# Mapping Transcriptomic Vector Fields of Single Cells

**DOI:** 10.1016/j.cell.2021.12.045

**Published:** 2022-02-01

**Authors:** Xiaojie Qiu, Yan Zhang, Jorge D. Martin-Rufino, Chen Weng, Shayan Hosseinzadeh, Dian Yang, Angela N. Pogson, Marco Y. Hein, Kyung Hoi (Joseph) Min, Li Wang, Emanuelle I. Grody, Matthew J. Shurtleff, Ruoshi Yuan, Song Xu, Yian Ma, Joseph M. Replogle, Eric S. Lander, Spyros Darmanis, Ivet Bahar, Vijay G. Sankaran, Jianhua Xing, Jonathan S Weissman

**Affiliations:** 1 Whitehead Institute for Biomedical Research Cambridge, MA, USA.; 2 Howard Hughes Medical Institute, Massachusetts Institute of Technology, Cambridge, MA, USA.; 3 Department of Computational and Systems Biology, University of Pittsburgh, Pittsburgh, PA, USA.; 4 Joint CMU-Pitt Ph.D. Program in Computational Biology, University of Pittsburgh, Pittsburgh, PA, USA.; 5 Broad Institute of MIT and Harvard, Cambridge, MA, USA.; 6 Division of Hematology/Oncology, Boston Children’s Hospital and Department of Pediatric Oncology, Dana-Farber Cancer Institute, Harvard Medical School, Boston, MA, USA.; 7 Department of Molecular and Cell Biology, University of California, Berkeley, CA, USA.; 8 Chan Zuckerberg Biohub, 499 Illinois St, San Francisco, CA 94158, USA; 9 Department of Electrical Engineering and Computer Science, Massachusetts Institute of Technology, Cambridge, MA 02139, USA; 10 Department of Mathematics, University of Texas at Arlington, Arlington, TX, USA.; 11 Lycia Therapeutics, South San Francisco, San Francisco, CA, USA.; 12 California Institute for Quantitative Biosciences, University of California, Berkeley, CA, USA.; 13 Microsoft, Redmond, WA, USA.; 14 Halıcıoğlu Data Science Institute, University of California San Diego, San Diego, CA, USA.; 15 Medical Scientist Training Program, University of California, San Francisco, CA, USA.; 16 Department of Systems Biology Harvard Medical School, Boston, MA 02125, USA.; 17 Department of Biology, Massachusetts Institute of Technology, Cambridge, MA 02139, USA.; 18 Genentech Inc., South San Francisco, CA, USA.; 19 UPMC-Hillman Cancer Center, University of Pittsburgh, Pittsburgh, PA, USA.; 20 Department of Physics and Astronomy, University of Pittsburgh, Pittsburgh, PA, USA.; 21 Koch Institute For Integrative Cancer Research at MIT, MIT, MA, USA.

**Keywords:** dynamo, hematopoiesis, RNA metabolic labeling, vector field reconstruction, differential geometry analysis, RNA Jacobian, acceleration, curvature, divergence and curl, cell fate transitions, dynamical systems theory, least action path, *in silico* perturbation

## Abstract

Single-cell (sc)-RNA-seq, together with RNA-velocity and metabolic labeling, reveals cellular states and transitions at unprecedented resolution. Fully exploiting these data, however, requires kinetic models capable of unveiling governing regulatory functions. Here, we introduce an analytical framework *dynamo*, that infers absolute RNA velocity, reconstructs continuous vector-field functions that predict cell fates, employs differential geometry to extract underlying regulations, and ultimately predicts optimal reprogramming paths and perturbation outcomes. We highlight *dynamo*’s power to overcome fundamental limitations of conventional splicing-based RNA velocity analyses to enable accurate velocity estimations on a metabolically-labeled human hematopoiesis scRNA-seq dataset. Furthermore, differential geometry analyses reveal mechanisms driving early megakaryocyte appearance and elucidate asymmetrical regulation within the PU.1–GATA1 circuit. Leveraging the Least-Action-Path method, *dynamo* accurately predicts drivers of numerous hematopoietic transitions. Finally, *in silico* perturbations predict cell-fate diversions induced by gene perturbations. *Dynamo* thus represents an important step in advancing quantitative and predictive theories of cell-state transitions.

## INTRODUCTION

A hallmark of metazoans is the ability of a single zygote to differentiate into a multitude of cell types while maintaining the same genome. To illustrate this process, Waddington introduced the epigenetic landscape, a metaphor in which differentiation proceeds like a ball sliding downhill into various valleys (Waddington, 1957). This metaphor has been used to intuitively explain cell differentiation (Huang et al., 2007), and more recently transdifferentiation or reprogramming (Cahan et al., 2014); however, a central goal of the field remains to move beyond such a qualitative, metaphorical conceptualization toward more quantitative, predictive models.

Mathematical modeling, especially in conjunction with dynamical systems theories (Brauer and Kribs, 2015), provides a powerful tool for gaining mechanistic insights into how gene regulatory networks (GRNs) control biological processes (Alon, 2006). In a dynamical systems formalism, one can represent the state of each cell as a vector (***x***) in a multi-dimensional expression space in which the elements are the instantaneous concentrations of molecules. Neglecting stochasticity, the time derivative of the cell state, or its velocity $\left(\dot{x}(t)\right)$, is governed by a set of ordinary differential equations (ODEs) determined by the underlying GRN, expressed as $\dot{x}(t)=f(x(t))$, where ***f*** is a vector field function of the instantaneous cell states (***x***(*t*)). Although efforts have been made to perform whole-cell simulations of bacteria (Karr et al., 2012; Macklin et al., 2020), it remains a grand challenge to reconstruct the vector field function representing the time evolution of a genome-wide expression state in mammalian cells from experimental data.

Recent developments in single-cell genomics have enabled profiling of cell state transitions at unprecedented resolution (Cao et al., 2020a). However, due to their destructive nature, it is generally infeasible to follow the same cell over time. Advances in single-cell profiling have fueled the development of computational approaches for inferring cellular dynamics from snapshot measurements. Chief among them are pseudotime-based methods (Bendall et al., 2014; Haghverdi et al., 2016; Saelens et al., 2019; Trapnell et al., 2014) first developed to infer the order of biological progression by learning a graph manifold of single cells based on transcriptome similarity. However, pseudotime ordering is limited to the analysis of central trends of biological progressions rather than the precise dynamics of individual cells over real time, and it is not generally suitable for resolving the directionality of biological processes (Qiu et al., 2020b). A second major advance has been the development of RNA velocity (La Manno et al., 2018), which predicts the cell RNA expression states in the near future by explicitly exploring the intrinsic splicing kinetics. Efforts have been made to extend “RNA velocity” to “protein velocity” (Gorin et al., 2020) or non-stationary states (Bergen et al., 2020). Such methods provide a view of the short-term evolution of individual cell states, but have intrinsic limitations (see STAR Methods) that prohibit it from accurately predicting the continuous evolution of cell states over a long period of time.

Recently, several groups have adapted bulk RNA-seq with metabolic labeling to single-cell approaches (Battich et al., 2020; Cao et al., 2020b; Erhard et al., 2019; Hendriks et al., 2019; Qiu et al., 2020a). The ensuing ability to obtain time-resolved scRNA-seq, or *tscRNA-seq*, provides further quantitative measures of cell state and its velocity by distinguishing “new” and “old” RNA molecules in an experimentally programmable manner. Thus, these methods in principle provide the data necessary for accurate reconstruction of transcriptomic vector fields. However, mathematical models and tools for integrating labeling-based tscRNA-seq and splicing-based conventional scRNA-seq, or *cscRNA-seq*, to allow one to properly estimate RNA turnover rates and infer RNA velocity remain undeveloped, as do methods for using such information to construct continuous vector fields. Finally, it remains unknown whether it is possible to leverage vector field functions to gain quantitative, predictive, and functionally important insights into cell state transitions, and if so, how. Thus, despite striking advances in single-cell profiling, our ability to fully exploit these measurements is limited by the lack of an appropriate analytical framework for interpreting the data and guiding future experiments.

Here, we introduce a framework for constructing and interpreting single-cell transcriptomic vector fields. The framework delivers four innovations. First, by reconciling RNA metabolic labeling and intrinsic splicing kinetics, we build an inclusive model of expression dynamics that not only accurately estimates genome-wide RNA turnover rates, but also overcomes the intrinsic limitations of conventional splicing-based RNA velocity to infer absolute velocities. Second, we develop a general algorithm for robustly reconstructing the continuous transcriptomic vector field functions from discrete, sparse, noisy single-cell measurements. Third, we marry the scalability of machine learning-based vector field reconstruction methods with the interpretability of differential geometry analyses, including Jacobian, acceleration, curvature, and divergence, to gain further biological insights. Fourth, leveraging the analytical vector field function reconstructed directly from scRNA-seq datasets, we develop two principled methods, Least Action Paths (LAPs) and *in silico* perturbation, to make non-trivial predictions of optimal paths and key drivers of cell fate transitions, as well as outcomes of genetic perturbations.

This framework represents a notable advance from the metaphor of epigenetic landscape to a quantitative and predictive theory of the time evolution of single cell transcriptomics, applicable to many biological systems and at genome-wide scale. We have made the associated computational framework as an open-source software, ***dynamo***, available at https://github.com/aristoteleo/dynamo-release.

## RESULTS

### A general framework for cell state transitions with vector field function and differential geometry analyses

**Figure 1:**
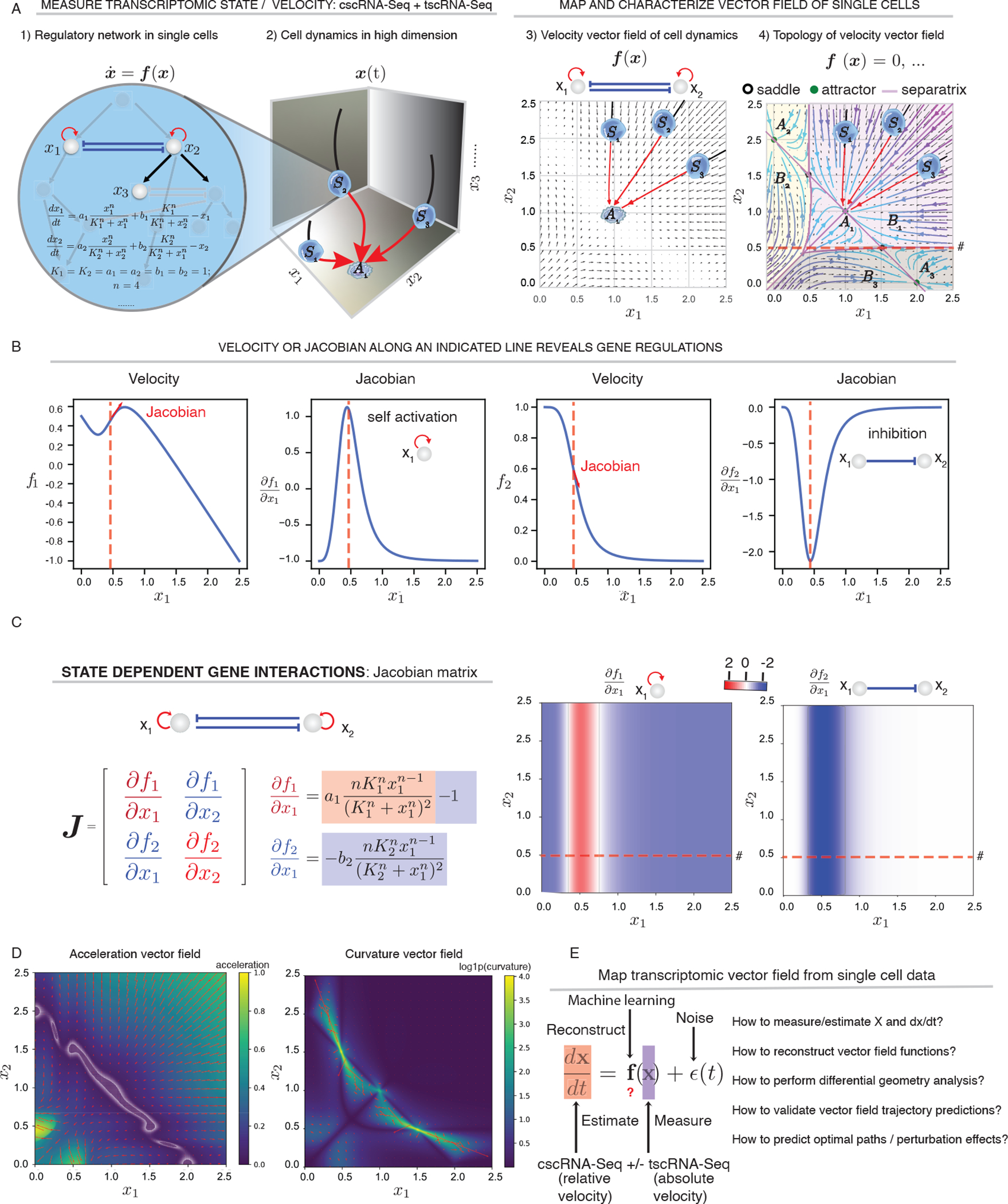
Modeling single-cell expression dynamics using velocity vector field functions and differential geometry analyses. A. **Cell state transition under dynamical systems framework. 1**) The toggle-switch motif of two genes (whose instantaneous expression levels are denoted as *x*_1_ and *x*_2_) and one of their downstream targets, *x*_3_, are embedded in an unknown complex regulatory network. **2**) Cell fate transitions as trajectories in a high-dimensional state space spanned by state descriptors. Here a three-dimensional state space is used to reveal the dynamics of the highlighted three-gene system from **1**. Any point in this space represents a network state *S*(*t*) = (*x*_1_, *x*_2,_ *x*_3_) at time *t*. Three example states *S*_1_, *S*_2_, and *S*_3_ and their convergent trajectories toward the same stable attractor state, *A*_1_, are shown. **3**) Global view of cell dynamics via vector field functions. **4**) Topological features of the vector field. Important features include: steady states, and saddle points, attractor basins, separatrices, and nullclines. Definition of these features can be found in STAR Methods. The vector field function of genes *x*_1_ and *x*_2_ is included in **1)** (Qiu et al., 2012) B. **Velocity and Jacobian along the dashed line indicated in A4.** Calculating the derivative of the velocity of *x*_1_, *f*_1_ (1th-panel) or that of *x*_2_, *f*_2_ (3th-panel) along the indicated line gives rise to the Jacobian terms *J*_11_ (self-activation of gene *x*_1_) or *J*_21_ (inhibition of *x*_2_ by *x*_1_). C. The Jacobian (**left**) of a vector field function reflects state-dependent gene interactions in the state space, represented as a heatmap (**right**). D. Acceleration and curvature vector fields of single-cell gene expression. Color of the heatmaps corresponds to the length of the acceleration and curvature vectors at each point in the state space. Quivers correspond to the acceleration or curvature vectors. For **C**/**D**, see more details in Box 1. E. Summary of the task of mapping vector field functions from transcriptomic data, formulated as a machine learning problem, with downstream validations, analyses and predictions.

In principle, a velocity vector field (Box 1) provides a complete description of how genes regulate each other. As a simple example, consider a two-gene toggle-switch motif (Huang et al., 2007; Wang et al., 2010) that appears frequently in cell differentiation such as the PU.1/SPI1-GATA1 regulatory network involved in hematopoiesis (Figure 1A1). The vector field function for this motif is often formulated as a set of ODEs (Figure 1A1), which model the self-activation and mutual inhibition involving PU.1 and GATA1, specify the instantaneous velocity of a cell at any given expression state, and predict the evolution of the cell state over time (Figure 1A2–4). One can further characterize the topology of this vector field in its gene expression space with separatrices that divide the space into three attractor basins, each containing a stable fixed point (the attractor) corresponding to a stable phenotype (Figure 1A4). We illustrate three representative cells that start from different states of the same attractor basin of attractor *A*_1_, each propagating along a trajectory (streamline) defined by the vector field function to settle at the same attractor state *A*_1_ (Figure 1A2–4, Figure S1A). By contrast, saddle points are unstable fixed points located on sepatrices connecting pairs of attractors (Figure 1A4).

Analyses of the vector field can also help generate hypotheses about how genes regulate cell states (Box 1, Figure 1B, C). For example, the Jacobian can be used to investigate the cell state–dependent interactions because it is tightly related to the underlying regulatory network (Box 1). In the toggle-switch model, the Jacobian analysis correctly identifies self-activation and mutual inhibition, with the strongest regulation taking place when *x*_1_ and *x*_2_ are about 0.5 (Figure 1B, C, Figure S1B).

A number of additional differential geometric quantities provide complementary information of gene regulations. The **acceleration** field (Box 1, Figure 1D left) reveals gene expression subspaces (i.e., hotspots of cells states) where the velocities change dramatically, either in magnitude or direction, e.g., the two symmetric regions in the bottom left corner where the expression level of either *x*_1_ or *x*_2_ increases rapidly. When a cell leaves an unstable state (e.g., a progenitor) and moves toward a stable attractor state (e.g., a mature cell type), its velocity tends to increase before it slows down in the attractor state (Figure 1D left). Therefore, it is possible to detect genes that have a large value for acceleration (in magnitude) in progenitor states, making key contributions to cell fate commitment, long before cells exhibit discernible lineage-specific gene expression differences. A related but different quantity is the **curvature** field (Box 1, Figure 1D right), which reveals gene expression hotspots where the velocity changes direction abruptly, e.g., in regions around unstable fixed points where one or more genes’ expression changes from induction to repression or *vice versa* (Figure 1D right, see especially the regions coincide with the two saddle points). The genes that strongly contribute to the curvature are regulatory genes that steer the cell fate. Curl and divergence (Box 1, Figure S1C), respectively, characterize the infinitesimal rotation of a cell state in the vector field and the local flux exiting versus entering an infinitesimal region in the expression space – the “outgoingness”. The sources (sinks) of a dynamical system often have strong positive (negative) divergence. Thus, divergence of single cells can be used to identify the possible progenitors (sources) or terminal cell types (sinks) of a differentiation system.

The toggle-switch motif illustrates the significance of vector fields and various differential geometry analyses in studying the dynamics of a regulatory network. However, such simplified motifs are embedded within an unknown genome-wide regulatory network (Figure 1A). Thus, it is desirable to apply machine learning methods to reconstruct the transcriptomic vector field functions directly from single-cell measurements (Figure 1E).

### An integrative model of RNA metabolic labeling and expression kinetics provides genome-wide estimates of mRNA kinetic parameters

The original RNA velocity method (La Manno et al., 2018) uses incidentally captured intron reads from cscRNA-seq data and assumes a universal splicing rate constant. Assuming a steady state for cells with extreme high expressions, and using the substitution $\tilde{\gamma }=\gamma /\beta$ (*β* and *γ* are the respective rate constants for splicing and degradation), the conventional RNA velocity as defined in the original study (La Manno et al., 2018) is given by (see more details in STAR Methods):

$$v=u-\tilde{\gamma }s.$$

Here *u* and *s* are the copies of unspliced and spliced RNAs for a particular gene in the cell, The resultant degradation rate constants and velocities from conventional RNA velocity method are therefore relative, and scaled by the gene-specific splicing rate constant *β* (See STAR Methods). We reason that such limitations can be relaxed with tscRNA-seq, which measures RNA turnover dynamics in a controllable, less biased, and time-resolved fashion.

**Figure 2:**
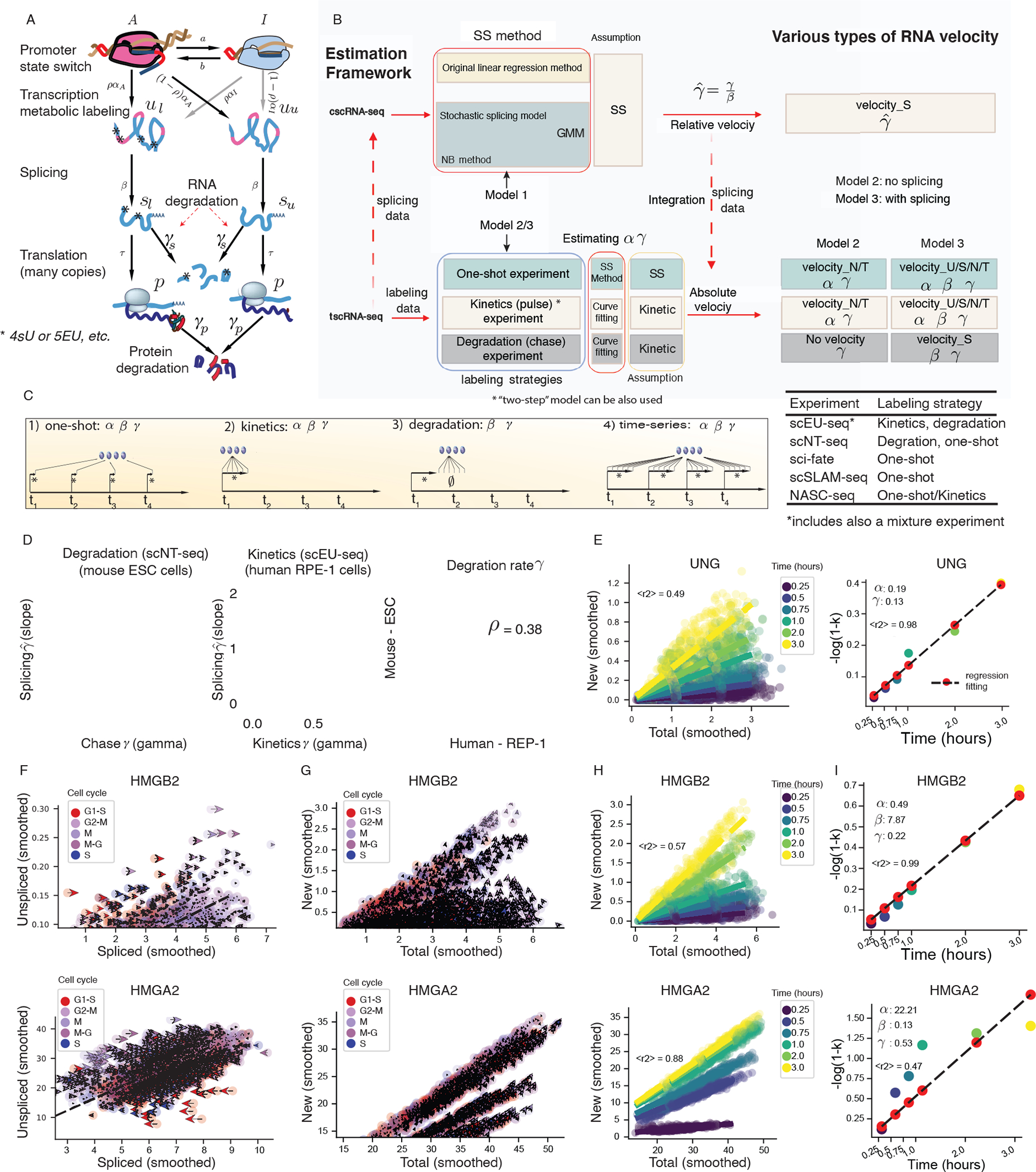
Inclusive model of single-cell expression dynamics incorporates RNA metabolic labeling. A. A comprehensive model of expression kinetics that includes promoter state switch, metabolic labeling, transcription, splicing, translation, and RNA/protein degradation. *A* and *I* correspond to active and inhibitive promoter states, whereas *ρ* is the fraction of labeled RNA (STAR Methods). *u*_*u*_, *u*_*l*_, *s*_*u*_, and *s*_*l*_ are respectively unspliced unlabeled, unspliced labeled, spliced unlabeled, and spliced labeled RNA. B. ***Dynamo***’s estimation framework of kinetic parameters for tscRNA-seq and cscRNA-seq experiments. GMM: generalized methods of moments; NB: negative binomial; SS: steady state. C. Typical RNA metabolic labeling strategies and their applications. On the left, **One-shot** experiment, an experiments with a single RNA labeling period; **kinetics** experiment, a time-series of multiple durations of RNA labeling; **degradation** experiment, a time-series with an extended RNA labeling period, followed by chase at multiple time points; **Multi-time-series** experiment, single cell samples are collected at multiple time points, each with a kinetics experiment. The table on the right summarizes the main labeling strategies used in published tscRNA-seq studies. D. Comparing degradation rate constants (*r*) calculated from tscRNA-seq data and the relative degradation rate constants $\left(\tilde{\gamma }\right)$ from the corresponding splicing data, and those from human cells or mouse cells. Each point corresponds to a gene. E. Two-step method (see STAR Methods) of the kinetics experiment [data from scEU-seq study (Battich et al., 2020)]: **step 1**) A strong linearity in the new–total RNA phase plane of gene *UNG* with ascending slope *k* for longer labeling times; **step 2**) A strong linearity between −ln(1 − *k*) and labeling time period *t* for the *UNG* gene. Color of data points (**right**) corresponds to the experimental time, as on the **left**. The same applies to panel **I**. F. Phase portraits of spliced-unspliced RNA planes of *HMGB2* and *HMGA2*. Quivers correspond to the spliced RNA velocity. G. Same as above but for the total–new RNA planes. Quivers correspond to the total (*x*-component) or new (*y*-component) RNA velocity. H. **Step 1** as in panel **E** but for genes *HMGB2* and *HMGA2*. I. **Step 2** as in panel **E** but for genes *HMGB2* and *HMGA2*. Panels **F-I** all used the kinetics experiment dataset from scEU-seq study (Battich et al., 2020)

To develop a unified framework for extracting RNA kinetic information from cscRNA-seq and tscRNA-seq datasets, we constructed an inclusive model (Figure 2A) that considers RNA metabolic labeling (when using tscRNA-seq data), RNA splicing and degradation. To account for different data types and experiments, we further implement three reduced models: **Model 1** considers RNA transcription, splicing and degradation, but not RNA metabolic labeling, and is tailored for cscRNA-seq, whereas both **Models 2** and **3** are tailored for tscRNA-seq with metabolic labeling, with the difference that only **Model 3** considers RNA splicing (Figure SI2A).

When only cscRNA-seq data are available, or when one needs to use splicing data from tscRNA-seq experiments, ***dynamo*** can be used to estimate the relative degradation rate constant $\left(\tilde{\gamma }=\gamma /\beta \right)$ and relative spliced RNA velocity (Figure 2B, top). The estimation methods built upon **Model 1** from Figure S2A include both the original method (La Manno et al., 2018) and the generalized method of moments (GMM) (Hansen, 1982). The GMM, in turn, consists of the stochastic splicing method, which relies on a master equation formulation of RNA kinetics (see STAR Methods) and is equivalent to the stochastic method developed recently (Bergen et al., 2020), and a new approach, the negative binomial (NB) method, which additionally models the gene expression at steady state as a NB distribution, in the same vein as reported in (Grün et al., 2014).

By comparison, from a tscRNA-seq experiment, one can estimate the absolute kinetic parameters (*α*, *β*, and *γ*) and calculate absolute unspliced, spliced, new, or total RNA velocity (Figure 2B, bottom). We suggest three general labeling strategies, namely one-shot, kinetics/pulse, and degradation/chase experiments, aimed at estimating different RNA kinetic parameters (Figure 2C). It is possible to extend or combine these general labeling strategies to more complicated labeling schemes, e.g., the fourth type in Figure 2C, which consists of a time-series of multiple kinetics experiments, or a mixture experiment as in the scEU-seq study (Battich et al., 2020).

Estimating the parameters and RNA velocities with labeling data involves some technical subtleties, which we took into account when developing the corresponding algorithms. Overall, we estimate absolute splicing and degradation constants (*β* and *γ*) by first estimating the degradation rates from labeling data and then the scaled degradation rate constant $\left(\tilde{\gamma }=\gamma /\beta \right)$ from splicing data, followed by obtaining an absolute splicing rate constant $\beta =\gamma /\tilde{\gamma }$ (See STAR Methods for details). For kinetics experiments, we designed a two-step method (see STAR Methods, Figure 2D–I).

To demonstrate the effectiveness of our approach, we applied our framework to two previously reported datasets: a degradation dataset obtained by scNT-seq of murine ESCs (Qiu et al., 2020a) and a kinetics dataset obtained by scEU-seq of RPE-1 cells (Battich et al., 2020) (Figure 2D–I and Figure S2B–I). In both datasets, the values of *γ* estimated from the degradation experiment, or those from the kinetics experiment using the two-step method, show no apparent correlation with $\tilde{\gamma }=\gamma /\beta$ (splicing rate is not a universal constant) (Figure 2D, left and middle). Unsurprisingly, the splicing rates are generally much higher than the degradation rates (Figure SI2B left and middle, D). Still, certain genes have extremely fast degradation rates (Figure SI2B left and middle, D). For example, *Slc25a32* degrades quickly, with a half life (*t*_1/2_=ln 2/*γ*) of just 14 minutes, 81 times faster than *Ank2* (*t*_1/2_ of 18.6 hours) (Figure SI2C). Housekeeping genes tend to be spliced quicker but degraded slower than other genes (Figure SI2E).

In the scEU-seq cell-cycle data (Battich et al., 2020), genes with either fast splicing or fast degradation rates were enriched in cell-cycle–related pathways (Figure SI2F). Interestingly, splicing and degradation rates of mouse genes are correlated with, but generally higher than, those of their human orthologs (Figure 2D right, Figure S 2B right), similar to what has been observed previously (Matsuda et al., 2020; Rayon et al., 2020). In particular, the new and total RNAs show the expected strong linear relationship, with slope increasing with the labeling time during the kinetics experiment (Figure 2E, G–I; see also STAR Methods). Interestingly, analysis of the transcription and degradation rates for the mixture experiment (Battich et al., 2020) (Figure SI2G–I) revealed that the genes with the highest transcription rates are all mitochondrially encoded (Figure SI2H).

For a kinetics experiment, we can plot the unspliced/spliced velocity on the “phase plane” (La Manno et al., 2018) of spliced and unspliced RNAs, as well as the new/total velocity on the “phase plane” of total and new RNAs. For example, from the phase plot, we find that since the splicing rate of *HMGB2* is greater than its degradation rate, across cells its unspliced RNA is less abundant than its spliced RNA (Figure 2F, top row). By contrast, *HMGA2* exhibits the opposite dynamics (Figure 2F, bottom row). The new RNA velocities are always non-negative, as the levels of labeled RNAs generally increase during a short labeling experiment (Figure 2G).

### RNA metabolic labeling with *dynamo* overcomes fundamental limitations of conventional splicing-based RNA velocity

**Figure 3:**
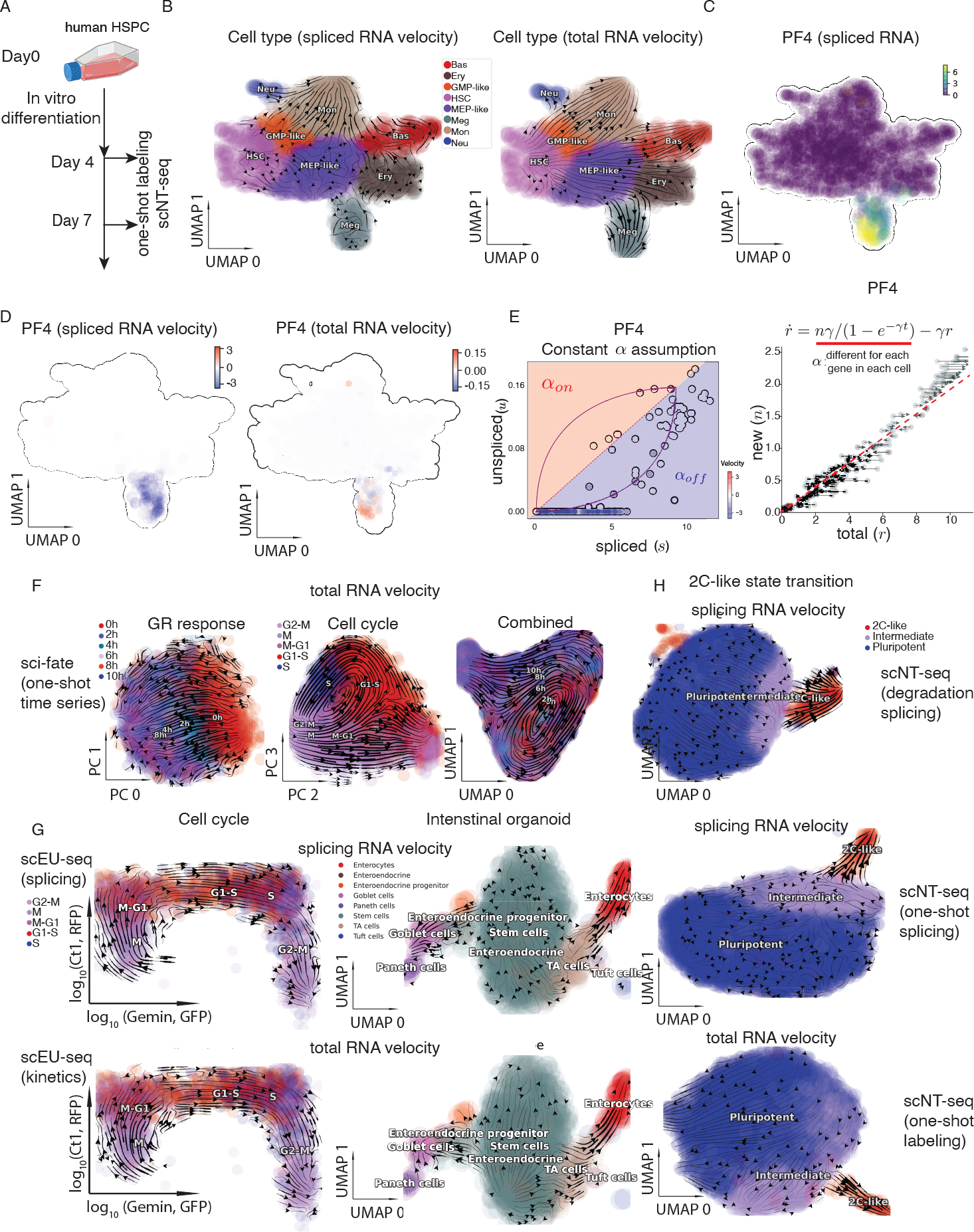
Metabolic labeling experiments improve and generalize RNA velocity estimation. A. Schematic of the one-shot labeling scNT-seq experiment for human hematopoietic stem and progenitor cells (HSPCs) (STAR Methods). B. RNA velocity flow projected in the UMAP space. **Left**: splicing data give noisy, nonsensical velocity flow with terminal cell types moving back to progenitors. scVelo’s dynamical model (Bergen et al., 2020) was used to generate this figure (see more at Figure S3C). Right: ***Dynamo*** analysis of the labeling data reveals a smooth transition of HSCs into MEP-like and GMP-like cells, which further ramify into Meg/Ery/Bas lineages and Mon/Neu lineages, respectively. C. Gene expression distribution of *PF4*, an Meg lineage marker, across cells. D. Velocity magnitude of *PF4* across cells. **Left**: spliced RNA velocities based on splicing data. **Right**: total RNA velocities based on labeling data with ***dynamo***’s estimation framework. E. Phase plot of gene *PF4*. **Left**: Splicing RNA phase plot. Because of unsuccessful capture of unspliced RNA and a rapid increase of transcription rate in the Meg lineage, the majority of cells are mistakenly treated as if they are in the repression phase with negative velocity. **Right**: Labeling RNA phase plot. Quivers correspond to the total RNA velocity. With labeling data under ***dynamo***’s framework, the transcription rate is modeled as a variable that depends on new RNA (*n*) which is measured in an unbiased manner for each gene in each cell (STAR Methods). F. Streamline plots of **one-shot** labeling dataset from (Cao et al., 2020b) reveal two orthogonal processes of GR response and cell cycle progression. From **left** to **right**: streamline plot on the first two principal components (PCs), the second two PCs, and the first two UMAP components that are reduced from the four PCs, respectively. G. Conventional (**top**) and kinetics labeling (**bottom**) velocity analysis of the RPE1-FUCCI cells (**left**) and murine intestinal organoid system (**right**) of the scEU-seq study. H. Conventional (**top, middle**) and **degradation** labeling (**bottom**) velocity analysis of the TET-dependent stepwise pluripotent–2C bidirectional transition of murine ESC in the scNT-seq study.

To demonstrate that large-scale, UMI-based tscRNA-seq datasets improve velocity analysis over cscRNA-seq datasets, using scNT-seq (Qiu et al., 2020a) we generated a time-resolved scRNA-seq dataset using primary human HSPCs (Martin-Rufino and Sankaran, 2021). Specifically, we applied scNT-seq to profile human CD34^+^ HSPCs undergoing multi-lineage differentiation in *in vitro* culture on days 4 and 7 (Figure 3A, Figure S3A, STAR Methods). We processed the data with ***dynast*** (STAR Methods) to quantify unspliced, spliced, new, and total RNA for each gene in each cell. We next performed cscRNA-seq RNA velocity analyses based solely on the splicing data (unspliced and spliced RNA). Unexpectedly, regardless of the tools or methods used, splicing RNA velocity analyses persistently led to nonsensical velocity flow starting in mature cell types [e.g., megakaryocyte (Meg), erythrocyte (Ery), or basophil (Bas) lineages] and pointing backward to progenitors, including Meg and Ery progenitor (MEP)-like/granulocyte (Gran) and monocyte (Mon) progenitor (GMP)-like cells and HSPCs (Figure 3B left, Figure S3B, C). By contrast, with ***dynamo***’s modeling framework, the labeling data (labelled and total RNA) yielded velocity flows that closely recapitulate the established knowledge of hematopoiesis (Figure 3B right). Previous studies have reported that biased capture of intron regions via mispriming in droplet-based scRNA-seq libraries (La Manno et al., 2018; Qiu et al., 2020a) and dynamic RNA transcription rates (Barile et al., 2021; Bergen et al., 2021) may result in inaccurate RNA velocity flow. Indeed, when inspecting the expression kinetics of lineage marker genes, such as *PF4*, a Meg lineage marker (Paul et al., 2016), we found that the spliced and unspliced RNAs were undetectable in progenitors, but its expression switched on rapidly in the Meg lineage (Figure 3C, left subpanels of Figure 3D, E) with the unspliced RNA present at a much lower level, consistent with the unsuccessful capture of its introns. By contrast, with metabolic labeling, the new RNA is measured in an unbiased manner, leading to continuous activation of new and total *PF4* RNA in the Meg lineage (right subpanels of Figure 3D, E).

In contrast to the implicit assumption of a constant transcription rate for cscRNA-seq data (Barile et al., 2021; Bergen et al., 2020; La Manno et al., 2018), ***dynamo*** models the transcription rate for labeling data as a variable that depends on measured new RNA and can therefore vary across genes and cells. Collectively, the unbiased measurements of the nascent RNA and the modeling assumption of a transcription rate that differs for each gene in each cell correctly led to positive velocities of *PF4* for Meg lineage cells and more broadly corrected the velocity flow (Figure 3B, E). For the cscRNA-seq data, we reasoned that by providing the lineage relationships as a prior, one may correct spliced RNA velocity by identifying and removing genes whose behavior in the phase plane is inconsistent with those relationships (Figure S3D). Indeed, we found a large fraction of genes whose behaviors disagree with the known hematopoietic lineage hierarchy (Figure S3E, F). After removing those genes from the velocity analysis, we obtained a velocity flow (Figure S3G) that approaches the one generated using the labeling data (right subpanel of Figure 3B). The downstream analysis, however, remains restricted because there are a small number (43) of reliable genes and the velocity magnitude is relative; moreover, the procedure prevents discovery of new lineage relationships. We further demonstrate the generality of ***dynamo*** with labeling data (Figure SI3H–J) in overcoming the intrinsic limitations in splicing RNA velocity estimation, based on an analysis with the neuronal activity dataset from (Qiu et al., 2020a).

To assess ***dynamo***’s ability to deconvolve orthogonal cellular processes, we analyzed datasets from sci-fate in which cell cycle progression and glucocorticoid receptor (GR) activation are explored (Cao et al., 2020b). We reanalyzed this dataset and performed time-resolved total RNA velocity analysis on combined or individual set(s) of GR response and cell-cycle genes detected by the original study. From the analysis with GR response gene set, we revealed a smooth sequential transition from untreated cells at time point 0 to 2, 4, 6, and 8 hours after the initial DEX (dexamethasone) treatment (Figure 3F/S3K GR response). Similarly, we identified a cyclic loop matching the cell-cycle progression from the analysis with the cell-cycle gene set (Figure 3F/S3K Cell cycle). Interestingly, combined analysis revealed both a linear progression of the GR response and a circular loop indicative of cell cycle (Figure 3F/S3K combined). Next, we analyzed datasets from the scEU-seq study (Battich et al., 2020) and observed a sequential cell-cycle transition for the RPE1-FUCCI cells (Figure 3G, left column) as well as a bifurcation (Figure 3G right column) from intestinal stem cells into the secretory lineage (left) and the enterocyte lineage (right) for the intestinal organoid data. Similarly, labeling based RNA velocity analyses accurately revealed an increase of the commitment into rare 2C-like totipotent cells under *Tet 1/2/3* triple knockout (TetTKO) on the scNT-seq mESC dataset (Figure 3H/S3 L, M) from (Qiu et al., 2020a).

### Accurate, robust and efficient reconstruction of vector field functions of single cells

We next sought to leverage the discrete and local measures of velocity samples to reconstruct continuous vector field functions in the full gene expression state space. We start with a theoretical discussion of the recoverability of vector field functions (Figure S4A and STAR Methods) (Kim et al., 2000; Weinreb et al., 2018) and validated that scRNA-seq contains sufficient information for the reconstruction with a dataset where transcriptomes of sister/cousin cells are profiled (Figure S4B–F and STAR Methods).

**Figure 4.**
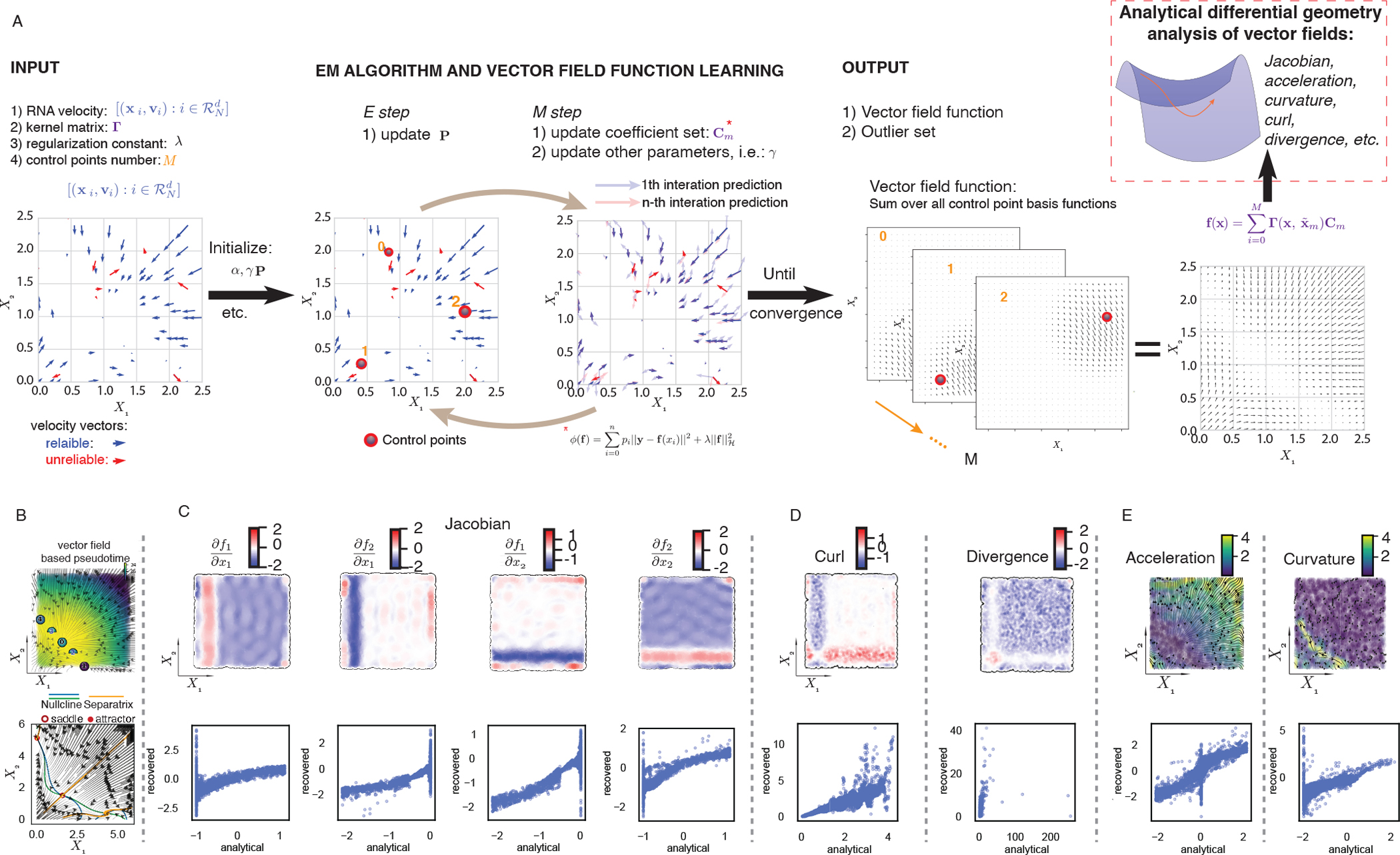
Mapping the vector field, quantifying its topography, and moving towards differential geometry analyses. A. Functional reconstruction of the continuous and analytical velocity vector field from sparse, noisy single cell velocity measurements with sparseVFC (Ma et al., 2013) (Box 2, STAR Methods and STAR Methods). B. Reconstructed vector field and topological features of the simulated toggle-switch system. **Top**: Scatterplots of simulated cells (*x*/*y*-axis: expression of *x*_1_/*x*_2_, same as in **C**) that are colored by vector-field based pseudotime, calculated via the *ddhodge* algorithm (Maehara and Ohkawa, 2019). Full-cycle nodes correspond to attractors while half-cycle saddle points. Streamline plot of the reconstructed vector field is superimposed on top of the scatterplot. **Bottom**: *x*/*y*-nucline and separatrix, plotted on top of the streamline plot of the reconstructed vector field. C. Scatterplots of simulated cells with a frontier representing the expression boundary of sample cells (**top**). Cells are colored by the estimated values of the indicated Jacobian elements. **Bottom**: Scatterplots comparing the estimated (*x*-axis) and analytical (*y*-axis) Jacobian elements across cells. D. Same as in **C** but for the recovered curl and curvature. E. Same as in **C** but for the acceleration and curvature. Since acceleration and curvature are vectors, the streamlines of the recovered acceleration and curvature vector field are visualized. Cells are colored by the length of acceleration or curvature vectors.

In general, to construct the vector field function from noisy and sparse samples of single-cell states and velocity estimates (Figure 1A), we adopted a machine learning approach that takes advantage of recent advances in vector-valued function approximation to scalably, efficiently, and robustly learn the transcriptomic vector field (see Box 2). The framework, as outlined in Figure 4A, employs sparseVFC (sparse approximation of Vector Field Consensus) (Ma et al., 2013), which uses a vector-valued kernel method built on RKHS (reproducing kernel Hilbert space) to learn the vector field, which is expressed analytically as a weighted linear combination of a set of vector-valued kernel basis functions (Figure 4A Output). The learning process relies on sparse approximation to estimate the coefficients (weights) of a selected number of basis functions, each associated with a control point, that is often much smaller than the number of data points (Figure 4A Output). With sparse approximation, the reconstruction scales linearly with the number of data points in both computational time and memory requirements (Ma et al., 2013). To account for the noise and outliers of velocity measurements, sparseVFC relies on an **EM algorithm** to iteratively optimize the set of inliers as well as the optimized coefficient set for each basis function (Figure 4A), further improving the robustness of vector field reconstruction. With the continuous vector field function that is learned in either high-dimensional PCA space, which can be projected back to the full transcriptomic space, or lower dimensional space (such as 2D UMAP space), or directly in the full gene-expression space, we can also calculate RNA Jacobian, acceleration, curvature, divergence, curl, etc, with derived analytical formulas (Figure 4A).

To explore the potential of the vector field reconstruction, we first tested the efficacy of our reconstruction on a simulation dataset with 5,000 randomly sampled points on the state space of the model introduced in Figure 1. The estimated streamlines of the reconstructed vector field, as well as the fixed points, etc., were nearly indistinguishable from the analytical ones (Figure 4B). Moreover, we could accurately recover the Jacobian matrix across the state space (Figure 4C, Figure S4G). The estimated higher-order vector calculus quantities closely matched the true analytically computed quantities (Figure 4D, E, Figure S4G). The analytical formulae of vector calculus that we derived lead to nearly 1000-fold speedup than state-of-the-art numerical approaches (Figure SI4H).

We also tested the idea of using the scalar potential estimated from a reconstructed vector field with the Hodge decomposition as a new type of pseudotime analysis (Maehara and Ohkawa, 2019). Because this method utilizes velocity fields that consist of the direction and magnitude of expression kinetics, it is intrinsically directional and arguably more relevant to real time than other pseudotime methods. As expected, the vector field–based pseudotime revealed a smooth cell state transition moving toward attractor states (Figure 4B bottom). We further demonstrated the robustness of vector field reconstruction under cell downsampling, noise distortion as well as with respect to changes in its parameters (Figure S4I–k).

Once a vector field is learned, one immediate application is to predict the historical or future state of a cell in a manner analogous to Newtonian mechanics, i.e., with the vector field function and the initial gene expression states, one in principle can predict position and velocity at any point in time (Supplementary Animation). We reason that this prediction can be validated by comparing the single-cell trajectory prediction with gene expression in clonal cells (cells arising from the same progenitor through cell division) measured sequentially, which approximates the dynamics of a single cell over time (Figure S5A). We firstly generated such a dataset from an experiment in which we sequentially profiled a statically barcoded human leukemia cell line (HL60) with scSLAM-seq under ATRA (all-trans-retinoic acid)-induced neutrophil lineage commitment (Huang et al., 2005). We show that the vector field reconstructed for this dataset can predict the single cell fate trajectories over several days (Figure S5B–F). We also arrive at similar conclusions when analyzing data from a recently published study (Weinreb et al., 2020) with sequential clone cell tracing (Figure S5G–I).

### Differential geometry analyses reveal timing and regulatory mechanisms of hematopoiesis

**Figure 5:**
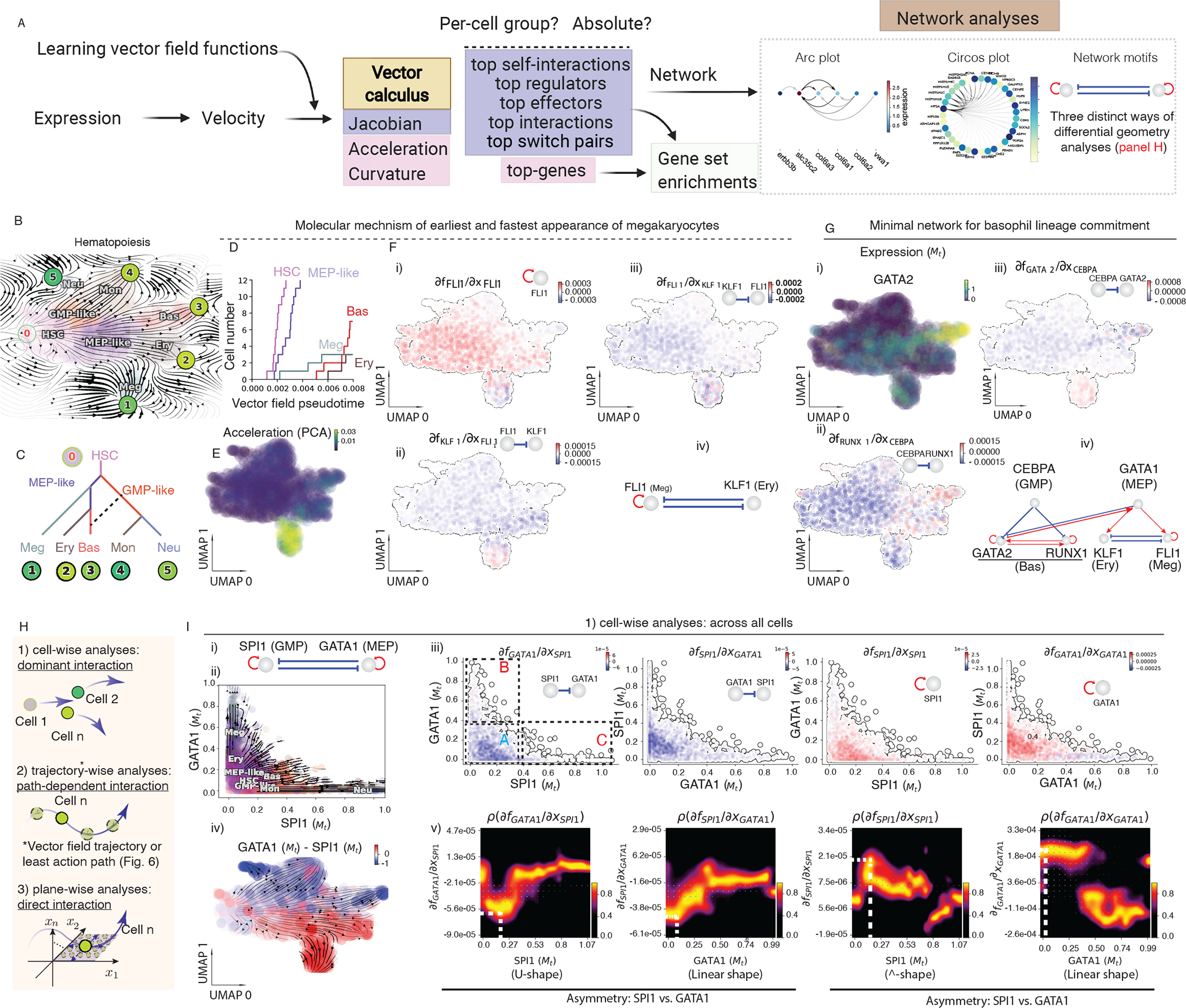
Vector field and differential geometry analyses of human hematopoiesis. A. Schematic of leveraging differential geometry quantities to rank genes (using either raw or absolute values) across all cells or in each cell group/state, followed by gene set enrichment, network construction, and visualization. Furthermore, ***dynamo*** can identify top toggle-switch pairs driving cell fate bifurcations. B. The reconstructed vector field and associated fixed points. The color of digits in each node reflects the type of fixed point: **red**, emitting fixed point; **black**, absorbing fixed point. The color of the numbered nodes corresponds to the confidence of the fixed points. C. Lineage tree of hematopoiesis, lumped automatically from the vector field built in the UMAP space (STAR Methods). D. Megakaryocytes appear earliest among the Meg, Ery, and Bas lineages. The vector field pseudotime is calculated based on the velocity transition matrix, as in Figure S6A. E. Megakaryocytes have the largest acceleration among all cell types. F. Molecular mechanisms underlying the early appearance of the Meg lineage. i) Self-activation of *FLI1*. ii) Repression of *KLF1* by *FLI1*. iii) *FLI1* represses *KLF1*; iv) Schematic summarizing the interactions involving *FLI1* and *KLF1*. G. Regulatory network governing the Bas lineage’s dual origins. i) *GATA2* has high expression in the Bas lineage; ii) *CEBPA* represses *RUNX1*; iii) *CEBPA* represses *GATA2*; iv) A minimal network governing GMP vs. Bas origin of Bas lineage (Figure S6I). H. Three approaches for in-depth network motif characterizations: 1) cell-wise analyses to reveal dominant interactions across all cells; 2) trajectory-wise analyses reveal trajectory dependent interactions along a trajectory (predicted either from vector field streamline, or least action path, see Figure 6). 3). Plane-wise analyses reveal direct interactions for any characteristic cell states by varying genes of interest while holding all other genes constant. I. Cell-wise analyses of the *PU.1*/*SPI1–GATA1* network motif across all cells. i) Schematic of the *SPI1*-*GATA1* toggle switch model. ii) Streamline plot of the RNA velocities of *SPI1* (*x*-axis) and *GATA1* (*y*-axis). iii) Repression from *SPI1* to *GATA1*, *GATA1* to *SPI1*, and self-activation of *SPI1*, and *GATA1*, in the *SPI1* and *GATA1* expression space. In particular, the repression from *SPI1* to *GATA1* is mostly discernable in progenitors (**rectangle A**) but becomes negligible when either *GATA1* is much higher than *SPI1* (**rectangle B**) or *GATA1* is close to zero (**rectangle C**). iv) *GATA1* has overall lower expression in the HSC state than *SPI1*. v) Similar to iii) but replaced with a *response* heatmap (Qiu et al., 2020b). White dashed lines indicate the minimum or maximum of repression or activation and the corresponding expression threshold.

Having demonstrated the validity of single-cell trajectory prediction, we next designed a coherent suite of differential geometric analyses with the vector field function to uncover quantitative information about gene regulation (Figure 4A, 5A). We then applied such analyses to our hematopoiesis tscRNA-seq dataset to gain mechanistic insights. We first learned the vector field function with this dataset. Fixed points identified in the UMAP space-based vector field (STAR Methods) correctly reflect the topology of the system (Figure 5B). The vector field was then organized into a tree structure (STAR Methods) that correctly summarizes the hematopoietic lineage hierarchy (Figure 3B, Figure 5B,C).

One intriguing phenomenon observed in hematopoiesis is that commitment to and appearance of the Meg lineage occurs more rapidly than other lineages (Sanjuan-Pla et al., 2013; Yamamoto et al., 2013). However, the mechanisms underlying this process remain elusive. To mechanistically dissect this finding, we focused on all cell types derived from the MEP-like lineage. The Meg lineage appears ahead of Ery and Bas lineages along the vector field based pseudotime axis (Figure 5D, SI6A). Interestingly, this early appearance of Meg lineage is further reinforced by its considerably higher RNA speed (Figure SI6B) and acceleration (Figure 5E) relative to all other lineages. When inspecting the expression of *FLI1* and *KLF1* (Siatecka and Bieker, 2011), known master regulators of Meg and Ery lineages, respectively, we observed high expression of *FLI1*, rather than *KLF1*, beginning at the HSPC state (Figure S6C). Furthermore, Jacobian analyses revealed mutual inhibition between *FLI1* and *KLF1* (Figure 5F) and self-activation of *FLI1* (Truong and Ben-David, 2000), but not *KLF1*, across all cells. These analyses collectively suggest self-activation of *FLI1* maintains its higher expression in the HSPC state, which biases the HSPCs to first commit towards the Meg lineage with high speed and acceleration, while repressing the commitment into erythrocytes through inhibition of *KLF1*.

Early studies reported that basophils are derived from GMPs (Iwasaki et al., 2006; Truong and Ben-David, 2000). Our dataset, however, suggests that they mostly originated from MEP-like cells (with a minor route from GMP-like cells) (Figure 3B, 5B, C), in line with recent scRNA-seq studies (Drissen et al., 2016; Pellin et al., 2019). To reconcile the discrepancy of two alternative trajectories of the Bas lineage, we next set to derive a minimal network model of its commitment. In order to identify the putative drivers of the Meg/Ery vs. Bas lineage bifurcation, we performed switch gene-pair analyses to identify gene pairs with antagonistic interactions (Figure 5A) for cells near the regions of the Ery and Bas lineage bifurcations. Notably, the *GATA2–PLEK* pair ranked first (among 17,556 pairs, Table S1). Similar to *PLEK*’s exclusivity in megakaryocytes (Figure S3B), the expression of *GATA2* is specifically high in basophils (Figure 5G i). Switch pair ranking analyses also prioritized the involvement of *CEBPA* and *RUNX1*, previously reported in (Guo et al., 2017), as well as *GATA1*, the master regulator of the GMP lineage. Subsequent Jacobian analyses indicated repression of *RUNX1* and *GATA2* by *CEBPA* (Figure 5G ii–iv), as well as mutual activation between *RUNX1* and *GATA2* and their self-activation in progenitors (Figure 5G iv, S6D, E). In addition, Jacobian analyses confirmed the known repression from *GATA1* to *GATA2*, activation from *GATA2* back to *GATA1*, as well as the activation of *KLF1* and *FLI1* by *GATA1* (Figure S6E). Collectively, these analyses reveal a network comprises the repression from key regulators of both GMP lineage and MEP lineage (*CEBPA* and *GATA1* respectively) to the basophil master regulators *GATA2* and *RUNX1*, suggesting that Bas lineage can arise via two potential trajectories, from either GMP or MEP cells, consistent with (Drissen et al., 2019).

To obtain mechanistic insights into key regulatory motifs from different perspectives, we developed three complementary strategies: cell-wise, trajectory-wise and plane-wise analyses (Figure 5H). We showcase these strategies with the canonical PU.1/SPI1-GATA1 network motif (Figure 5I i) (Guo et al., 2017; Huang et al., 2007). The streamlines of *SPI1* and *GATA1* show that HSPCs bifurcate into GMP-like and MEP-like branches (Figure 5I ii, SI6F). Most of the prior models for how SPI1 and GATA1 mutually inhibit each other assume the simplest additive model formalism (Guo et al., 2017; Huang et al., 2007), which was also adopted in Figure 1 and 4. A key characteristic of this form is that each Jacobian element is a function of only one of the two genes (Figure 1C, Figure S1B). Instead, the inhibitory effect of *SPI1* on *GATA1* (∂*f*_GATA1_/∂*x*_SPI1_) decreases as the level of *GATA1* increases (rectangle B of Figure 5I iii first subpanel), and the self-activation of *SPI1* (∂*f*_SPI1_/∂*x*_SPI1_) decreases with increased *GATA1* level (third subpanel of Figure 5I iii). Similar behaviors could also be observed for the reverse interactions (∂*f*_SPI1_/∂*x*_GATA1_ and ∂*f*_GATA1_/∂*x*_GATA1_). These behaviors are in marked contrast to Figure 1C, Figure S1B, but consistent with an alternative formalism in which *SPI1* and *GATA1* antagonize each other through a “competitive inhibition”-type mechanism, as reported in (Nerlov et al., 2000; Zhang et al., 2000).

Furthermore, regulation between SPI1 and GATA1 is typically modeled to be symmetric, with some sigmoidal functions reflecting cooperative binding (Guo et al., 2017; Huang et al., 2007). To extract quantitative insight into the regulatory functions of the motif (Figure 5I iii–iv), we first plotted distributions of the four Jacobian elements versus expression of each gene (Figure 5I iii, v). Two terms, ∂*f*_GATA1_/∂*x*_SPI1_ and ∂*f*_SPI1_/∂*x*_SPI1_, exhibited peaked distributions corresponding to sigmoidal-shaped response functions (Figure S6F–I), reminiscent of what is shown in Figure 1B. The other two terms ∂*f*_SPI1_/∂*x*_GATA1_ and ∂*f*_GATA1_/∂*x*_GATA1_, assume large (absolute) values even at low levels of *GATA1*, reflecting the absence of a threshold for *GATA1* inhibition/self-activation (Figure S6G–I). Interestingly, cell transfection with reporter constructs confirmed that even low levels of GATA1 can activate the *GATA1* promoter and inhibit SPI1 transactivation activities (Nerlov et al., 2000; Zhang et al., 1999). Therefore, these data-reconstructed effective response functions emphasize the complexity of dynamic gene regulation under an unperturbed intracellular environment and highlight the limitations (such as the assumed symmetry and cooperativity) of the standard equilibrium-binding model routinely used in mathematical modeling of network dynamics. Functionally, in the context of HSPC differentiation, where *GATA1* has an overall lower initial expression in HSPCs than *SPI1* (Figure 5I iv, SI6F), the *GATA1-SPI1* asymmetry may contribute to balanced lineage development. Given the high levels of *SPI1* in HSPCs and the fact that knockdown of *SPI1* to 20% of its original expression still allows emergence of GMP lineages (Rosenbauer et al., 2004), the low threshold of *GATA1* for self-activation and inhibition to SPI1 helps it to compete with *SPI1* to generate the MEP lineage. We similarly show the potential of trajectory-wise (Figure S6J) and plane-wise (Figure S6K) analyses to provide additional insights for the SPI1–GATA1 motif.

### Least action paths predict drivers of optimal hematopoietic cell-fate conversion

**Figure 6:**
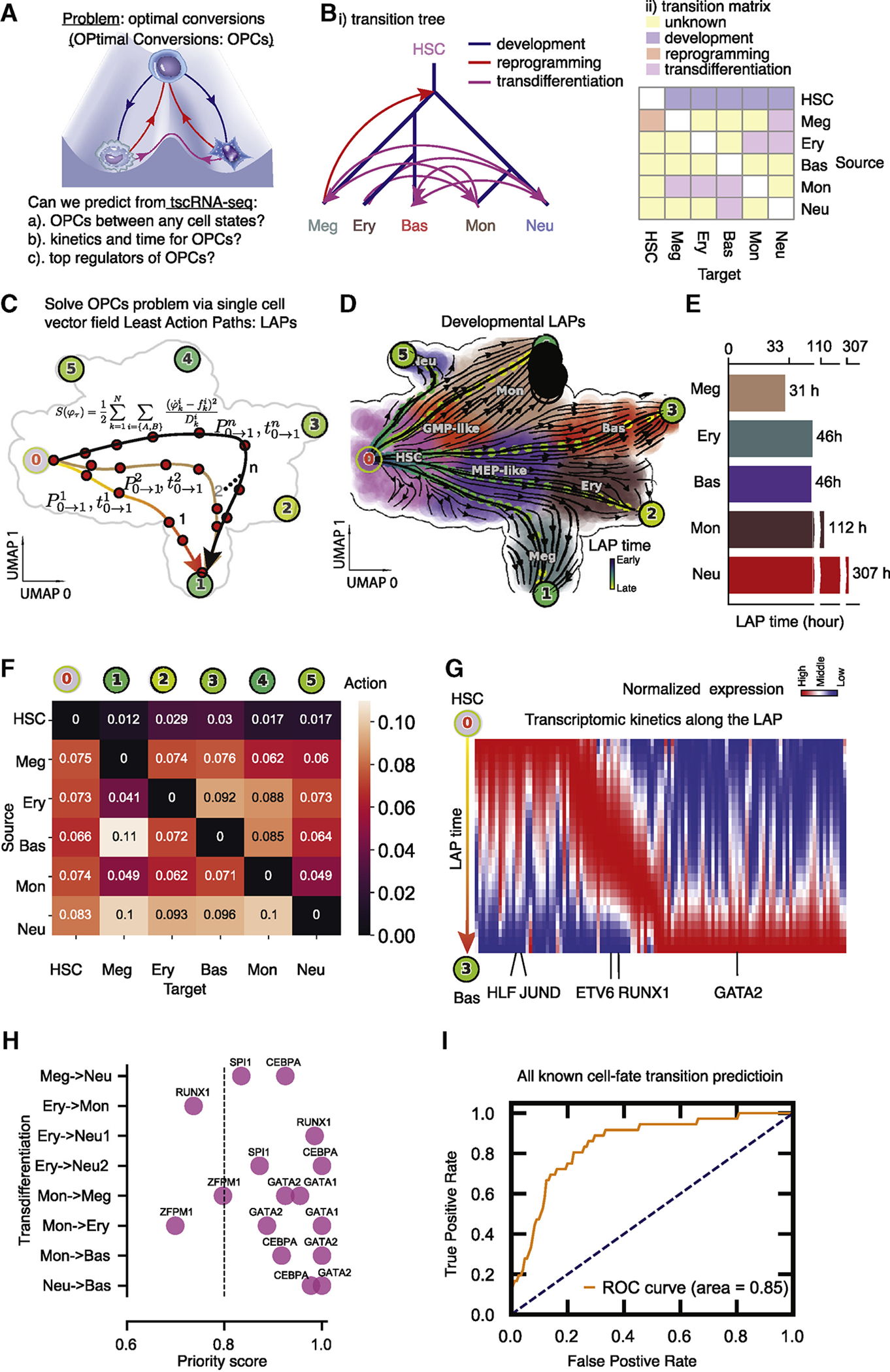
Least action path approach accurately predicts optimal cellular conversion paths. A. The grand problem of predicting OPtimal cell-fate Conversions (OPCs). B. Predicting OPCs for hematopoietic cell types. i) The developmental tree, known dedifferentiation and transdifferentiation events previously reported for the six cell types observed in our data. ii) Matrix representation of subpanel i. iii). The optimal paths for hematopoietic transitions can be found by identifying the LAPs between the fixed points that correspond to each stable cell type (STAR Methods). C. Predicted optimal developmental path (a.k.a. developmental LAP) from HSC to each of the terminal cell types in the UMAP embedding. Color of the node along the paths indicates the LAP transition time. D. The transition time of HSC to Meg lineage LAP (31 hour) is the shortest among all developmental LAPs. E. Action (STAR Methods) of the LAPs of transitions between any two hematopoietic cell states. F. Three TF-activation waves along the LAP from HSC to Bas lineage. G. Majority of TFs involved in known hematopoietic transdifferentiation are accurately prioritized by LAP predictions (STAR Methods). H. Receiver operating curve analyses of LAP TF priority predictions when using all known genes of all known transitions as the gold standard (STAR Methods). AUC: area under curve.

The ability to drive conversion between different cell states has garnered a great deal of attention as a promising avenue for disease modeling (Graf and Enver, 2009). A fundamental challenge in the field of stem cell biology is thus to assess the feasibility and identify optimal paths and key TFs (transcription factors) of such interconversions (Figure 6A) (Graf and Enver, 2009; Zhou and Huang, 2011). Recently, statistically inspired and heuristic methods (Cahan et al., 2014; Morris et al., 2014; Rackham et al., 2016) were proposed based on analyzing bulk measurements of mature cell states, and partially validated experimentally.

With the continuous vector field function built from scRNA-seq datasets available, we aimed to develop a principled strategy that reveals optimal paths, associated driving TFs, and the corresponding expression dynamics along them (Figure 6A). The hematopoietic scNT-seq dataset is well suited for testing such a method with many known developmental, dedifferentiation and transdifferentiation events (Figure 6B, Table S2).

The least action path (LAP, action: a functional of the trajectory) is a principled method (STAR Methods) that has previously been used in theoretical efforts to predict the most probable path a cell will follow during fate transition (Qiu et al., 2012; Wang et al., 2011, 2014). We reasoned that it would be possible to leverage the LAP method to make principled predictions of the optimal hematopoietic cellular conversions with the analytical vector field function (Box 3). Specifically, the optimal path between any two cell states (e.g. the fixed point of HSCs and that of megakaryocytes) is searched by variating the continuous path connecting the source state to the target while minimizing its action and updating the associated transition time. The resultant least action path has the highest transition probability and is associated with a particular transition time (Figure 6C). In order to identify the associated key regulators, we focus only on TFs and rank them by the path integral of the mean square displacement (MSD) of gene expression with respect to the initial expression.

The ability of the LAP method to estimate the dominant transition path and associated gene expression dynamics broadly yields non-trivial predictions regarding these transitions (Figure 6D–I). As an example, we analyzed transitions between the fixed points of each stable cell type in the hematopoiesis dataset. For a given differentiation process, many paths closely following streamlines of the vector field will have similar near-zero action; therefore, we characterized each transition process by the fastest LAP (F-LAP) (see STAR Methods for details). The developmental F-LAPs from HSC to terminal cell types are not simply the shortest paths in the gene expression space as would be predicted purely based on expression similarity, but follow the curved flows specified by the vector field function that incorporates expression kinetic information (Figure 6D). Furthermore, the developmental F-LAPs are distinct from and generally have shorter transition times and smaller actions than the dedifferentiation LAP paths (Figure 6F, S7A–D). Similarly, the transdifferentiation LAP from one cell type to another is distinct from that of the reverse transition, reflecting the fact that a cell is an irreversible system (Figure S7B). Notably, we found that the developmental F-LAP for the differentiation of HSC to Meg lineage requires the least time [roughly 31 hours, in line with what reported in (Yamamoto et al., 2013)], further corroborating the observation of the early appearance of Meg lineage (Figure 6E, Figure S7D).

Once the LAP is calculated in the PCA space, we can project it back to the original gene expression space to predict the full transcriptomic kinetics along the path. Exploring the dynamics of TFs along the LAP from HSC to the Bas lineage revealed three distinct waves of TFs activation (Figure 6G, see the reverse LAP at Figure S7C). We next broadly explored the ability of the LAP method to prioritize key drivers of a diverse range of hematopoietic cell fate transitions (Figure S7E, F, Fig 6H, I). We compiled known TFs for all reported normal development and reprogramming experiments and scored them based on their cumulative MSD. Experimentally identified TFs of all reported transdifferentiation events ranked consistently high in our LAP analyses (mostly in the top 80%) (Figure 6H, Figure S7F), with a total AUC (area under curve) score for all reported transitions of about 0.85 (Figure 6I).

These analyses reveal the potential of the LAP approach to predict the optimal path and TF cocktails of cell fate transitions with high accuracy, paving the road for à la carte reprogramming between any cell types of interest for applications in regenerative medicine (Graf and Enver, 2009).

### *in silico* perturbation predicts cell fate diversions after genetic perturbations

**Figure 7:**
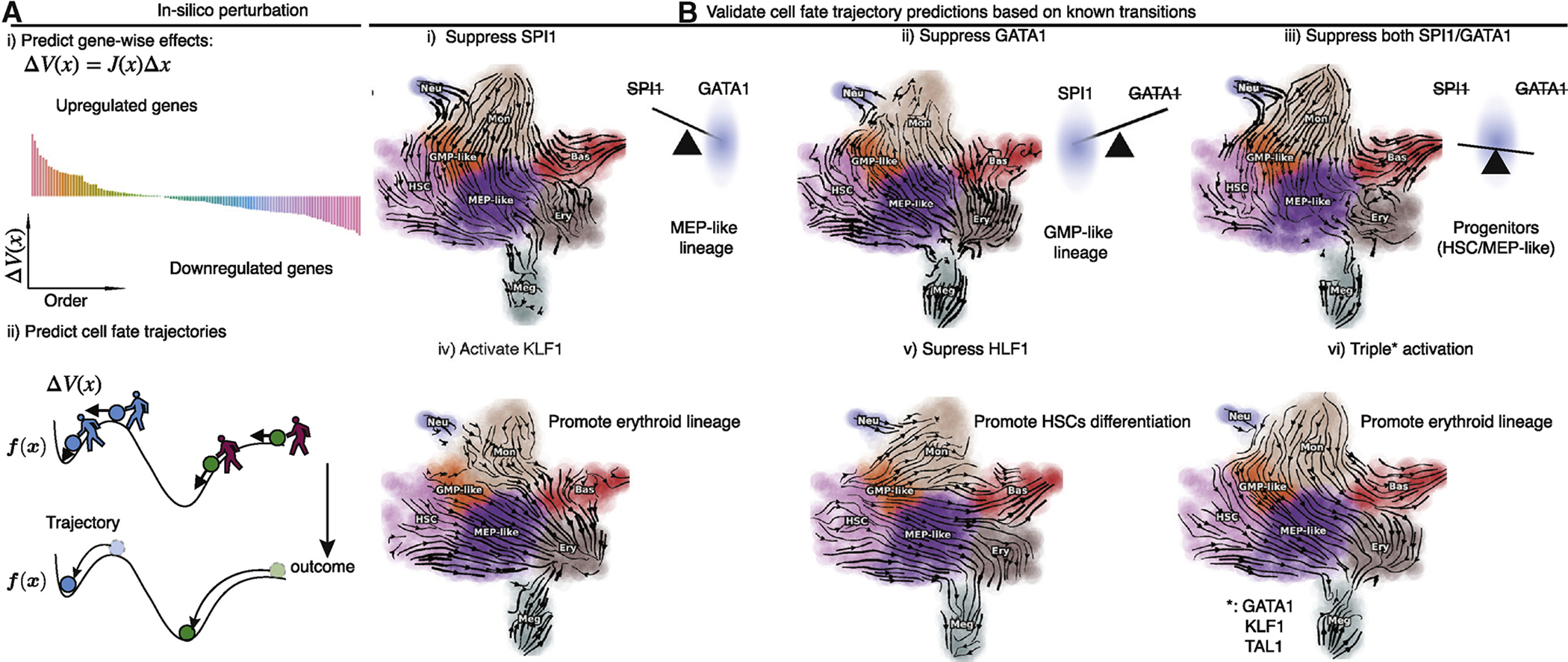
*in silico* perturbation dissects cell fate transitions under genetic perturbation A. *In silico* genetic perturbation of the velocity vector field. i) *In silico* perturbation can predict the gene-wise response. ii) *In silico* perturbation can predict the cell fate trajectory after genetic perturbation by integrating the displacement of velocities across cells. B. Validation of *in silico* trajectory predictions. i) Suppression of *SPI1* diverts cells from MEP-related lineages to GMP-related lineages. ii) Suppression of *GATA1* diverts cells from GMP-related lineages to MEP-related lineages. iii) Suppression of both *SPI1* and *GATA1* traps cells in the progenitor states. iv) Activation of *KLF1* diverts cells into the Ery lineage. v) Suppression of *HLF1* leads to differentiation of HSCs. vi) Triple activation of *GATA1*, *KLF1,* and *TAL1* leads to transdifferentiation of other lineages into erythrocytes.

The analytical form of a vector field permits *in silico* perturbation predictions of expression response for each gene in each cell (Figure 7A i) and the cell fate diversions after genetic perturbations (Figure 7A ii, STAR Methods). In particular, we demonstrated the predictive power of hematopoietic fate trajectory predictions after genetic perturbations. Interestingly, suppressing the master regulator of the GMP lineage, *SPI1*, diverts cells to megakaryocytes and erythrocytes, whereas suppressing the master regulator of the MEP lineage, *GATA1*, diverts cells to monocytes and neutrophils (Figure 7B i, ii). Suppressing both genes, however, traps the cell in the progenitor state. These predictions align well with those reported in (Rekhtman et al., 1999) and reveal a seesaw-effect regulation between *SPI1* and *GATA1* in driving the GMP and the MEP lineages (Figure 7B iii). *In silico* perturbation also correctly predicts other cellular transitions, for example, activating *KLF1* leads to conversion into erythroid cells, consistent with (Orkin and Zon, 2008) (Figure 7B iv). Similarly, suppressing the HSPC maintenance gene *HLF1* triggers cells to move away from the progenitors (Lehnertz et al., 2021) (Figure 7B v). Finally, triple activation of *GATA1*, *KLF1,* and *TAL1*, known erythrocyte factors, and TFs used for reprogramming fibroblasts into erythrocytes, diverts most other cells into the Ery lineage (Capellera-Garcia et al., 2016) (Figure 7B vi).

## DISCUSSION

A central goal of metazoan biology is to understand how a single zygote gives rise to a complex organism in a precisely coordinated fashion. Experimental advances in single cell genomics have provided a uniquely rich view of this process but we lack an appropriate analytical framework to exploit these data. In this study we developed ***dynamo*** to fulfill this unmet gap by integrating black box machine learning methods with interpretable dynamical systems approaches to gain quantitative insights from single cell datasets.

Our analytical framework consists of four integral stages. First, we estimate genome-wide kinetic rate constants and RNA velocity vectors from single-cell data. Next, we use RNA abundance and velocity vectors to reconstruct the vector field functions. We then apply differential geometry analyses made possible by the analytical vector field function, thereby obtaining biological insights. Finally, we apply the LAP method and *in silico* perturbation to predict the optimal paths of cellular state transitions and outcomes of genetic perturbations. In the first stage of kinetic parameter estimation, because our approach implements a universal modeling system, it is broadly compatible with existing RNA metabolic labeling strategies, as well as new labeling protocols that may be developed, such as dual labeling with 4sU and 6-thioguanine (6-TG) (Kiefer et al., 2018) to directly measure RNA acceleration. Furthermore, we collected a high-quality tscRNA-seq dataset for the human hematopoiesis and establish that the total RNA velocity estimated from this and other tscRNA-seq datasets with ***dynamo*** overcomes intrinsic limitations of conventional RNA velocity estimation, which can lead to inaccurate velocity measurements (Barile et al., 2021; Bergen et al., 2021), thereby enabling more accurate downstream absolute vector field analyses.

In the second stage, we take single-cell velocity vector samples as input to robustly learn a continuous vector field function in transcriptomic space. Early efforts in pseudotime ordering, RNA velocity, and sci-fate (Cao et al., 2020b), constitute important developments in dynamics inference. The key advance here is that we are now able to reconstruct analytical and continuous vector field functions in transcriptomic space. With the reconstructed continuous vector field function, we can predict the cell states over an extended time period in the past or future, as evidenced by our analysis of sequential transcriptomic profiling and clone fate tracing for neutrophil differentiation or murine hematopoiesis. Our method is also capable of *in silico* tracing the transcriptomic dynamics of cell ensembles over time (Supplementary Animation), which may provide an important complement to live-cell imaging (Baker, 2010; Wang et al., 2020) or lineage tracing (Chan et al., 2019; Frieda et al., 2017; McKenna et al., 2016).

In the third stage, we apply predictive dynamical systems methods and differential geometry analyses that extract regulatory information from the vector field function. ***Dynamo*** thus makes it possible to use single cell genomics data to directly explore governing regulatory mechanisms and even recover kinetic parameters, such as Hill coefficients, underlying cell fate transitions.

In the fourth stage, we propose two principled approaches, LAPs and *in silico* perturbation, to predict the optimal transition paths and the outcomes of genetic perturbations, respectively. The ability to perform *in silico* perturbations should facilitate the search through the vast number of possible pairwise and higher-order perturbations to discover gene combinations that lead to interesting cell states and transitions.

In summary, we have built a general framework for the analysis of transcriptional dynamics that can be applied to numerous biological systems. More broadly, when coupled with remarkable experimental advances in single cell approaches including RNA metabolic labeling (Holler et al., 2021), lineage tracing (Chan et al., 2019; McKenna et al., 2016), RNA age (Rodriques et al., 2020), signal pathway recording (Sheth and Wang, 2018), as well as genetic perturbations (Adamson et al., 2016; Dixit et al., 2016), ***dynamo*** will enable us to move towards holistic kinetic models and theories of the entire organism for cell atlas projects (Cao et al., 2020a), to understand how complex cell states arise from the combinatorial regulations of a limited number of factors, and finally to tackle the ultimate goal of converting between any cell types.

### Limitations of the Study

First, the kinetic parameter estimation for labeling experiments in ***dynamo*** still largely requires the steady-state assumption, an interesting future direction would be to develop algorithms for tscRNA-seq datasets to consider all cells instead of only extreme cells along the lines of the “dynamic model” approach from (Bergen et al., 2020), but *without* assuming a constant transcription rate, to further improve estimates for absolute kinetic parameters and velocity. Second, our vector field learning approach currently focuses on deterministic aspects of dynamics but should extend to the stochastic aspects of the model as well. Third, the reconstructed vector field functions can be confounded by unobserved hidden variables. Incorporating datasets from the recent developments of single cell multi-omics (Cao, 2020; Ma et al., 2020), spatial transcriptomics (Chen et al., 2021; Moffitt et al., 2018; Rodriques et al., 2019), or both (Liu et al., 2020) into our framework will provide the opportunity to address the hidden variable problem.

## STAR+METHODS

### RESOURCE AVAILABILITY

#### Lead contact

Further information and requests for resources and reagents should be directed to and will be fulfilled by the Lead Contact, Jonathan Weissman (weissman@wi.mit.edu).

#### Materials availability

This study did not generate new unique reagents.

#### Data and code availability

The following public cscRNA-seq datasets are used in this study: the hematopoiesis clone tracing dataset (Weinreb et al., 2020). The following public tscRNA-seq datasets are used in this study: scSLAM-seq (Erhard et al., 2019), scNT-seq (Qiu et al., 2020a), sci-fate (Cao et al., 2020b), and scEU-seq (Battich et al., 2020). All datasets can be directly downloaded with ***dynamo***. The raw and processed data for the 10x scRNA-seq, the scSLAM-seq clone tracing experiment and the human hematopoiesis scNT-seq will be accessible via GEO upon publication of this study.

***Dynamo*** (version: 1.0) is implemented as a Python package and is available through GitHub (https://github.com/aristoteleo/dynamo-release). Notebooks, tutorials for reproducing all figures in this study, and tutorials of ***dynamo*** usage cases are also available through GitHub (https://github.com/aristoteleo/dynamo-notebooks, https://github.com/aristoteleo/dynamo-tutorials).

### EXPERIMENTAL MODEL AND SUBJECT DETAILS

#### Cell Culture of HL60 cells and primary human CD34+ hematopoietic stem and progenitor cells

HL60 cells (ATCC® CCL-240™) were grown in RPMI 1640 medium (Gibco), with 20% FBS + 5% Penicillin-Streptomycin) at 37°C under 5% CO2, supplemented with 10% fetal bovine serum (Sigma) and 1% Penicillin/Streptomycin (HyClone). Cells were maintained below a density of 10^6^ cell/mL. On the first day of the differentiation experiment, cells were seeded at 200,000 cell/mL in 12 well plates (unless stated otherwise) and treated with 1 μM ATRA (all-trans-retinoic acid, Cat#R2625-100MG) to differentiate into either the neutrophil-like cells. Cell differentiation status was confirmed by flow cytometry analysis of *CD14* (Biolegend, Cat#367117) and *CD11b* (Biolegend, Cat#301309).

For the human hematopoiesis dataset, we cultured primary human CD34+ hematopoietic stem and progenitor cells obtained from the Fred Hutchinson Cancer Research Center. Cells were thawed and cultured in StemSpan SFEM II human hematopoietic stem cell expansion media (StemCell Technologies, Cat#02690) supplemented with StemSpan CC100 (StemCell Technologies, Cat#02690) and 50 ng/ml TPO (PeproTech, Cat#300-18-100UG). Cells were allowed to differentiate over the course of 1 week.

### METHOD DETAILS

#### Profiling HL60 cell differentiation with 10x Chromium–based scRNA-seq

HL60 differentiations were initialized on different days so that all samples could be harvested in a single scRNA-seq reaction to minimize batch effects. Cells were treated with 1 μM ATRA and differentiated for 0 (no ATRA treatment), 1, 2, 3, 4, or 5 days, with all differentiations performed in biological replicates. Samples were tagged or “cell hashed” (Stoeckius et al., 2018) with distinct BD sample tags (BD Bioscience, cat#PN 633780) to enable demultiplexing of cells, and then pooled for scRNA-seq. scRNA-seq was performed on one lane of the 10x Chromium™ Single Cell 3’ v2 system following the standard library prep protocol (10x Genomics Single Cell 3’ Reagent Kits v2 User Guide, CG00052). Libraries were amplified with 10 cycles of cDNA amplification and 15 cycles of Sample Index PCR. BD Sample Tags were size-separated by SPRI selection after cDNA amplification and amplified according to standard protocols (BD User-Demonstrated Protocol: BD Single-Cell Multiplexing Kit—Human Doc ID: 179682 Rev. 1.0). Final cDNA and sample tag libraries were sequenced on a NovaSeq 6000 (Illumina).

#### scSLAM-seq

Our scSLAM-seq protocol was adapted from (Erhard et al., 2019; Hendriks et al., 2019). Before proceeding with the protocol using cells collected on particular days (see below), HL60 cells were labeled in medium with 100 mM 4sU (Lexogen) for about 60 minutes at 37°C and sorted into lysis buffer (4μL, 0.5 U/μL Recombinant RNase Inhibitor (Takara Bio, 2313B), 0.0625% Triton X-100 (Sigma, 93443–100ML) in 96-well PCR plates. All plates were frozen at −80°C until use. After thawing the plates to room temperature, to the lysed cells, 0.4*μ*L of 10x PBS and 4.4*μ*L of alkylation mix (20 mM IAA in 100% DMSO) was added for a final concentration of 10 mM IAA, 50% DMSO. Alkylation was stopped by addition of 1.3 μL of 100 mM DTT and incubating for 5 minutes at room temperature. Alkylated RNA was purified with 1.1 volume of Ampure XP beads and two washes with fresh 80% ethanol, and eluted into an RNA elution buffer (4μL, 3.125 mM dNTP mix (Thermo Fisher, R0193), 3.125 μΜ Oligo-dT30VN (Integrated DNA Technologies, 5′AAGCAGTGGTATCAACGCAGAGTACT30VN-3′), 0.5 U/μL Recombinant RNase Inhibitor, 1:24million ERCC RNA spike-in mix (Thermo Fisher, 4456740)). cDNA and the remaining library preparation was performed according to a modified version of the protocol for Smart-seq2 (Tabula Muris Consortium, 2020). The prepared libraries were sequenced on MiSeq and NovaSeq5000 platform (Illumina), generating paired-end reads with 100 PCR-cycle.

#### Sequential lineage tracing of HL60 cell differentiation with static barcode and scSLAM-seq

To facilitate lineage tracing in scSLAM-seq libraries, cellular barcodes (GBCs) were introduced using a lentiviral transduction strategy (Adamson et al., 2016). Given that the success of this experiment critically depended on the uniqueness of barcode sequence to each cell at the start of the experiment, i.e. low barcode collision rate, and the capture of clone cells (clones with the same barcodes) across different days, we used an experimental scheme in which the starting population of the HL60 cells were infected at a low (2%) multiplicity of infection (MOI). This scheme has two benefits: first, we obtained a small number of barcoded single cells (~2000 in 1 ml of media in each well of a 24-well plate) so that we could capture clone cells via plate-based SLAM-seq (scRNA-seq augmented by metabolic labeling) characterized of low throughput; second, co-culturing the small number of infected cells with a large population of uninfected cells enabled us to differentiate infected cells more conveniently, as a small number of cells are difficult to grow and differentiate. Single cells carrying barcodes and expressing the blue fluorescent protein (BFP) reporter were sorted (Sony SH800) at five timepoints, days 0, 1, 2, 3, and 5, during differentiation in the presence of ATRA. cDNA from single cells was prepared in a 96-well format as previously described (Tabula Muris Consortium, 2020) following alkylation and RNA cleanup (Erhard et al., 2019; Hendriks et al., 2019). Sequencing libraries were either reformatted into a 384-well format and prepared using TTP Mosquito automated liquid handlers, or in a 96- well format using a multichannel pipette. GBC sequencing libraries were prepared by dual PCR amplification to enrich for GBC cDNA and to add Illumina adapters and dual indexes complimentary to that cell’s transcriptome sequencing library indexes. GBC sequencing libraries were spiked into transcriptome libraries at 1:10 and sequenced on the NextSeq or MiSeq platform (Illumina). Transcriptome libraries were sequenced separately using a NovaSeq5000 S2 300-cycle kit.

#### Profiling human hematopoiesis *in vitro* with scNT-seq

Our scNT-seq protocol was adapted from (Qiu et al., 2020a). For the one-shot metabolic labeling experiment in primary human CD34+ hematopoietic stem and progenitor cells, 4-thiouridine (4sU) (Sigma, T4509–25MG) dissolved in DMSO was added into human hematopoietic stem cell expansion media at a final concentration of 400μM. Cells were labeled for 5 hours for the day 4 sample and 3 hours for the day 7 sample. Exposure of the samples to light was minimized throughout the experiment to avoid 4sU degradation.

Upon completion of the labeling phase, cells were washed twice with 0.01% bovine serum albumin (BSA, Sigma-Aldrich, A8806–5G) in Dulbecco’s Phosphate-Buffered Saline (DPBS) and filtered through a 40 μm strainer (Corning, 431750). Subsequently, the samples were diluted to a concentration of 120 cells/μL in DPBS-0.01% BSA, and loaded into a 10-mL Luer lock syringe (BD, 300912) containing a magnet (V&P Scientific, 782N-6-150), and stirred gently. 10mL of lysis buffer containing 4 mL of water, 3 mL of 20% Ficoll PM 400 (Sigma, 26873-85-8), 100 μL of N-lauroylsarcosine sodium salt solution 20% (Sigma, 137-16-6), 400μL of 0.5M EDTA (Invitrogen, 15575-038), 2 mL of 1M Tris-HCl, pH 7.5, and 500 μL of 1 M DTT (Caiman Chemical, 700416) was used to resuspend barcoded oligo-dT primer on beads for Drop-seq [ChemGens, MACOSKO-2011-10(V+)] at a concentration of 130 beads/μL in a 10-mL Luer lock syringe containing a magnet. Droplet generation oil (Biorad, 1863005) was dispensed using a 30-mL Luer lock syringe (BD, 302832). Cells (4,000 μL/h), oil (15,000 μL/h) and beads (4,000 μL/h) were transferred into uFluidix Drop-seq chips with hydrophobic coating using KD Scientific Syringe Pumps (KDS, 78-2910) and micromedical tubing (Scientific Commodities, BB31695-PE/2), and visualized using a Photron Fastcam SA5 camera. Droplets were collected in 50-mL conicals.

Droplet breakage was performed by removing oil and adding 30 mL of 6X SSC (diluted from Life Technologies, 15557044) and 1 mL of 1H,1H,2H,2H-perfluoro-1-octanol 97% (Sigma-Aldrich, 370533), followed by vigorous shaking. The supernatant was removed following a 1-minute centrifugation at 1,000 g, and 20 mL of 6X SSC was added twice to resuspend the beads, which were then transferred to new tubes and centrifuged at 1,000 g for 2 minutes. Beads were resuspended in 6X SSC, transferred to 2-mL Lobind tubes, and washed twice with 1 mL of 6X SSC. This and all subsequent washing steps were performed by centrifuging the samples in a spinning bucket centrifuge at 1,000 g for 1 minute.

4sU was converted to cytosine analogs using TimeLapse-seq chemistry, which gives rise to apparent U-to-C mutations following reverse transcription (Schofield et al., 2018). Briefly, beads were washed using a mix of 16 μL of 3M sodium acetate pH 5.2 (Thermo Scientific, R1181), 4 μL of 0.5 M EDTA pH 8.0, and 430 μL of water. Next, beads were resuspended in a mixture of 8 μL of 3M sodium acetate pH 5.2, 2 μL of 0.5 M EDTA pH 8.0, 13 μL of 2,2,2-trifluoroethylamine (Sigma-Aldrich, 91692-5ML), 13 μL of freshly made 192 mM sodium periodate (Sigma-Aldrich, 311448-5G), and 214 μL of water and incubated at 45°C for 1 hour with rotation. Beads were then washed once with 1 mL of TE (Sigma-Aldrich, 93302-100ML) and incubated for 30 minutes at 37°C in a mixture of 5 μL of 1 M Tris-HCl pH 7.5, 5 μL of 1 M DTT, 10 μL of 5M NaCl, 1 μL of 0.5M EDTA, 10 μL of RiboLock RNase inhibitor (Thermo Scientific, EO0381), and 469 μL of water. A subsequent wash was performed with 1 mL of Tris-HCl buffer (10 mM, pH 8.0) and 0.3 mL of Maxima H Minus 5X RT buffer (Thermo Scientific, EP0751). Reverse transcription was performed by incubating beads with a mixture of 40 μL of Maxima H Minus 5X RT buffer, 40 μL of 20% Ficoll PM-400, 20 μL of 10mM dNTPs (NEB, N0447L), 5 μL of 100μM template switch oligo (AAGCAGTGGTATCAACGCAGAGTGAATrGrGrG), 5 μL of RiboLock RNase inhibitor, 10 μL of Maxima H Minus reverse transcriptase enzyme (Thermo Scientific, EP0751) and 80 μL of water for 30 minutes at room temperature and 120 minutes at 42°C, with rotation. Finally, beads were washed with 1 mL of TE-SDS (10 mM Tris pH 8.0, 1 mM EDTA, and 0.5% SDS), and twice with 1 mL of TE-TW (10 mM Tris pH 8.0, 1 mM EDTA, and 0.01% Tween-20).

Beads were then washed once with 1mL 10mM Tris-HCl pH 8.0, and treated with a mix of 10μL of exonuclease I (NEB, M0293L), 20μL 10X exonuclease I buffer (NEB, M0293L) and 170μL of water at 37°C for 45 minutes, and washed with 1 mL of TE-SDS and twice with 1 mL of TE-TW. To prepare beads for second strand synthesis, beads were resuspended in 500μL fresh 0.1N NaOH, incubated at room temperature with rotation for 5 minutes, and neutralized using 500μL 0.2M Tris-HCl (pH 7.5). A wash with TE-TW and one with 10mM Tris-HCl (pH 8) were performed. Subsequently, the beads were resuspended in a mix of 20μL 10X Blue buffer (Enzymatics, P7010-HC-L), 40μL 20% Ficoll PM-400, 20μL 10mM dNTPs, 10μL 100μM TSO-N9 primer (/5SpC3/AAGCAGTGGTATCAACGCAGAGTGAAT(N1:25252525)(N1)(N1)(N1)(N1)(N1)(N1)(N1)(N1)), 5μL 100μM TSO-GAATG primer (/5SpC3/AAGCAGTGGTATCAACGCAGAGTGAATG), 10μL Klenow exo- (Enzymatics, P7010-HC-L) and 95μL of water, and incubated at room temperature for 10 minutes and at 37°C for 60 minutes with rotation. Finally, beads were washed with 1 mL of TE-SDS and twice with 1 mL of TE-TW.

We next determined the optimal number of cycles required for whole-transcriptome amplification by performing qPCR on an aliquot of 6,000 beads. Following two washes in water, beads were resuspended in a mixture of 25 μL KAPA HiFi HS ReadyMix (Roche, 07958935001), 0.4 μL of 100 μM TSO-PCR primer (AAGCAGTGGTATCAACGCAGAGT), and 24.6 μL of water. A first PCR was run using the following parameters: 95°C for 3 minutes; 4 cycles of (98°C for 20 seconds, 65°C for 45 seconds, and 72°C for 3 minutes); 9 cycles of (98°C for 20 seconds, 67°C for 20 seconds, and 72°C for 3 minutes); 72°C for 5 minutes; and hold at 4°C. The PCR product was purified using one round of 0.7X AMPURE XP beads (Beckman Coulter, A63881). One μL of purified cDNA was added to 4.5 μL of KAPA HiFi HS ReadyMix spiked with SYBR Green Dye (Lonza, 12001-796), 0.07 μL of 25 μM TSO-PCR primer and 3.53 μL of water. qPCR was run using the following parameters: 95°C for 3 minutes; 25 cycles of 95°C for 15 seconds, 63°C for 30 seconds, and 72°C for 30 seconds). The extra number of cycles to add to the last stage of the first PCR was three-fourths of the cycle number coinciding with the exponential amplification stage of the qPCR.

Large-scale PCR amplification was performed on the rest of beads with the same parameters as the first PCR above, plus the additional number of cycles in the last stage determined by qPCR. To ensure high diversity in our libraries, multiple tagmentations were performed for the cDNA from each of the timepoints using the Nextera XT DNA Library Prep Kit (Illumina, FC-131-1096), and amplified in a second round of PCR (15 μL of Nextera PCR mix, 5 μL of 2 μM P5-TSO hybrid primer – AATGATACGGCGACCACCGAGATCTACACGCCTGTCCGCGGAAGCAGTGGTATCAA CGCAGAGT*A*C–, and 5 μL of 2 μM Nextera N70X oligo from (Buenrostro et al., 2015) using the following parameters: 95°C for 30 seconds; 12 cycles of (95°C for 10 seconds, 55°C for 30 seconds, and 72°C for 30 seconds); 72°C for 5 min; and hold at 4°C. PCR products were purified using two rounds of 0.6X AMPURE XP beads, and the fragment size was measured using the Agilent 2100 Bioanalyzer High Sensitivity DNA kit (Agilent Technologies, 5067-4626 and 5067-4627). Pooled libraries were quantified using the KAPA Library Quantification Kit (07960204001), and sequenced in an Illumina NovaSeq 6000 System using a S1 flow cell with a 20 base pair (Read 1), 75 base pair (Read 2), and 8 base pair (Index 1) configuration and an HPLC-purified custom read 1 primer (GCCTGTCCGCGGAAGCAGTGGTATCAACGCAGAGTAC).

#### Binomial mixture model to quantify labeled and unlabeled RNA

We use the binomial mixture model first described in the GRAM-SLAM study (Jürges et al., 2018) to estimate the fraction of labeled (*π*_*g*_) and unlabeled reads of each gene *g* for the scSLAM-seq data that we produced. The probability of *y* T-to-C mutations in a read that contains *n* possible mutation sites can be defined with the following equation:

$$P\left(y;p_{\text{e}},p_{\text{c}},n,\pi_{\text{g}}\right)=\left(1-\pi_{\text{g}}\right)B\left(y,n,p_{\text{e}}\right)+\pi_{\text{g}}B\left(y,n,p_{\text{c}}\right),$$

where *p*_*e*_ is the background T-to-C mutation rate that is independent of the mutations introduced by metabolic labeling, and *p*_*c*_ is the T-to-C mutation rate introduced by metabolic labeling. *B*(*y*, *n*, *p*) is the binomial probability mass function. Estimation of *p*_*c*_, *p*_*e*_, and *π*_*g*_ is performed using the pipeline from (Hendriks et al., 2019) with a few custom adaptations for the clone tracing scSLAM-seq dataset. We define the ratio between the true (*π*^True^) and estimated (*π*_*g*_) fraction of labeled reads as the labeling correction coefficient, denoted as $\rho =\pi_{g}/\pi^{True}$. When the fraction of labeled RNA is overestimated, *ρ* is larger than 1 and vice versa.

#### Quantifying splicing and labeling data of the human hematopoiesis scNT-seq experiment with *dynast*

We developed ***dynast*** (https://github.com/aristoteleo/dynast-release), an inclusive and efficient command-line toolkit for preprocessing data from metabolic labeling–based scRNA-seq experiments and quantifying the following four types of mRNA species (relies on the binomial mixture model): unspliced, spliced, labeled (or new), and total RNAs (details will be reported elsewhere). Samples from days 4 and 7 were each subjected to standard ***dynast*** runs, using human genome hg38 as the alignment reference. The resultant objects were first filtered to keep only high quality cells and then merged to obtain in total about 2,000 high-quality cells. This merged object was then used for all downstream analyses.

#### Effects of under and overestimation of labeled RNA fraction on tscRNA-seq kinetic parameter estimation and velocity calculation

For one-shot experiments, the slope of the linear relationship between labeled RNA *l* and total RNA *r* is proportional to the labeling correction coefficient *ρ*:

$$k=\rho \left(1-e^{-\gamma t}\right).$$

Therefore, an overestimated *ρ* amounts to a high NTR (new to total RNA ratio) at steady state. For one-shot experiment, we assume that the labeling data have been statistically well corrected with the mixture binomial model, and thus the *ρ* is effectively close to 1. Then the slope is approximately *k* = 1 − *e*^−*γt*^, allowing us to obtain the degradation rate constant *γ* from the NTR slope. We can evaluate the error between the estimated *γ* under this assumption, and the true degradation rate constant *γ*^*true*^:

$$\gamma -\gamma^{\text{true}}=-\frac{1}{t}\left(\ln\left(1-\frac{k}{\rho }\right)-\ln(1-k)\right)\;\;=-\frac{1}{t}\ln\left(\frac{1-\frac{k}{\rho }}{1-k}\right)\;.$$

When *ρ* < 1, *γ* < *γ*^*true*^, *γ* is underestimated. Consequently, the velocity of total RNA, considering only its magnitude, differs from the true velocity by,

$$|\dot{r}|-\left|{\dot{r}}^{\text{true}}\right|=\left|\frac{\gamma n}{k}-\gamma r\right|-\left|\frac{\gamma^{\text{true}}n}{k}-\gamma^{\text{true}}r\right|\;\;=\left(\gamma -\gamma^{\text{true}}\right)\left|\frac{n}{k}-r\right|\;.$$

Note that *n*/*k* − *r* determines the sign of the velocity, i.e., *n*/*k* − *r* > 0 amounts to a positive velocity, and vice versa. Therefore, under-correction of labeled RNA leads to underestimation of the velocity. It is also apparent that a *ρ* higher than one leads to the overestimation of both the degradation rate constant *γ* and the velocity of total RNA. The labeling correction coefficient *ρ*, which is assumed to be constant across all time points, has minimal impacts on curve fitting methods for degradation rate constants because the time scale of a first order degradation is independent of initial concentrations. However, for kinetics experiments, *ρ* affects the curve fitting of both the synthesis of labeled RNAs, and the degradation of unlabeled RNAs. The transcription rate *α* is under-estimated when *ρ* < 1, and overestimated when *ρ* > 1. The kinetics of unlabeled RNA are not merely a degradation process when *ρ* < 1, as there are artificial increases of new RNA due to underestimation of labeled RNA, and the degradation appears slower than the true rate. By contrast, when *ρ* > 1, the degradation of the unlabeled RNA is unaffected for similar reasons as in degradation experiments. As a result, the cluster-wise velocity for kinetic experiments is underestimated when *ρ* < 1 and overestimated when *ρ* > 1. In the extreme cases, an underestimated labeling RNA fraction can lead to a sign change in the velocity. On the other hand, because the cell-wise velocity is:

$$\dot{r}=\frac{\gamma l}{1-e^{-\gamma t}}-\gamma r.$$

It is unaffected by the inaccurate estimation of *α*, and an inaccurately estimated *γ* alters its magnitude but not the sign.

#### Dynamo: from velocity vector samples to continuous vector field functions and differential geometry analysis

Our analytical framework, ***dynamo***, consists of four integral stages: 1) estimation of genome-wide kinetic rate constants and velocity vectors, 2) reconstruction of single-cell vector field functions with the resultant cell state and velocity samples, 3) in-depth analyses leveraging various differential geometry analyses, and 4) predictions of optimal paths and cell fate diversions induced by genetic perturbations.

As the core of the first stage, we develop a comprehensive parameter estimation framework that includes all key steps involved in expression dynamics. This complete model assumes that the promoter of a gene stochastically switches, with inactivation rate *a* and activation rate *b*, between an active state (*A*, with a high transcription rate *α*_*A*_) and an inactive state (*I*, with a much lower transcription rate *α*_*I*_) (Golding et al., 2005) Next, we explicitly model the accumulation or decay of 4sU-labeled RNAs (Figure 2A, B, also see below), which are subsequently captured by scRNA-seq augmented with RNA metabolic labeling. We denote the ratio between the true $\left(\pi_{g}^{\text{True}}\right)$ and estimated (*π*_*g*_) fraction of labeled reads for gene *g* as the **labeling correction coefficient**. Our model further incorporates RNA splicing dynamics with the splicing rate constant *β*. The degradation of the spliced RNA is captured by the degradation rate constant *γ*_*s*_. The protein translation rate constant *η* and degradation rate constant *γ*_*p*_ are also modeled in ***dynamo*** for possible datasets from single-cell transcriptomic–proteomic coassays. For the purpose of simplicity, this work mainly focuses on RNA transcription, splicing, degradation, and metabolic labeling. We analyze various types of scRNA-seq data with and without metabolic labeling. For the former, we consider four possible experimental scenarios (Figure 2C); for each case, one may or may not consider RNA splicing. We use three groups of models (Figure SI2A) to describe these various types of scRNA-seq data. Details on how to estimate the RNA turnover rates and RNA velocities for each case are given below.

#### Dynamo: 1) Estimation of genome-wide kinetic rate constants and velocity vectors

##### Limitations of conventional RNA velocity methods for scRNA-seq experiments without metabolic labeling

Most existing pseudotime ordering methods merely reveal the central trend of a population of cells. By contrast, RNA velocity (La Manno et al., 2018), an important recent development in inferring dynamics of single cells, explicitly models the RNA kinetics to offer a local extrapolation, for a period up to a few hours, of cell fate transitions of individual cells by exploring the intron or exon reads incidentally captured by most scRNA-seq platforms. The conventional RNA velocity method (La Manno et al., 2018) from the original paper exploits the kinetics of RNA transcription, splicing, and degradation with corresponding ODEs (ordinary differential equations) as follows:

$$\dot{u}=\alpha -\beta u,$$

$$\dot{s}=\beta u-\gamma s,$$

where *u* and *s* are the copies of unspliced and spliced RNA for a particular gene in a cell, respectively; *α*, *β*, and *γ* are the rate constants for transcription, splicing, and degradation (see ***Impacts of Dimensions of Rate Constants on RNA Velocity*** for a discussion of “rate” and “rate constant”, as well as their dimensions), respectively. In this study, we classify such a model system as **Model 1**. If we can estimate the kinetic parameters (*α*,*β*, and *γ*), together with *u* and *s* measured by scRNA-seq, we can derive a measure of “RNA velocity” of unspliced $\left(\dot{u}\right)$ or spliced RNA $\left(\dot{s}\right)$ that reveals the direction and magnitude of rate of change of gene expression of each gene in each cell. Because in general *α* is not constant, but rather a function of the cell state and other variables (e.g., abundance of transcription factors, extrinsic signals, etc., see more below), it is difficult to obtain the unspliced RNA velocity. On the other hand, splicing and degradation rate constants (*β*) can in most cases be approximated as constants for certain cell types. The question, then, is how to estimate those kinetic parameters. Assuming pseudo-steady state $\left(\dot{s}=0\right)$ for cells with extreme high unspliced and spliced RNA expressions (top right corner of the phase plane), one reaches the following linear relation between the spliced and unspliced RNA

βu=γs.

Let $\tilde{\gamma }=\gamma /\beta$, the above relation can be rewritten as:

$$u=\tilde{\gamma }s.$$

A linear regression of cells at steady states can be performed to obtain $\tilde{\gamma }$. Thus, the conventional RNA velocity as defined in the original study is given by:

$$v=u-\tilde{\gamma }s.$$

Note that *v* is equal to $\dot{s}$ up to the splicing rate constant *β*, which is in general gene-specific as revealed in Figure 2D. Because velocity can be estimated for each gene in each cell, velocities of all genes in any cell form a high-dimensional vector, with each dimension corresponding to a gene. This high-dimensional velocity vector is often projected into a low-dimensional space for visualization using either pearson or cosine kernels (Bergen et al., 2020; La Manno et al., 2018; Li et al., 2020) to reveal the direction of cell fate transitions in low-dimensional space via projected velocities.

Although conventional RNA velocity has been successfully applied to a variety of studies, it has several limitations:

1. Because the intron reads are generated through mis-priming on polyA- or polyT- enriched intronic regions of nascent pre-RNA, it can be difficult to apply conventional RNA velocity to most transcription factors, which are typically expressed at low levels, and genes with no polyA/T-enriched intron regions;
2. Although many biological systems, including hematopoiesis, involve rapid but coordinated changes of RNA transcription rates for a considerable number of genes (Barile et al., 2021), existing methods for estimating splicing RNA velocity (Bergen et al., 2020; La Manno et al., 2018) all assume constant transcription rates (*α*_on_ for the induction phase, *α*_off_ for the repression phase, Figure 3E.) and often lead to nonsensical backward RNA velocity flow;
3. The linear regression methods used by conventional RNA velocity ignores the distribution of unspliced and spliced RNA, which can be used to improve the estimation of kinetic parameters;
4. For systems far away from the pseudo-steady state, using cells with extreme RNA expression levels for linear regression may lead to inaccurate velocity calculations for most cells;
5. The time scale for the degradation rate constant in conventional RNA velocity $\left(v=u-\tilde{\gamma }s\right)$ is relative to that of the splicing rate β. This makes the estimated velocity a relative quantity.
6. Conventional RNA velocity only estimates velocities for observed cells. Thus, it is a discrete, sparse, and local measure of cell dynamics and often merely used as a descriptive rather than a predictive tool.

A great deal of efforts have been devoted to the improvement of conventional RNA velocity estimation (La Manno et al., 2018) in regard to challenges 3) and 4) and extend the concept to “protein velocity” (Gorin et al., 2020), but 1), 2), and 5) are fundamental limitations that cannot be resolved at the computational level without additional experimental information. In this section, we introduce our methods for analyzing conventional scRNA-seq data, addressing some of the issues with existing RNA velocity methods. In the next section we focus on computational methods for computing RNA velocity for metabolic labeling data, which reconciles the splicing- and labeling-based kinetics and overcomes other drawbacks of conventional RNA velocity methods. Finally, to address 6), we go beyond RNA velocity samples of single cells to map the continuous vector field functions in transcriptomic space and perform sophisticated differential geometry analyses to gain various functional vector field predictions and biological insights.

##### Generalized method of moments (stochastic splicing and negative binomial distribution method) improves RNA velocity estimation for conventional scRNA-seq experiments

Current scRNA-seq methods have low RNA capture rates that lead to frequent “dropouts,” in which individual RNA levels are not observed. In order to alleviate dropout effects and measurement noises as well as to improve the robustness of the estimation, the original RNA velocity method (La Manno et al., 2018) utilizes the mean expression (first moment) of each gene across cells, calculated based on the *k*-nearest neighbor graph of cells, instead of the raw expression:

$$\langle u\rangle =\frac{1}{k}\sum_{i\in N}u_{i},$$

$$\langle s\rangle =\frac{1}{k}\sum_{i\in N}s_{i},$$

where N (30 by default in ***dynamo***) is the set of *k*-nearest neighbors of each individual cell, often constructed in the space of the top PCs (principal components) (e.g., 30 PCs), reduced from the original gene expression space of highly variable genes. These can be considered as estimators of the first moments of the distribution of unspliced and spliced RNAs. RNA velocity calculations performed on the first moments lead to a cleaner phase plane and therefore smoother velocity vectors (La Manno et al., 2018). However, higher moments of the distribution are ignored in the original linear regression method.

Second moments (uncentered variances and covariances) provide information in addition to first moments on the shape of the underlying distribution. It is thus desirable to also take advantage of the second moments to improve the estimation robustness and accuracy of the kinetic parameters, and thus that of the RNA velocity measurements. The second moments of unspliced and spliced RNA, as well as their mixed moments, also rely on the *k*-nearest neighbor graph of cells, and can be computed as follows:

$$\langle u^{2}\rangle =\frac{1}{k}\sum_{i\in N}u_{i}^{2},$$

$$\langle s^{2}\rangle =\frac{1}{k}\sum_{i\in N}s_{i}^{2},$$

$$\langle us\rangle =\frac{1}{k}\sum_{i\in N}u_{i}s_{i},$$

with the first, second, and mixed moments of unspliced and spliced RNAs for each gene across cells, one can apply the generalized method of moments (GMM) to improve the estimation of kinetic parameters ***θ*** (e.g. *α*, *β*, and *γ*), in lieu of the linear regression on mean expressions as used in the original RNA velocity method. Instead of directly fitting the distribution, GMM seeks to solve the following equations of moments for ***θ***, also known as *moment conditions*:

$$\langle g_{1}(X,\theta )\rangle =0,\;\langle g_{2}(X,\theta )\rangle =0,\;\langle g_{3}(X,\theta )\rangle =0,\;\;\cdots$$

where *g*_1_, *g*_2_, *g*_3_, … are functions of the random variables ***X*** (e.g. the copies of spliced and unspliced RNA across cells) and parameters ***θ***. The optimal ***θ*** can be found by minimizing the Euclidean norm of the above expectations:

$$\theta^{*}=\underset{\theta }{\arg\min}{\langle g(X,\theta )\rangle }^{⊤}W\langle g(X,\theta )\rangle ,$$

where ***g*** is a vector-valued function consisting of the moment conditions, and ***W*** is a positive definite weighting matrix, defined as the inverse of the covariance matrix:

$$W={\left(\frac{1}{n}\sum_{i=1}^{n}\varepsilon \varepsilon^{⊤}\right)}^{-1},$$

where ***ε*** is the error term for the moment conditions when applied to actual data.

Specifically, to apply GMM in the context of RNA velocity, one needs to find the moment conditions for first and second moments. The unspliced and spliced RNA in **Model 1** (Figure S2A) are generated stochastically during the transcription and splicing processes, which can be mathematically described by master equations. By deriving the ODEs for first and second moments from the master equations, Berger et al. showed that the moment conditions are (Bergen et al., 2020):

$$\left[\begin{array}{c} \langle u\rangle \\ \langle u\rangle +2\langle us\rangle \end{array}\right]=\tilde{\gamma }\left[\begin{array}{c} \langle s\rangle \\ 2\langle s^{2}\rangle -\langle s\rangle \end{array}\right]+\left[\begin{array}{c} \varepsilon_{1}\\ \varepsilon_{2} \end{array}\right]\;\;\Rightarrow y=\tilde{\gamma }x+\varepsilon ,$$

where $\tilde{\gamma }=\gamma /\beta$, and *ε*_1_ and *ε*_2_ are the error terms for the two moment conditions. Given vector pairs ${\left\{x_{i},y_{i}\right\}}_{i=1}^{n}$ of the first and second moments computed from the conventional scRNA-seq data in *n* cells at pseudo-steady state, the optimal $\tilde{\gamma }$ is obtained by minimizing the following least squares:

$$\tilde{\gamma }=\underset{\tilde{\gamma }}{\text{argmin}}\sum_{i=1}^{n}{\left(y_{i}-\tilde{\gamma }x_{i}\right)}^{⊤}W\left(y_{i}-\tilde{\gamma }x_{i}\right)=\frac{\sum_{i}x_{i}^{⊤}Wy_{i}}{\sum_{i}x_{i}^{⊤}Wx_{i}}.$$

We name this procedure as the *stochastic splicing method*, which has been shown to be more accurate and robust than the original linear regression method used in the conventional RNA velocity, possibly due to the inclusion of the additional moments (Bergen et al., 2020). Another major improvement to the RNA velocity methods from (Bergen et al., 2020) is the *dynamical model*, where Bergen et al. derived the solutions for *u* and *s* under the assumption that the promoter has only two states: active and inactive. This assumption is reasonable and proven to be effective but not necessarily true; see above discussion of transcription rates. An EM algorithm is used to iteratively infer the state of the promoter and the latent time for each gene in each cell, and then the solutions are fit to the resulting pseudo-time course of unspliced and spliced RNAs to obtain the kinetic parameters. No steady state assumption is required in this method other than providing a reasonable guess about the initial values for kinetic parameters.

We also developed an alternative procedure, the *negative binomial (NB) distribution method*, based on an observation that in most cases total RNA counts at steady state follow the NB distribution (Grün et al., 2014). With this distribution the variance *σ*^2^ (second central moment) and the mean *μ* satisfy the following relationship:

$$\sigma^{2}=\mu +\phi \mu^{2},$$

where *ϕ* is the reciprocal of the dispersion parameter of NB distribution. Assuming that the number of spliced RNA *s* is an NB-distributed variable, the variance of spliced RNA satisfies:

$$\mathrm{Var}(s)=\frac{\langle u\rangle }{\tilde{\gamma }}+\hat{\phi }\frac{{\langle u\rangle }^{2}}{{\tilde{\gamma }}^{2}},$$

where $\hat{\phi }$ is the estimator of *ϕ* and is computed from:

$$\hat{\phi }=\frac{\mathrm{Var}(s)-\langle s\rangle }{{\langle s\rangle }^{2}}.$$

Put all together, these give the moment conditions for the first and second moments:

$$\{\begin{array}{c} \tilde{\gamma }\langle s\rangle =\langle u\rangle ,\\ {\tilde{\gamma }}^{2}Var(s)=\tilde{\gamma }\langle u\rangle -\hat{\phi }{\langle u\rangle }^{2}. \end{array}$$

A nonlinear least squares optimizer can then be used to solve for $\tilde{\gamma }$ with the above two equations. Note that the two assumptions applied here are: 1) there is a linear relationship between two random variables, which are not limited to the unspliced and spliced RNA, but can also be generalized to labeled or new and total RNA, and 2) one of the variables follows the NB distribution. Therefore, it is straightforward to generalize this method to one-shot labeling data, as will be detailed later.

##### Negative binomial model and bursting properties

A simplistic two-state model was used to model the stochastic expression of genes (Grün et al., 2014):

$$A\overset{k_{\text{off}}}{\to }I,$$

$$I\overset{k_{\text{on}}}{\to }A,$$

$$A\overset{\alpha }{\to }r,$$

$$r\overset{\gamma }{\to }Ø,$$

where *A* and *I* are the active and inactive states of the promoter, respectively, and *r* the number of mRNAs. The first two lines describe the spontaneous switching of the promoter, and the last two lines correspond to the synthesis and degradation of the total mRNA. At steady state, the distribution of *r* follows a negative binomial distribution, where:

$$\langle r\rangle =\frac{\alpha }{\gamma }\frac{k_{\text{on}}}{k_{\text{off}}},$$

$$\varphi =\frac{\gamma }{k_{\text{on}}},$$

where *φ* is the reciprocal dispersion parameter. The burst frequency (BF) is defined as the rate constant for promoter activation, *k*_*on*_, whose reciprocal characterizes the expected time required for the promoter to switch from the inactive state *I* to the active state *A* (Larsson et al., 2019). Following previous work by Larsson et al. (2019), we define the burst size (BS) as the ratio of the synthesis rate *α* and the promoter inactivation rate constant *k*_*off*_. Combining this with the results from the negative binomial, we obtain:

$$BF=k_{\text{on}}=\frac{\gamma }{\varphi },$$

$$BS=\frac{\alpha }{k_{\text{off}}}=\langle r\rangle \frac{\gamma }{k_{\text{on}}}=\langle r\rangle \varphi .$$

##### Estimating absolute RNA velocity for metabolic labeling–based scRNA-seq experiments across various labeling strategies

Because metabolic labeling–based scRNA-seq (time-resolved RNA-seq or tscRNA-seq) measures the synthesis or degradation of labeled RNA within a known period of time in an experimentally programmable manner, it offers a more direct measurement of the kinetics of gene expression than cscRNA-seq. Thus, in principle, it also provides an opportunity to overcome some of the challenges of the cscRNA-seq in RNA velocity estimation. However, it is nontrivial to properly estimate kinetic parameters and compute RNA velocity for tscRNA-seq data with various metabolic labeling approaches, including three general labeling strategies given in Figure 2C: one-shot (the simplest labeling strategy with a single RNA labeling period), kinetics or pulse (a time-series of 4sU or other nucleotide analog treatment to observe the accumulation of metabolically labeled RNA over time), and degradation or chase (a time-series after an extended 4sU or other nucleotide analog treatment period, followed by chase at multiple time points after the wash-out to observe the decay of metabolic labeled RNA over time). Although the exact details of the resultant data vary across different labeling strategies, we found they can be uniformly treated with two different models, **Model 2,** which explicitly considers RNA labeling but not splicing, and **Model 3**, which considers both labeling and splicing (Figure S2A). In the following, we will first briefly introduce these two models, then provide the respective estimation procedures of the three general labeling strategies based on the corresponding models.

In **Model 2**, we take into account labeling (with a labeling correction coefficient *ρ*) but not splicing. The total RNA has a synthesis rate constant *α* and a degradation rate constant *γ*. The labeled RNA has a reduced synthesis rate constant *ρα* but the same degradation rate constant. The ODEs for describing the dynamics of labeled (*l*) and total (*r* = *l* + *o*) are,

$$\dot{l}=\rho \alpha -\gamma l,$$

$$\dot{r}=\alpha -\gamma r.$$

The general solution for the total RNA *r* over time *t* is:

$$r(t)=r_{0}e^{-\gamma t}+\frac{\alpha }{\gamma }\left(1-e^{-\gamma t}\right),$$

where *r*_0_ is the initial concentration of the total RNA *r*. For the labeled RNA, the solution is:

$$l(t)=\frac{\rho \alpha }{\gamma }\left(1-e^{-\gamma t}\right).$$

Note that in this study we rely on a binomial mixture distribution model of background or 4sU-introduced mutation rates, otherwise stated, to quantify the labeled or unlabeled RNA from the observed T-to-C mutation in the final sequencing reads (Jürges et al., 2018). Therefore, assuming labeled RNA (*l*) is well corrected with the binomial mixture model (Jürges et al., 2018), *ρ* is effectively 1. Also see ***Effects of under and overestimation of labeled RNA fraction on tscRNA-seq*** for a detailed discussion on labeling correction coefficient. Furthermore, it is obvious that the transcription rate is not simply a state-dependent constant, as assumed in conventional methods for RNA velocity estimation (Bergen et al., 2020; La Manno et al., 2018) but rather a gene/cell- dependent variable, for it is a function of the labeled (or new, except for degradation labeling experiments) RNA measured for each gene in each cell, i.e., *α* = *γl*(*t*)/(1 − *e*^−*γt*^) (Figure 3E). Together with the unbiased capture of nascent RNA of tscRNA-seq, ***dynamo*** thus overcomes intrinsic challenges to velocity measurements based solely on cscRNA-seq data.

In **Model 3**, we consider both the labeling and the splicing processes. The solutions for labeled, unspliced RNA (*u*_*l*_) and labeled, spliced RNA (*s*_*l*_) are equivalent to those for unspliced and spliced RNA in **Model 1**, with an additional *ρ* modifying the effective transcription rate of the labeled RNA:

$$u_{l}(t)=u_{l,0}e^{-\beta t}+\frac{\rho \alpha }{\beta }\left(1-e^{-\beta t}\right),$$

$$s_{l}(t)=s_{l,0}e^{-\gamma t}+\frac{\rho \alpha }{\gamma }\left(1-e^{-\gamma t}\right)+\frac{\rho \alpha -u_{l,0}\beta }{\gamma -\beta }\left(e^{-\gamma t}-e^{-\beta t}\right).$$

When *β*=*γ*, the solution for *s*_*l*_ is instead:

$$s_{l}(t)=s_{l,0}e^{-\gamma t}+\frac{\rho \alpha }{\gamma }\left(1-e^{-\gamma t}\right)+\left(\beta u_{l,0}-\rho \alpha \right)te^{-\gamma t}.$$

We will omit this special scenario for simplicity in the following sections, although it is included in ***dynamo*** for the sake of completeness and robustness for kinetic parameter estimations.

Below, we detail the respective estimation procedures of the four labeling scenarios given in Figure 2C based on the corresponding models.

Now we will introduce the respective estimation procedures and the corresponding models for each of the three general labeling strategies given in Figure 2C.

###### One-shot experiment

In “one-shot” experiments, there is only one labeling time point, and the splicing process is not explicitly considered. The solution for new RNA in **Model 2** is:

$$l=\frac{\rho \alpha }{\gamma }\left(1-e^{-\gamma t}\right)=\frac{\alpha }{\gamma }k,$$

where *t* is the labeling time and we denote *k* = *ρ*(1 − *e*^*γt*^). When the dynamics of total RNA is at steady state $\left(\dot{r}=\alpha -\gamma r=0\right)$,

$$\;r=\frac{\alpha }{\gamma }=\frac{l}{k}\;\Rightarrow l=kr.$$

Then the parameter *k* can be obtained through a simple linear regression with zero intercept of the first moments of labeled and total RNAs (*l*, *r*), for cells with extreme high expressions of both *l* and *r* (top right corner of the phase plane). This approach effectively replaces *u* and *s* in the original RNA velocity method with *l* and *r*, and was previously reported as the “NTR” (New to Total Ratio) velocity method (Erhard et al., 2019). The NTR velocity can be calculated as:

$$v_{\text{ntr}}=l-kr.$$

Because we used corrected labeling RNAs, i.e. *ρ* ~ 1, the degradation parameter *γ* can be calculated from *k* and the labeling duration *t*:

$$\gamma =-\frac{1}{t}\ln(1-k).$$

Because we obtain *γ*, not the relative $\tilde{\gamma }$ as in the original velocity of spliced RNA, we can calculate the velocity of total RNA with a physical time unit (Qiu et al., 2020a):

$$\dot{r}=\alpha -\gamma r=\frac{\gamma }{k}l-\gamma r.$$

Note that the NTR velocity proposed in (Erhard et al., 2019) is very similar to this method, but scaled by *γ*/*k*, a factor that can differ for individual genes and cancels the unit of time, so it only approximates the true kinetics.

Because in one-shot experiments the labeled and total RNAs are linearly correlated with a slope of *k* = *ρ*(1 − *e*^−*γt*^), and at steady state the total RNA follows the negative binomial distribution, one can easily incorporate second moments using the negative binomial method:

$$\{\begin{array}{l} \;k\langle r\rangle =\langle l\rangle ,\\ k^{2}\mathrm{Var}(r)=k\langle l\rangle -\hat{\phi }{\langle l\rangle }^{2}, \end{array}$$

where,

$$\hat{\phi }=\frac{\text{Var}(r)-\langle r\rangle }{{\langle r\rangle }^{2}}.$$

Then one obtains a more accurate slope *k*, and can be used to compute the velocity of total RNA.

###### Kinetics (pulse) experiment

Two approaches were developed to estimate the RNA turnover rates for the datasets obtained from the kinetics experiment. The first method is a generalization of the “one-shot” method to multiple time points, whereas the second uses a curve fitting strategy which can be also applied to datasets obtained for the degradation experiment. We introduce these two approaches in order:

1. The “two-step” approach (Figure 2C Case 2–4, multi labeling time points/with or without splicing)

With data collected at multiple labeling time points in a kinetics (pulse) experiment, on the phase plane of labeled and total RNA, we find that cells from the same labeling period are distributed on a line whose slope increases as the labeling period increases. We realize that this phenomenon can be explained by the fact that the slope *k* is a monotonically increasing function of the labeling time *t* (see the **“one-shot” method**):

$$k(t)=\rho \left(1-e^{-\gamma t}\right).$$

We then take advantage of this discovery and develop the “two-step” approach, which relies on two consecutive linear regressions to estimate the degradation rate constant *γ* based on **Model 2** (Figure SI2A), and the steady state assumption that $\dot{r}=0$. The first step computes the slope *k* for the labeled (*l*) and total (*r*) RNA for different labeling time *t*, based on the linear relationship (see the **“one-shot” method**):

l=kr.

When labeling correction coefficient is close to one, from *k*(*t*) = 1 − *e*^−*γt*^, it is apparent that the slope increases with longer labeling time and asymptotically approaches one. Rearranging this equation, we have:

γt=−ln(1−k).

A linear relationship exists between the labeling time *t* and the quantity −ln(1 − *k*). In the second step, we then estimate the parameter *γ* using a simple linear regression of *t*. The total RNA velocity is again:

$$\dot{r}=\frac{\gamma }{k}l-\gamma r.$$

Note that the “two-step” approach can be regarded as a generalization of the above “one-shot” method for one-shot labeling experiments to kinetics experiments with multiple labeling time points. The negative binomial method can also be applied here in the first step to achieve a more robust estimation of the slope *k*. We note that not every single gene in the dataset may follow this kinetics, and in general we use R-square of the “two-step” model fitting to select genes with confident fittings for downstream analysis.

2). Curve fitting methods (Figure 2C Case 2–4, multi labeling time points/with or without splicing)

When single-cell kinetics (pulse) or degradation (chase) data using RNA metabolic labeling, e.g., scEU-seq or scNT-seq (Battich et al., 2020; Qiu et al., 2020a), at multiple time points are available (Figure 2C Case 2), it is possible to estimate the kinetic parameters (*α*, *β*, and *γ*) for each gene using nonlinear least-squares methods. In general, given *m* experimental data points, *y*^(1)^,*y*^(2)^,…,*y*^(*m*)^, at time points *t*^(1)^,*t*^(2)^,…, *t*^(*m*)^, the least-squares fitting method finds a set of parameters ***θ*** that minimize the following loss function:

$$L(\theta )=\sum_{i=1}^{m}{\left(y^{\left(i\right)}-x\left(t^{\left(i\right)},\theta \right)\right)}^{2},$$

where *x*(*t*, ***θ***) is the solution of the ODEs at the time point *t*, given parameters ***θ***. When there are multiple species (i.e., unspliced labeled *u*_*l*_, spliced labeled *s*_*l*_, unspliced unlabeled *u*_*u*_, or spliced unlabeled *s*_*u*_ RNAs) quantified from the experiment, we cast the ODEs into a matrix form while the composite loss function is the summation for loss function of all species, and weights can be added to the loss function to adjust the importance of each species. For example, a higher weight is assigned to the labeled than the unlabeled species (2:1 by default) for the kinetics experiment, because the unlabeled species does not strictly follow the degradation kinetics due to imperfect labeling:

$$L(\theta )=\sum_{j=1}^{n}\omega_{j}L_{j}(\theta )=\sum_{j=1}^{n}\omega_{j}\sum_{i=1}^{m}{\left(y_{j}^{\left(i\right)}-x_{j}\left(t^{\left(i\right)},\theta \right)\right)}^{2}.$$

This general procedure is applied to all following curve-fitting methods; the key is to find solutions of each species for various RNA labeling strategies.

We used Latin hypercube sampling to randomly initialize a set of values of ***θ*** in a predetermined range (see ***Estimation of Parameter Ranges for Curve Fitting Methods***) as the initial guesses for the parameters ***θ*** required by the nonlinear least squares optimizer.

In kinetics experiments, the samples are collected after a short period of 4sU (or other nucleotide analogs) labeling. At the beginning of the experiment, the concentrations for labeled RNA, unspliced labeled and spliced labeled RNA, are zero (*l*_0_ = *l*(0) = 0, *u*_*l*,0_ = *u*_*l*_(0) = 0 and *s*_*l*,0_ = *s*_*l*_(0) = 0). During the labeling process, because we assume that the labeling period is much shorter than the time scale of the biological process of interest, transcriptional rates are treated as constant in all cells. Therefore, based on the solutions of **Model 3,** the abundance of labeled, unspliced labeled and spliced labeled RNA increase over time:

$$\;u_{l}(t)=\frac{\alpha }{\beta }\left(1-e^{-\beta t}\right),\;s_{l}(t)=\frac{\alpha }{\gamma }\left(1-e^{-\gamma t}\right)+\frac{\alpha }{\gamma -\beta }\left(e^{-\gamma t}-e^{-\beta t}\right).$$

With sufficient sampling of the labeling time points (at least three), all three kinetic parameters can be estimated in theory. Because cells at different states may have different transcription rates, clustering can be performed first and the fitting is done for each cluster to derive cluster or cell-type specific kinetic rates (Battich et al., 2020; Qiu et al., 2020a). The above solutions are often insensitive to variations in *γ*, and the read counts for the unspliced RNA are unreliable for genes with fast splicing rates, so it is optional to provide further constraints by including the kinetics of unlabeled or old, unlabeled spliced and unlabeled unspliced RNA, in the curve-fitting procedure. The unlabeled RNA in kinetics experiments mostly follow the degradation kinetics, if the labeling efficiency is close to 1 (see ***Effects of under and overestimation of labeled RNA fraction on tscRNA-seq***), and the solutions are more sensitive to *β* and *γ* than those of the labeled species:

$$\;u_{u}(t)=u_{u,0}e^{-\beta t},\;s_{u}(t)=s_{u,0}e^{-\gamma t}-\frac{\beta u_{u,0}}{\gamma -\beta }\left(e^{-\gamma t}-e^{-\beta t}\right).$$

The spliced RNA velocity can be computed as before:

$$\dot{s}=\beta u-\gamma s.$$

The solution for *u*_*l*_ above also allows us to compute the velocity for unspliced RNA in individual cells:

$$\dot{u}=\alpha -\beta u=\frac{\beta u_{l}}{1-e^{-\beta t}}-\beta u.$$

If no splicing data are available, the solution for **Model 2** can be used:

$$l(t)=\frac{\alpha }{\gamma }\left(1-e^{-\gamma t}\right).$$

The total RNA velocity can be computed either for each cluster, where *α*_*c*_ denotes the transcription rate constant of cluster *c*:

$$\dot{r}=\alpha_{c}-\gamma r,$$

or for individual cells:

$$\dot{r}=\alpha -\gamma r=\frac{\gamma l}{1-e^{-\gamma t}}-\gamma r.$$

The velocity for new RNA can be computed in a similar way:

$$\dot{l}=\alpha -\gamma l=\frac{\gamma l}{1-e^{-\gamma t}}-\gamma l.$$

There is, however, a practical issue when using curve-fitting methods with **Model 1** for data obtained from the kinetics experiments. Because the current labeling time of a tscRNA-seq kinetics experiment typically requires at least 1 hour (because of the low sensitivity of single- cell methods), which is much longer than the time scale of RNA splicing (usually on the scale of minutes), the labeling kinetics do not have sufficient time resolution for reliable estimation of the splicing rate constant *β*. We can circumvent this by first computing $\tilde{\gamma }=\gamma /\beta$ from the total unspliced (*u* = *u*_*l*_ + *u*_*u*_) and spliced RNA (*s* = *s*_*l*_ + *s*_*u*_) using the conventional RNA velocity method. Then, we can use either model to estimate the actual degradation rate constant *γ*, and the splicing rate constant is simply given by:

$$\beta =\gamma /\tilde{\gamma }.$$

With this, we can then estimate absolute RNA velocities for total, spliced, unspliced, and new RNAs according to the model and data available. Note that a similar procedure can also be applied to relative kinetic parameters estimated with the dynamical method from (Bergen et al., 2020) that generalizes to the non–steady-state assumption, and used to scale them to absolute values.

###### Degradation (chase) experiments

In degradation experiments (Case 3 in Figure 2C), samples are chased after an extended 4sU (or other nucleotide analog) labeling period and the wash-out to observe the decay of the abundance of the (labeled) unspliced *u*_*l*_ and spliced *s*_*l*_ RNA decay over time. The process can be formulated as below (the zero in the subscript indicates the initial condition):

$$\;u_{l}(t)=u_{l,0}e^{-\beta t},\;s_{l}(t)=s_{l,0}e^{-\gamma t}-\frac{\beta u_{l,0}}{\gamma -\beta }\left(e^{-\gamma t}-e^{-\beta t}\right).$$

These two equations can be substituted into the loss function, and we obtain splicing rate constant *β* and degradation rate constant *γ* using the nonlinear least squares. The (labeled and unlabeled) spliced RNA velocity is then given by:

$$\dot{s}=\beta u-\gamma s.$$

Although the unlabeled RNAs (*u*_*u*_, *s*_*u*_) indeed increase over time due to transcription, cell-wise transcription rates *α* cannot be directly estimated from such experiments because each cell has different transcription activity. However, with a two-state promoter stochastic expression model, we can assume a universal *α*_on_ and *α*_off_ for all cells, similar to the dynamical model (Bergen et al., 2020).

For degradation experiments without splicing data, the solution of **Model 2** is used. The abundance of labeled RNA (*l*) follows the first-order decay kinetics (Qiu et al., 2020a):

$$l=l_{0}e^{-\gamma t}.$$

Note that this method has the same drawback as the curve-fitting method for experimental kinetics data, i.e., the estimation of *β* can be unreliable if the chasing time resolution is much larger than the time scale of splicing. Again, one may combine the curve fitting with the conventional RNA velocity method and obtain a more accurate splicing rate constant *β* and RNA velocities.

##### Estimation of parameter ranges for curve fitting methods

To overcome the local optima of the cost function and speed up parameter estimation, we need to have good guesses of parameters and the valid ranges of those parameters. A set of parameter ranges are used for initial parameter value sampling and providing upper and lower bounds for optimizers to avoid unrealistic results. The “guesstimated” values *θ*_0_ for specific parameters are first determined, according to the specific labeling strategy used. The range of the parameters is then simply set to be (0, 100 *θ*_0_). The methods for obtaining “guesstimations” are different for each parameter:

1. Kinetics experiments
  If the RNA dynamics are far from steady state and degradation is negligible, then the amount of newly synthesized RNA is proportional to the labeling time:
$$l_{t}∼\alpha t,$$
  where *l*_*t*_ = *n*(*t*), i.e. the number of copies of new RNA at labeling time *t*. Thus, the guesstimated *α* is simply the averaged ratio of new RNA and labeling time. The degradation rate constant can be roughly estimated from the old RNA:
$$\gamma ∼\frac{1}{t}\ln\frac{o_{0}}{o_{t}}.$$
  The splicing rate constant is estimated in a similar manner:
$$\beta ∼\frac{1}{t}\ln\frac{u_{u}(0)}{u_{u}(t)}.$$
2. Degradation experiments
  The guesstimated values for the initial conditions, including *l*_0_, *u*_*l*,0_, and *s*_*l*,0_, are simply the average abundance of labeled RNAs across all cells belonging to the initial labeling time point. The degradation rate constant is guesstimated with the labeled RNA, using a equation similar to the one for kinetics experiments:
$$\gamma ∼\frac{1}{t}\ln\frac{l_{t}}{l_{0}}.$$
  The splicing rate constant is estimated with:
$$\beta ∼\frac{1}{t}\ln\frac{u_{l}(t)}{u_{l}(0)}.$$

##### Goodness of fit for linear regression and curve fitting methods during kinetic parameters estimation

For linear regression models, given the data and model predictions ${\left\{x_{i},y_{i}\right\}}_{i=1}^{n}$ for *n* cells, the goodness of fit is determined using the standard R-squared:

$$R^{2}=1-\frac{{\sum_{i=1}^{n}\left(x_{i}-y_{i}\right)}^{2}}{\sum_{i=1}^{n}{\left(x_{i}-\bar{x}\right)}^{2}},$$

where $\bar{x}$ is the mean of data. For curve fitting methods, the Gaussian log-likelihood is used as a measure for goodness of fit. Given the data and model predictions ${\left\{x_{i},y_{i}\right\}}_{i=1}^{k}$ of *k* species, where each *x*_*i*_ and *y*_*i*_ is a vector of model predictions for *m* time points, the Gaussian log-likelihood is:

$$\ln G\left(x_{1},x_{2},\ldots ,x_{k}|y_{1},y_{2},\ldots ,y_{k}\right)=-\frac{n}{2}\ln(2\pi )-\sum_{i=1}^{k}\ln\left(\sigma \left(x_{i}\right)\right)-\sum_{i=1}^{k}\frac{1}{2}{\left\|{\bar{x}}_{i}-y_{i}\right\|}^{2},$$

where $\bar{x}$ and *σ*(***x***) are the mean and standard deviation of ***x***, respectively. To balance the numerical difference between species, the data and model predictions are normalized by the maximal value of data for each species.

##### Impacts of dimensions of rate constants on RNA velocity

The rate law connects the rate of a reaction and concentrations of involved (bio)chemical species. As an example, the rate law for a first-order reaction that generates a product *A* is:

v=k[A],

where *v* is the reaction rate, *k* the first-order rate constant, and [*A*] is the concentration of product *A*. Because the time scale of a first-order reaction is often characterized by the reciprocal of the rate constant (also known as the “time constant”, or “half-life” up to a factor of ln 2 for first-order degradation, i.e., *t*_1/2_ = ln 2/*k*), “rate” and “rate constant” are often used interchangeably in certain contexts (La Manno et al., 2018; Bergen et al., 2020). They are, however, quantities with different dimensions, and this often leads to confusion, especially for RNA velocity methods. For scRNA-seq data, we assume a constant cell volume (see below for more discussions on impacts of the cell volume and others), and the concentration, whose dimension is usually the quantity (or the copies of RNA species) of gene *A* per unit volume, is replaced by the copy number of *A*. Therefore, the dimension of the reaction rate *v* is copy number of molecules per unit time (denoted as *N*/*T* ), and that of the first-order rate constant is one per unit time (1/*T*). For a zeroth-order reaction, the rate constant is also the reaction rate, and therefore they share the same dimension.

In the context of RNA velocity, the velocities of unspliced and spliced RNA for a gene are essentially net reaction rates for the production and depletion of unspliced and spliced RNA, with the dimension of *N*/*T*. Because RNA splicing and degradation are first-order reactions, *β* and *γ* are first-order rate constants with dimension 1/*T*. In the original RNA velocity method (La Manno et al., 2018), the degradation rate constant *γ* is scaled by the splicing rate constant *β*, so the relative rate constant $\tilde{\gamma }$ is dimensionless. The resulting RNA velocity $v=u-\tilde{\gamma }s$ does not have the dimension of reaction rates *N*/*T*, but rather only the number of molecules (*N*), and thus the “velocity” is relative to the splicing rate constant *β*. Consequently, suppose that one obtains a small relative RNA velocity for a gene, the actual change in the copy number of spliced RNA per unit time can be large if the splicing rate is fast.

The transcription of unspliced RNA is assumed to be a zeroth-order reaction, so *α* is a zeroth-order rate constant with the dimension *N*/*T*. Note that RNA transcription is not an elementary reaction in which products are formed in a single step, but instead a complex reaction with multiple steps involving various trans- and cis-elements. The zeroth-order rate constant *α* is thus an apparent rate constant under a reduced reaction scheme that lumps many intermediate steps, which are in fact regulated by a variety of internal and external signals. As a result, the transcription rate constant *α* is a function of cell state in the gene expression space. This has also been shown to be the case for splicing and degradation rate constants (Battich et al., 2020) although it is reasonable to assume those are constants as we and others did (La Manno et al., 2018; Bergen et al., 2020).

Here, we would like to provide some thoughts on cell volume. Because typical scRNA-seq data contain no cell volume information, as a zeroth-order approximation we assume a constant cell volume for all cells. This approximation does not affect the sign of estimated RNA velocity because all RNA species in one cell are affected equally. With cell volume information available together with the expression state (e.g., from imaging based methods), it is straightforward to incorporate cell-specific volume information in our parameter estimation procedure.

We also want to comment that in practice, we additionally assume that all cells share the same total RNA content. In our preprocessing steps, we scale the total UMI counts in each cell to 10,000 molecules, similar to many other scRNA-seq analysis toolkits. The normalized gene expression in each cell can be regarded as the fraction of total RNA content occupied by each gene. This normalization scheme is believed to help remove library size differences incurred during library construction and sequencing (Love et al., 2014).

##### Correcting RNA velocity flow by removing genes with low gene-wise confidence in the phase plane

In some scenarios, we may find unexpected wrong velocity backflow from your RNA velocity analysis. To diagnose those cases, we can identify genes showing up in the wrong phase portrait position that may contribute to the wrong flow direction. We can then remove those genes to correct velocity vectors. This requires some prior knowledge about the progenitor and terminal cell types in the system. The underlying rationale boils down to the following scenarios (Figure S3D):

1. If the expression of a particular gene in the progenitor is low, it should start to increase as cells differentiate from progenitor to terminal cell states. There should be progenitors that are above the steady-state fitting line in the phase plane. However, if most of the progenitor cells are located below the line, their velocities are negative, leading to reversed vector flow.
2. If the expression of a particular gene in progenitors is high, it should start to decrease as the cells differentiate to terminal cell states. There should be progenitors that are below the steady-state fitting line. However, if most of the progenitors are located above the steady-state line, their velocities are positive, leading to reversed vector flow.
3. Similar reasoning can be applied to the mature cell states.

Thus, we design a heuristic algorithm to quantify the confidence of each gene by assessing whether it obeys the above constraints:

- We first assess whether, when each progenitor state differentiates into each terminal cell state, a gene is in the induction or repression phase based on the shift of the median gene expression between these two states. If it is in the induction phase, cells should mostly have positive or close to zero velocity (e.g. a small negative velocity threshold) and vice versa. Those thresholds can be provided by the users or inherited from the default values provided by ***dynamo***.
- 1 - fraction of cells having velocity passing those thresholds in each state is then used as a measure of velocity confidence.

Note that this heuristic method requires one to provide meaningful progenitor groups and mature cell groups, and the thresholds of velocity. In particular, the progenitor groups should in principle have cells going out (transcriptomically), whereas mature groups should end up in a different expression state, and there are intermediate cells going to the dead end cells in each terminal group (or most terminal groups).

##### Cell-wise confidence of RNA velocity vectors

Several confidence metrics for cell-wise velocity vectors are implemented in ***dynamo***. By default it uses the Jaccard index, which measures how well each velocity vector meets the geometric constraints defined by the local neighborhood structure (Ma et al., 2017). The Jaccard index is calculated as the fraction of the number of the intersected set of nearest neighbors from each cell at the current expression state (***x***) and that from the future expression state (***x*** + ***v***) over the number of the union of these two sets, namely:

$$J=\frac{S\left(x_{i}\right)\;\cap \;S\left(x_{i}+v_{i}\right)}{S\left(x_{i}\right)\;\cup \;S\left(x_{i}+v_{i}\right)},$$

where *x*_*i*_, *v*_*i*_, *S*(*x*_*i*_) and *S*(*x*_*i*_ + *v*_*i*_) are respectively the current expression state for cell *i*, the current velocity vector for cell *i*, the set of nearest neighbor cells for cell *i* based on the current expression states (***x***), and the set for nearest neighbor cells for cell *i* based on the future expression states (***x*** + ***v***).

The cosine or correlation method is similar to that used by *scVelo* (Bergen et al., 2020) and can be used to quantify the local consistency of the velocity flow for each cell.

##### Dynamo: 2) Reconstruction of single-cell vector field functions

###### Hidden variables of single-cell transcriptomic datasets affect cell state and dynamics quantification, and vector field reconstruction

There are three fundamental assumptions in the modeling of cell dynamics in the gene expression space, and the reconstruction of the vector field from single-cell transcriptomic data: first, the transcriptome is complete (or sufficient to specify cell states); second, the trajectories of cell transitions in the gene expression space are continuous and differentiable; and third, the dynamics can be described by a set of memoryless equations, i.e., the temporal propagation of the system depends only on the present state, but not those at prior times. Here, we provide some justifications for those assumptions and discuss the limitations of the vector field reconstruction. Moreover, we discuss the sources of noise in RNA velocity data and how their effects are minimized in vector field reconstruction.

Generically, one can represent the internal state of a cell by the expression levels (and even spatial distributions) of intracellular molecular species, e.g., spliced or unspliced RNAs. Mathematically, one represents the cell state as a vector *z* = {***x***, ***y***}, where ***x*** represents the measured spliced and unspliced transcripts, and/or labeled and total RNA in the case of labeling-based scRNA-seq experiments, and ***y*** represents all other unmeasured species such as the proteome and epigenome. It should be noted that ***x*** can be different from the raw RNA counts (denoted as *u*, *s*, *l*, and *r*, for unspliced, spliced, labeled, and total RNA, respectively), as in many cases the domain of the vector field is the size-factor normalized and then logarithm-transformed transcripts (or top principal components). Let us assume that one can describe the dynamics of a cell by a set of stochastic differential equations (or other forms such as discrete dynamics, for which the following discussions still hold),

(1) $$\frac{\text{d}x}{\text{d}t}=F(x,y,\mu (t))+\zeta_{x}(x,y,t),$$

(2) $$\frac{\text{d}y}{\text{d}t}=G(x,y,\mu (t))+\zeta_{y}(x,y,t).$$

The functions ***F*** and ***G*** form a vector field in the full space that describes interactions among intracellular species, influence from extracellular environmental factors (*μ*) including external stimuli and the extracellular secretome, and direct interactions with neighboring cells. Biologically, we expect that different layers of gene regulation, e.g., the proteome and transcriptome, are coupled. The extracellular factors *μ* are in general explicitly time dependent. The terms *ζ*_***x***_ and *ζ*_***y***_ refer to random noise, and we assume them to be white noise with zero means. Much of the effort in this study focuses on the reconstruction of ***F***, and there are two theoretical issues that must be considered when reconstructing the vector field from single-cell transcriptome data alone. First, in a typical scRNA-seq experiment, only ***x*** is measured, and the other variables are hidden. Second, a cell is generally subject to a time-varying extracellular environment.

For simplicity, we restrict ourselves to the case that external stimuli are constant and spatially uniform, whereas in a more general situation the vector field is time-dependent. We also treat direct and indirect cell–cell interactions in a mean-field sense instead of treating the many-body cell–cell interaction problem explicitly. With single-cell multi-modality co-assays that are also augmented with spatial and temporal resolution (Liu et al., 2020), our framework will allow us to explicitly account for “hidden variables”.

If the system dynamics are deterministic, i.e., *ζ*_***x***_ *= ζ*_***y***_ = 0, cells evolve along a manifold *M* embedded in the state space of {***x***, ***y***} (the solid curve in Figure S4A). If one wants to define the metaphorical Waddington’s epigenetic landscape, it should be defined on this manifold. In the case that ***x*** and ***y*** are tightly coupled, i.e., *x* and *y* are not independent variables, and the manifold can be parameterized solely by ***x***, then a cell state can be well-represented by the transcriptome alone. Mathematically, this means that we assume that the manifold in the full space and its projection to the ***x*** space are homeomorphic. In Figure S4A, ***x*** and ***y*** are coupled at ***x***_*a*_ and ***x***_*b*_, while at ***x***_*c*_ the cell state cannot be uniquely specified solely by ***x***.

The presence of stochasticity loosens the coupling. Instead of moving strictly on the manifold, the population of cells follows an evolving probability distribution *ρ*(***x***, ***y***, *t*) centered at the manifold (represented as the gradient around *M* in Figure S4A). Mathematically, a transcriptome-based quantity *O*(***x***), e.g., the number of unspliced (*u*) and spliced (*s*), or labeled (*l*) and total (*r*) RNA, should be understood as being projected to the subspace of ***x***, i.e., averaged over the hidden variables,

(3) $$\langle O(x)\rangle =\frac{1}{Z}\int \text{d}x^{\prime }\text{d}y^{\prime }O\left(x^{\prime },y^{\prime }\right)\rho \left(x^{\prime },y^{\prime },t\right)\delta \left(x^{\prime }-x\right),$$

where *Z* is the normalization factor $Z=\int \text{d}x^{\prime }\text{d}y^{\prime }\rho \left(x^{\prime },y^{\prime },t\right)\delta \left(x^{\prime }-x\right)$, and *δ* is Dirac’s delta function, which sifts out the ***x*** among all possible ***x*** within an integral. In the case of time scale separation between transcription and other slower processes (translation, epigenetic modification, etc.), one may further assume that ***x*** reaches quasi-steady-state for a given set of ***y***, and one can expect that *ρ*(***x***, ***y***, *t*) ≈ *ρ*_1_(***x***_*ss*_(***y***)) *ρ*_2_(***y***, *t*) also varies slowly in time.

In practice, the above average is typically performed by averaging *k* neighboring cells in the state space, weighted with a specific kernel function (see ***First moment smoothing method in the generalized methods of moments***, and (La Manno et al., 2018; Bergen et al., 2020; Li et al., 2021)):

(4) $$\langle O(x)\rangle =\sum_{i=1}^{k}O\left(x_{i},y_{i}\right)K\left(\left|x_{i}-x\right|\right),$$

with the data set sampled from *ρ*(***x***, ***y***, *t*), and $\sum_{i}K\left(\left|x_{i}-x\right|\right)=1$. Note that *K* is often chosen as a fast-decaying function (Gaussian kernel) to |***x***_*i*_ − ***x***|, compared to *ρ*(***x***, ***y***, *t*), or a *k*-nearest-neighbor step function with a sufficiently small neighborhood (30 by default in ***dynamo***), compared to the total number of cells in the dataset. In fact, the RNA velocity is also such an average, for example:

$$\langle v_{s}\rangle =\langle u-\tilde{\gamma }s\rangle =\langle u\rangle -\tilde{\gamma }\langle s\rangle ,$$

$$\langle v_{\text{ntr}}\rangle =\langle l-kr\rangle =\langle l\rangle -k\langle r\rangle .$$

Note that here *k* = 1 − *e*^−γt^ (see ***Estimating absolute RNA velocity for metabolic labeling–based scRNA-seq experiments across various labeling strategies***). This approximates the following average in the continuous domain:

(5) $$\langle O(x)\rangle =\frac{1}{Z}\int \text{d}x^{\prime }\text{d}y^{\prime }\;O\left(x^{\prime },y^{\prime }\right)\rho \left(x^{\prime },y^{\prime },t\right)\kappa \left(\left|x^{\prime }-x\right|\right)\;\;=\frac{1}{Z}\int \text{d}x^{\prime }\;\kappa \left(\left|x^{\prime }-x\right|\right)\rho \left(x^{\prime },t\right)\int \text{d}y^{\prime }\;O\left(x^{\prime },y^{\prime }\right)\rho \left(y^{\prime }|x^{\prime },\;t\right),$$

where *κ* is the continuous analogue of *K*, and:

$$Z=\int \text{d}x^{\prime }\text{d}y^{\prime }\kappa \left(x^{\prime }-x\right)\rho \left(x^{\prime },y^{\prime },t\right)\;\;=\int \text{d}x^{\prime }\kappa \left(x^{\prime }-x\right)\rho \left(x^{\prime },t\right)\int \text{d}y^{\prime }\rho \left(y^{\prime }|x^{\prime },t\right)\;\;=\int \text{d}x^{\prime }\kappa \left(x^{\prime }-x\right)\rho \left(x^{\prime },t\right).$$

Note that in the discrete version (Eq. 4), because (***x***_**i**_, ***y***_**i**_) are supposedly sampled from the distribution, the probability density *ρ*(***x***, ***y***, *t*) is implicitly included in the summation. Comparison of the continuous average (Eq. 5) with the projection (Eq. 3) makes it clear that the fast-decaying kernel *K*/*κ* serves as a softened Dirac’s delta function, which sifts through all possible ***x*** and keeps those that are close to ***x*** (purple gradients in Figure S4A). The second integral in Eq. 5 performs the projection, and an Taylor expansion of *O*(***x***, ***y***) around the mean of *ρ*(***x***, ***y***, *t*), $\bar{y}$, reveals the dependency of the error on *ρ*(***x***, ***y***, *t*):

$$\int \text{d}y^{\prime }O\left(x^{\prime },y^{\prime }\right)\rho \left(y^{\prime }|x^{\prime },\;t\right)=\int \text{d}y^{\prime }\left\{O\left(x^{\prime },\bar{y}\right)+\frac{\partial O}{\partial y^{\prime }}\cdot \left(y^{\prime }-\bar{y}\right)+O\left({\left(y^{\prime }-\bar{y}\right)}^{2}\right)\right\}\rho \left(y^{\prime }|x^{\prime },\;t\right)\;\;=O\left(x^{\prime },\bar{y}\right)+\frac{\partial O}{\partial y^{\prime }}\cdot {\left(y^{\prime }-\bar{y}\right)}_{y^{\prime }|x^{\prime },t}+\ldots$$

When ***x*** and ***y*** are tightly coupled, *ρ*(***y***|***x***, *t*) is a very narrow unimodal distribution (***x*** = ***x***_*b*_ in Figure S4A), and the higher order terms depending on $\left|y-\bar{y}\right|$ vanish. The projection approximates $O(x,\bar{y})$ with minimal error. As the coupling between ***x*** and ***y*** weakens, the higher-order terms become no longer negligible, and cells whose hidden variables largely deviated from $\bar{y}$ are included, leading to error in the projection (***x*** = ***x***_*a*_ in Figure S4A). In the worst case where there is no coupling between ***x*** and ***y***, *ρ*(***y***|***x***, *t*) becomes multimodal and the projection is compromised (***x*** = ***x***_*c*_ in Figure S4A).

Due to stochasticity in gene expression and technical errors from scRNA-seq experiments, the observable *O*(***x***, ***y***) are almost always measured with errors, in addition to the error introduced by the hidden variables. The stochastic differential equations in Eq. 1 and Eq. 2 correspond to a Fokker–Planck equation, describing the time evolution of the probability distribution *ρ*(***y***|***x***, *t*):

$$\frac{\partial \rho (z,t)}{\partial t}=\nabla \cdot (-A(z)\rho (z,t)+D(\;z)\cdot \nabla \;\rho (z,t)),$$

where ***D***(*z*) is the diffusion tensor associated with the white noises. ***A***(*z*) is the *drift*, which concatenates ***F*** and ***G*** (Kampen, 2007):

$$A(z)=\lim_{\text{Δ}t\to 0}\frac{\langle \text{Δ}z\rangle }{\text{Δ}t}=\lim_{\text{Δ}t\to 0}{\left(\frac{\langle \text{Δ}x\rangle }{\text{Δ}t},\frac{\langle \text{Δ}y\rangle }{\text{Δ}t}\right)}^{⊤}=(F(z),\;\;G(z)).$$

***A*** dictates the evolution of cells in the gene expression space and can be understood as the averaged velocity of both ***x*** and the unmeasured ***y***. The velocity vectors obtained using the first moment averaging (Eq. 4) approximate $\lim_{\Delta t\to 0}\Delta x/\Delta t$. The vector field reconstructed based on the first moment average is then essentially (***F***(***x***)), the projection of ***F***(*z*) on ***x***. During vector field reconstruction, the sparseVFC algorithm minimizes noise by optimizing the sum-of-squares of the difference between the vector field and the data, as well as detecting outliers based on a Bayesian approach (see ***Outlier detection in vector field reconstruction***). Although this study focuses on the reconstruction of the vector field, which corresponds to the deterministic term (drift) of Eq. 1, one can simulate multiple trajectories given some initial conditions and reasonable assumptions on the noise terms using the numerical Ito or Stratonovich integrals. In ***dynamo***, we provide such a possibility by leveraging the *sdeint* python package.

###### Caveats on vector field reconstruction

Note that the vector field, defined as $\dot{x}(t)=f(x(t))$, does not allow two trajectories to cross each other. Therefore, the input velocity vectors for vector field reconstruction should not have many cells with very similar gene expression states but inconsistent velocity vectors. This can happen either when the data have strong hidden variable effects (case *c* in Figure S4A), or when there are potential strong batch effects between different batches of datasets. We expect that the hidden variable issue can be alleviated by single cell multi-omics to capture a more holistic view of cell states, improvements in RNA capture rate, and a reduction in sequencing cost. Further efforts by our group or others will be needed to address the second issue so that we can correct batch effects while performing RNA velocity and vector field reconstruction.

Note that our vector field reconstruction is applicable to both the cscRNA-seq and the tscRNA-seq data. Because the RNA velocity from cscRNA-seq data is relative and scaled by the splicing rate constant *β* for each gene, we explore whether the velocity directionality would be affected by this scaling with relative RNA velocity, especially in the UMAP space. Randomly scaling the velocity vector by a positive value on a few cscRNA-seq data, sampled from the uniform distribution (0, 10) for each gene, however, does not change the velocity vector directionality in UMAP (data not shown), indicating that the sign of velocity is the most important information for revealing the directionality of RNA velocity, especially when projected to a lower dimension. This result may explain why the conventional RNA velocity method, although relative, still proves useful in revealing the directionality of cell fate transitions. When the RNA velocity estimates are relative, the resultant vector field and differential geometry quantities are also relative. In this study, we demonstrated that importance of absolute vector field analyses with the cell-cycle dataset from (Battich et al. (2020)). We found that even when the direction of relative splicing RNA velocity flow is correct, the downstream differential geometry analyses can lead to misleading results, e.g., although all the top acceleration genes from the absolute RNA velocity based vector field are associated with the cell cycle, a considerable number of top genes from the relative RNA velocity are not.

###### Robust reconstruction of continuous velocity vector field functions from sparse single cell transcriptomic measurements

In the second and third stages of our ***dynamo*** model framework, we robustly learn a continuous vector field function of single cells from the input discrete, sparse, and noisy single-cell velocity vector samples. We also bring in predictive dynamical system methods and differential geometry analyses to improve the interpretability of the “black box” machine learning powered vector field functions, thus marrying the power of advanced machine learning (ML) approaches in functional approximation with the interpretability of dynamical systems formulations.

###### Vector field of expression space in single cells

In classical physics, including astronomy, fluidics and aerodynamics, velocity and acceleration vector fields are used as fundamental tools to describe motion or external force of objects, respectively. In general, a vector field can be defined as a vector-valued function ***f*** that maps any point (i.e. expression state of a cell) ***x*** in a (subset of) *d* dimensional (gene expression) space to a vector ***v*** (e.g. the RNA velocity vectors) in the same space, i.e., ***v***=***f***(***x***). Thus, RNA velocity estimates (Bergen et al., 2020; La Manno et al., 2018) from single cells can be formally treated as samples in the velocity vector field. In two or three dimensions, a vector field is often visualized as a quiver plot, where a collection of arrows with a given magnitude and direction is drawn. Assuming an asymptotic deterministic system, the trajectory of the cells travelling in the gene expression space follows the vector field and can be calculated using numerical integration methods, e.g., the Runge–Kutta algorithm. In two or three dimensions, a streamline plot can be used to visualize those integration paths. For high-dimensional vector fields, it is challenging to present all information at once, and multiple quantities are required to reveal different features of the vector field. As we will show later, differential geometry offers many such quantities, each allowing us to capture some but not all dynamical features of the vector field.

###### Vector field reconstruction from sparse, noisy single-cell expression and velocity samples

With csc- or tscRNA-seq data and the computational framework mentioned above, in principle we can obtain vector field samples in either the unspliced, spliced, new, or total RNA space, depending on the exact experiment, labeling strategy, and estimation method. High-dimensional velocity vectors are often projected onto top PCA (principal component analysis) space or two- or three-dimensional UMAP (Uniform Manifold Approximation and Projection) space (Bergen et al., 2020; La Manno et al., 2018). In order to go beyond sparse velocity samples to continuous vector field functions in full gene expression space, we build on some recent advances in vector valued function approximation to scalably, efficiently, and robustly learn the transcriptomic vector field (see Box 2 and below) from noisy and sparse samples of single-cell states and velocity estimates. Our reconstruction works in projected PCA or UMAP space, or even in the full gene-expression space. When it is reconstructed in low-dimensional space, the learned vector field can be projected back to the original transcriptomic space for gene-specific velocity and differential geometry analyses.

###### Vector Field Reconstruction in the Reproducing Kernel Hilbert Space

To formally introduce the problem of velocity vector field learning in the context of scRNA-seq, we consider a set of pairs of cell expression states $x\in X\subset {\mathbb{R}}^{d}$ and RNA velocities $v\in V\subset {\mathbb{R}}^{d}$, i.e. ${\left\{x_{i},v_{i}\in X\times V\right\}}_{i=1}^{n}$, where *n* is the number of cells, and *d* is the dimension (number of genes or number of principal components) of the cell state space. We assume that the measured single-cell RNA velocity is sampled from a smooth, differentiable vector field that assigns each cell expression state ***x*** with an RNA velocity vector ***v***. Normally, single-cell RNA velocity measurements are results of biased, noisy, and sparse sampling of the cell expression state space. Therefore, the goal of velocity vector field reconstruction is to robustly learn a mapping function ***f***, which outputs an RNA velocity vector ***v***, based on the observed data ${\left\{x_{i},v_{i}\in X\times V\right\}}_{i=1}^{n}$, under certain smoothness constraints (Ma et al., 2013). Ideally, the mapping function ***f*** should recover the true velocity vector field on the entire domain X and can be used to predict the true dynamics in regions of expression space that are not sampled. The discussion introduced above is based on the velocity vector field, but it can be similarly extended into any general vector field, e.g., an acceleration vector field (Gorin et al., 2020).

Intuitively, the loss function for the search of an optimal vector field function f* can be written in a least-squares fashion:

$$\text{Φ}(f)=\sum_{i=1}^{n}p_{i}{\left\|v_{i}-f\left(x_{i}\right)\right\|}^{2},$$

where *p*_*i*_ is a weight deciding the importance of the *i*-th data point in the loss function. However, it is not a trivial task to minimize the above loss function with respect to a function ***f***. Approximating vector-valued functions in a sparse reproducing kernel Hilbert space (RKHS) has been shown to be effective in learning vector field functions for 2D applications, and can be easily generalized to high dimensional data (Ma et al., 2013). For a function in the RKHS space, i.e., f∈H, The function can be evaluated at any point in X, as a summation of Gaussian kernels centered on the so-called “control points”:

$$f(x)=\sum_{j=1}^{m}\text{Γ}\left(x,\;{\tilde{x}}_{j}\right)c_{j},$$

where *m* is the number of control points and $\tilde{x}$ is the coordinate of the control point. *c*’s are coefficient vectors in ${\mathbb{R}}^{d}$, where *d* is the dimension of the vector field. The reproducing kernel is chosen to be a Gaussian function:

$$\text{Γ}(x,\tilde{x})=\exp\left(-w{\left(x-\tilde{x}\right)}^{2}\right),$$

where *w* is a width parameter. In addition, a norm of functions can be computed on H (Ma et al., 2013):

$$∥f{∥}_{H}^{2}=\sum_{i,j=1}^{m}c_{i}^{⊤}\text{Γ}\left({\tilde{x}}_{i},{\tilde{x}}_{j}\right)c_{j}.$$

In this representation, the loss function can be optimized with respect to the coefficient vectors *c*, and a vector-valued *L*_2_ regularization term can be introduced to it:

$$\text{Φ}(f)=\sum_{i=1}^{n}p_{i}{\left\|v_{i}-f\left(x_{i}\right)\right\|}^{2}+\lambda ∥f{∥}_{H}^{2},$$

$$\Rightarrow \text{Φ}\left(c_{1},c_{2},\ldots \right)=\sum_{i=1}^{n}p_{i}{\left\|v_{i}-\sum_{j=1}^{m}\text{Γ}\left(x_{i},{\tilde{x}}_{j}\right)c_{j}\right\|}^{2}+\lambda \sum_{i,j=1}^{m}c_{i}^{⊤}\text{Γ}\left({\tilde{x}}_{i},{\tilde{x}}_{j}\right)c_{j},$$

where *λ* is the regularization coefficient. The sparseVFC (sparse vector field consensus) algorithm (Ma et al., 2013) improves this loss function for better outlier identification and rejection by formulating the weight *p*_*i*_ as a likelihood function (see details in ***Outlier detection in vector field reconstruction***). The final loss function has an additional parameter *σ* accounting for inlier noise:

$$\text{Φ}(f)=\frac{1}{2\sigma^{2}}\sum_{i=1}^{n}p_{i}{\left\|v_{i}-f\left(x_{i}\right)\right\|}^{2}+\frac{\lambda }{2}∥f{∥}_{H}^{2}.$$

Let ***C*** = [***c***_1_ ***c***_2_ ··· ***c***_*m*_]^T^, and it can be shown that the solution C* to the following linear equation contains the coefficient vectors for the optimal vector field function f*:

$$\left(U^{⊤}PU+\lambda \sigma^{2}K\right)C=U^{⊤}PV,$$

where ***U*** is an *m*-by-*m* matrix whose elements are $\text{Γ}\left(x_{i},{\tilde{x}}_{j}\right)$, and ***K*** an *m*-by-*m* Gram matrix consisting of $\text{Γ}\left({\tilde{x}}_{i},{\tilde{x}}_{j}\right)$. The ***P*** matrix is a diagonal matrix of the weights *p*_*i*_, and ***V***=[***v***_1_, ***v***_2_, …, ***v***_*n*_].

The sparseVFC algorithm (Ma et al., 2013) consists of 1) an **E-step**: calculation of the diagonal matrix ***P*** based on the likelihood function for outlier rejection, and 2) an **M-step**: Solving the above linear system for ***C***, and updating the vector field function evaluations at sample points ***f***(***x***) with the optimal ***c***_*i*_’s. Other parameters, for example *σ*, are also updated accordingly in this step. The algorithm finishes when the loss function converges, or the number of optimization steps surpasses the designated maximum iterations.

###### Outlier detection in vector field reconstruction

Outlier detection is vital for robust vector field reconstruction from noisy RNA velocity data. The sparseVFC algorithm (Ma et al., 2013) models noise in velocities ***v*** of inliers with a Gaussian distribution, i.e.:

$$P(v|z=1,x,\theta )=\frac{1}{{\left(2\pi \sigma^{2}\right)}^{d/2}}\exp\left[-\frac{∥v-f(x){∥}^{2}}{2\sigma^{2}}\right],$$

where *z* is an indicator variable, such that *z* = 1 when the cell is an inlier, and *z* = 0 otherwise. ***θ*** contains all parameters, including the variance of the Gaussian distribution *σ*^2^, the vector field ***f***, and the prior probability *q* mentioned below. The probability distribution of outliers is modeled with a uniform distribution:

$$P(v|z=0,x,\theta )=\frac{1}{a},$$

where *a* is the volume of the domain for velocity vectors. Empirically, this is a parameter used for adjusting the aggressiveness of the outlier detection. Denote the fraction of inliers as *q*:

q=P(z=1∣x,θ).

Then, this is essentially a mixture model where the likelihood is:

$$P(v|x,\theta )=qP(v|z=1,x,\theta )+(1-q)P(v|z=0,x,\theta )\;\;=\frac{q}{{\left(2\pi \sigma^{2}\right)}^{d/2}}\exp\left[-\frac{∥v-f(x){∥}^{2}}{2\sigma^{2}}\right]+\frac{1-q}{a},$$

and the posterior probability can be derived from Bayes’ theorem (notice that the following corrects an error in (Ma et al., 2013)):

$$P(z=1|v,x,\theta )=\frac{qP(v|z=1,x,\;\;\theta )}{P(v|x,\;\;\theta )}\;\;=\frac{\exp\left[-\frac{∥v-f(x){∥}^{2}}{2\sigma^{2}}\right]}{\exp\left[-\frac{∥v-f(x){∥}^{2}}{2\sigma^{2}}\right]+\frac{1-q}{q}\frac{{\left(2\pi \sigma^{2}\right)}^{d/2}}{a}}.$$

For *n* such independent and identically distributed (i.i.d.) RNA velocity data samples, one can construct a diagonal matrix ***P*** = diag(*p*_1_, *p*_2_, · · ·, *p*_*n*_), where *p*_*i*_ = *P*(*z*_*i*_ = 1|***v***_*i*_, ***x***_*i*_, *θ*). The E-step of the EM algorithm evaluates this matrix, which is used as the weight in the loss function for sparseVFC.

To update *σ* and *q* at the M-step of each EM iteration following standard EM algorithm procedure, the updated parameters are the solutions of the following optimization problem (Ma et al., 2013):

$$\theta^{\text{new}}=\underset{\theta }{\arg\max}Q\left(\theta ,\theta^{\text{old}}\right),$$

where *Q*(***θ***, ***θ***^old^) is a conditional expectation of the complete-data log-likelihood function:

$$Q\left(\theta ,\theta^{\text{old}}\right)=\sum_{z}P\left(z|V,X,\theta^{\text{old}}\right)\ln P(V,z|X,\theta ),$$

$$P\left(z|V,X,\;\;\theta^{\text{old}}\right)=\prod_{i=1}^{n}P\left(z_{i}|v_{\text{i}},\;\;x_{i},\;\;\theta^{\text{old}}\right),$$

$$P(V,z|X,\theta )=\prod_{i=1}^{n}P\left(v_{i},z_{i}|x_{i},\theta \right).$$

With i.i.d. samples, one can show that (Ma et al., 2013):

$$Q\left(\theta ,\theta^{\text{old}}\right)=\sum_{i=1}^{n}\sum_{z_{i}=0}^{1}P\left(z_{i}|v_{i},x_{i},\theta^{\text{old}}\right)\ln P\left(v_{i},z_{i}|x_{i},\theta \right)\;\;=\sum_{i=1}^{n}\sum_{z_{i}=0}^{1}P\left(z_{i}|v_{i},x_{i},\theta^{\text{old}}\right)\ln\left(P\left(v_{i}|z_{i},x_{i},\theta \right)P\left(z_{i}|x_{i},\theta \right)\right)\;\;=\sum_{i=1}^{n}\left\{p_{i}\left(\ln q-\frac{d}{2}\ln\left(2\pi \sigma^{2}\right)-\frac{{\left\|v_{i}-f\left(x_{i}\right)\right\|}^{2}}{2\sigma^{2}}\right)+\left(1-p_{i}\right)\ln\frac{1-q}{a}\right\}.$$

By taking derivatives of *Q*(***θ***, ***θ***^old^) w.r.t. *σ*^2^ and *q* and equating them to zero, one obtains the solutions for updating the parameters:

$$\sigma^{2}=\frac{{\left(V-F\right)}^{⊤}P(V-F)}{d\times trP},$$

γ=trP/n,

where ***F*** = [***f***(***x***_1_), ***f***(***x***_2_), …, ***f***(***x***_*n*_)]^⏉^.

###### Effects of parameters in vector field reconstruction

The sparseVFC algorithm with an isotropic Gaussian kernel has four main parameters: the number of control points *m*, the regularization parameter *λ*, the inverse bandwidth of the Gaussian kernel *w*, and the maximal number of iterations *N*_*max*_. Their default values and effects of changes in these values on the resultant vector field are summarized in the following table:

**Table:** 

	Default	Effects
*m*	5% of the number of cells, with a mini- mum of 50 control points	Too small: the approximation of the vector field in RKHS is too sparse (underfitting); Too large: the optimization of the loss function is memory- and time-consuming.
*λ*	3	Too small: overfitting; Too large: underfitting.
*w*	determined by the distribution of the data (see **below**)	Too small: large bandwidth means all control points have approximately equal contributions to all surrounding states in the vector field, and the vector field becomes linear; Too large: small bandwidth means control points have insufficient influence over distant states, result- ing in zero velocities evaluated for distant cells.
*N_max_*	500	Too small: the algorithm is terminated before reasonable convergence (underfitting); Too large: when convergence is hard to achieve, the algorithm takes too long with negligible improve- ments in minimizing the loss function.

The inverse bandwidth *w* is determined in the following way:

1. Find *k*-nearest-neighbors for each cell (by default 20% of the number of cells);
2. Compute the mean distance of each cell to its neighbors *d*_*m*_;
3. The inverse bandwidth $w=1.5/\left(\sqrt{2}d_{m}\right)$, so that the standard deviation of the Gaussian kernel is *σ* = *d*_*m*_/1.5.

##### Dynamo: 3) Vector field topological and differential geometry analyses

###### Topological analysis of single-cell vector field

In this study, we focus on calculating fixed points and nullclines in our topological analysis of vector fields. The fixed points are defined as points where the value of the vector field function is zero:

f(x)=0,

and the solution can be obtained using any nonlinear equation solver (*SciPy* fsolve is used in our case). Because the solver can only find fixed points closest to an initial guess ***x***_0_, we simply randomize *n* such initial points in a domain containing all data points. We used Latin hypercube sampling technique (Iman et al., 1981) to sample initial points effectively. To characterize the stability of a fixed point, the Jacobian is evaluated at the point, and we simply categorize fixed points into three types based on the signs of its Jacobian’s eigenvalues:

1. Stable fixed point (attractor): all eigenvalues are negative;
2. Unstable fixed point (repulsor): all eigenvalues are positive;
3. Saddle point: The eigenvalues are a mixture of positive, negative values, or even zeros.

If one is interested in fixed points of a specific order (i.e., with a given number of positive eigenvalues), one may use a quasi-Newton conditional root-finding algorithm developed by Wang et al. (Wang et al., 2014).

Nullclines are lines (in 2D) or surfaces (in higher dimensions) when at least one component of the vector field is zero. For example, for a 2D vector field ***f***(*x, y*) = [*f*_*x*_(*x*, *y*) *f*_*y*_(*x*, *y*)]^⏉^, the *x*-nullcline consists of points where:

$$f_{x}(x,y)=0.$$

Because it is computationally expensive to compute nullclines in higher dimensions, in our study we limit the calculation to 2D vector fields. In the 2D case, fixed points are intersections of *x*- and *y*-nullclines, so we compute nullclines using a pseudo-arclength continuation method (Seydel, 1988) starting at a certain fixed point. As an example, to find the next point ***p***_1_ on the *x*-nullcline, given a known point ***p***_0_ and a tangent vector of the nullcline ***v***_0_, one simply finds the initial guess for ***p***_1_ by:

$$p_{1}^{*}=p_{0}+\varepsilon v_{0},$$

where *ε* is an incremental increase in the arclength. ***p***_1_ can then be found by numerically solving the following equations:

$$\{\begin{array}{l} f_{x}\left(p_{1}\right)=0,\\ \left(p_{1}-p_{0}\right)\cdot v_{0}-\varepsilon =0. \end{array}$$

This guarantees that the solution ***p***_1_ is *ε* away from the known point ***p***_0_ on the nullcline. The tangent vector for the next iteration is approximated as ***v***_1_=(***p***_1_ − ***p***_0_)/|***p***_1_ − ***p***_0_|, and the first tangent vector at the fixed point is a normalized random vector.

##### Stable limit cycle detection and redundant trajectory removal for numerical integration of vector fields

Stable limit cycles cause redundant sampling for trajectories integrated using vector fields. In this study, we focus on detecting limit cycles for a 2D vector field, but the method can be easily generalized to higher dimensions. Suppose we have a trajectory of *n* points ${\left\{\left(x_{i},y_{i}\right)\right\}}_{i=1}^{n}$, and we divide it into *k* (*k* = 4 by default) intervals, each of which contains *m* points $\left\{X_{j},Y_{j}\right\}={\left\{\left(x_{i}^{j},y_{i}^{j}\right)\right\}}_{i=1}^{m}$, where *j* = 1, 2, …, *k*. If a portion of the trajectory enters a stable limit cycle and orbits around the corresponding fixed point, the *x*- and *y*-coordinates of the points are periodic. We use the fast Fourier transform to obtain the frequency spectra for the *x*- and *y*- coordinates of points in the last two intervals:

$$f_{x}^{j}=\mathrm{FFT}\left(X_{j}\right),\;f_{y}^{j}=\text{FFT}\left(Y_{j}\right)$$

where *j* = *k* − 1, *k*. Let $f^{j}=\left[f_{x}^{j},f_{y}^{j}\right]$ be the concatenated frequency spectrum. If the spectral difference 1m‖fk−1−fk‖ is smaller than a certain threshold (0.05 by default), the last interval is considered redundant and thus removed. This procedure is performed iteratively, until the redundancy criterion is not met.

##### Confidence of identified fixed points

We notice that some identified fixed points are far away from regions populated with data points, where the reconstructed vector field may be less reliable. We quantify the confidence of the fixed points based on how far they are from domains populated with cells, and use the filled color of each node (corresponds to the fixed points) to represent the confidence of those fixed points when creating the topography plot in ***dynamo***.

##### Prediction of cell fate via integration of vector field given initial cell states, and fate probability estimation

Once the vector field was learned, either in reduced UMAP space, top PCA space or even the original gene expression space, we can use it to predict the historical and future cell expression states over arbitrary time scales given any initial cell state. This can be achieved by integrating the continuous vector field from one or a set of initial cell states forward or backward in time. When the integration was performed for the early cells of a particular clone, the integration paths can be used to calculate the minimal distances from clone cells at later time points to the paths, as well as the fate bias (see below), to validate the accuracy and single cell trajectory predictability of the reconstructed vector field, as demonstrated in the HL60 or the hematopoiesis clone tracing datasets analyses (Figure S5).

Fate probability is currently calculated as the percentage of points along the predicted cell fate trajectory whose nearest observed cells belong to a particular cell group, e.g., cell type. The distances to the nearest cells are required to be small, and are determined by the median distance of cells and a distance threshold, see details below:

Cell fate trajectories predicted by our vector field method sometimes end up in regions where few or no cells were actually measured (see the above section). A heuristic method is thus used to iteratively move backwards along the integrated trajectory to assign cell fate. We first identify the region with small velocity in the tail of the trajectory, which is determined by a threshold of speed, and then check whether the points in the region are close enough to the observed data points (cells), determined by a distance threshold. If not, we select another set of points upstream along the trajectory by one time step while keeping the same amount of points. This moving-back procedure stops when all the selected points of the trajectory are sufficiently close to the observed cells.

In case that the trajectory is close to a few random cells, we find the second nearest cells for selected points on the trajectory to include more cells as the nearest neighbors, especially those from terminal cell types. We then use group information of those observed cells to define the fate probability. Fate probability for a particular cell group is then defined as:

1−(sum(distances>distancethreshold×mediandistance)+movebacksteps)/(#selectedpoints+movebacksteps).

“distances” are distances between the selected points of the trajectory and the second nearest cells of a par- ticular cell group. “median distance” is the median nearest-neighbor distance of all cells. “move back steps” is the number of steps moving backwards along the trajectory. Note when moving backward, the selected points do not necessarily have small velocity anymore.

##### Animating the single-cell trajectories on 2D vector field

Animating cell fate commitments relies on the numerical integration of vector fields, as in the above section. Note that this two-dimensional space can be either UMAP, any two dimensions from PCA, or any two genes of interest, as long as we first reconstruct the vector field on this two-dimensional space. A vector field animation visualizes the movement of a set of cells in gene expression space, and the long-range trajectory predicted by the reconstructed vector field. Thus, it provides intuitive visual understandings of the RNA velocity, curvature, acceleration, and cell fate commitment in action.

##### Differential geometry analysis of the reconstructed single-cell vector field

We derive the analytical formula of Jacobian of the vector field which improves the computational efficiency tremendously compared to numerical approaches. The vector field function obtained from the sparseVFC algorithm has the following form (See Box 2 for details):

$$f(x)=\sum_{i=1}^{m}\text{Γ}\left(x,{\tilde{x}}_{i}\right)c_{i},$$

where Γ is the Gaussian kernel, $\tilde{x}$ are the control points, and ***c*** are the combination coefficient vectors. Because the vector field is a linear combination of Gaussian kernels, whose derivative is:

$$\frac{\partial \text{Γ}(x,\tilde{x})}{\partial x}=-2w\;\;\exp\left(-w{\left(x-\tilde{x}\right)}^{2}\right)(x-\tilde{x})=-2w\text{Γ}(x,\tilde{x})(x-\tilde{x}).$$

The Jacobian of the vector field function is then:

$$J=\frac{\partial f(x)}{\partial x}=-2w\sum_{j=1}^{m}\text{Γ}\left(x,{\tilde{x}}_{j}\right)c_{j}{\left(x-{\tilde{x}}_{j}\right)}^{⊤}.$$

Let:

$$K=diag\left[\text{Γ}\left(x,{\tilde{x}}_{1}\right),\text{Γ}\left(x,{\tilde{x}}_{2}\right),\;\ldots ,\;\text{Γ}\left(x,{\tilde{x}}_{m}\right)\right],$$

$$C={\left[\begin{array}{c} c_{1},\;\;\;\;c_{2},\;\;\;\;\ldots ,\;\;\;\;c_{m} \end{array}\right]}^{⊤},$$

$$D={\left[x-{\tilde{x}}_{1},\;x-{\tilde{x}}_{2},\;\ldots ,\;x-{\tilde{x}}_{m}\right]}^{⊤}.$$

The above analytical form of the Jacobian can be vectorized into:

$$J=-2wC^{⊤}KD.$$

The divergence is the trace of the Jacobian matrix:

∇⋅f=TrJ.

Note that it is possible to have a point where the vectors converge in one direction but diverge in another, a case that is not depicted in the diagram in Box 1. This means that although an attractor (repulsor) always has negative (positive) divergence, the opposite does not necessarily hold.

The curl is only computable in 2D or 3D, and is computed as follows for a 3D system:

$$\nabla \times f=\left[\begin{array}{c} \frac{\partial f_{z}}{\partial y}-\frac{\partial f_{y}}{\partial z}\\ \frac{\partial f_{x}}{\partial z}-\frac{\partial f_{z}}{\partial x}\\ \frac{\partial f_{y}}{\partial x}-\frac{\partial f_{x}}{\partial y} \end{array}\right].$$

Many differential geometry quantities are defined on *streamlines*, which are curves everywhere tangent to the vector field. The streamlines can be parametrized with time *t*, denoted ***x***(*t*), as they are essentially trajectories of cells moving in the vector field. The *acceleration* is the time derivative of the velocity:

$$a=\frac{\text{d}v}{\text{d}t}=\frac{\text{d}}{\text{d}t}f(x(t))=\sum_{i=1}^{d}\frac{\partial f}{\partial x_{i}}\frac{\partial x_{i}}{\partial t}=Jv,$$

where ***v*** is the velocity vector. The curvature vector of a curve is defined as the derivative of the unit tangent vector (d(v∥v∥)dt), divided by the length of the tangent (∥***v***∥):

$$\kappa =\frac{1}{∥v∥}\frac{\text{d}}{\text{d}t}\frac{v}{∥v∥}=\frac{Jv(v\cdot v)-v(v\cdot Jv)}{∥v{∥}^{4}}.$$

In the context of velocity vector fields and streamlines, the unit tangent vector is the normalized velocity. In 2D, the curvature formula has an equivalent but simpler form:

$$\kappa =\frac{v\times (Jv\times v)}{∥v{∥}^{4}},$$

Although acceleration and curvature are mathematically defined on streamlines, the actual calculation, as shown above, can be done pointwise using only the velocity and the Jacobian evaluated at the point of interest. Because the acceleration or the curvature can be calculated for any point in the state space, one obtains the acceleration or curvature vector field.

Because the vector field function is often learned in a PCA-reduced space, and to acquire gene-specific information, a transformation of the Jacobian from the PCA space to the original gene expression space is needed. Suppose the first *k* principal components form a *d*-by-*k* matrix ***Q***, where *d* is the dimension of the original gene expression space, then the gene-specific Jacobian ***G*** is:

$$G=QJQ^{⊤}.$$

Thus, the *ij*-th element of ***G*** is the partial derivative of the velocity of gene *i* with respect to the expression level of gene *j*. The obtained Jacobian *G* here is only an approximation of the true gene-specific Jacobian, as only *k* < *d* principal components are used.

##### Ranking genes based on differential geometrical quantities

Generally, given some quantity (expression, velocity, acceleration, curvature, etc.) calculated for each gene in each cell, i.e. a *n* × *m* matrix ***Q***, where *n* is the number of cells, and *m* the number of genes, one can obtain a gene-wise vector of such quantities by averaging over cells:

$$q_{j}=\langle Q_{\cdot ,j}\rangle =\frac{1}{n}\sum_{i=1}^{n}Q_{i,j}.$$

Suppose that cells are divided into several clusters, e.g., distinct cell types, the above average can be calculated for each cluster:

$$q_{j}^{c}={\langle Q_{.,j}\rangle }_{c}=\frac{1}{n_{c}}\sum_{i\in \;\;C}Q_{i,j},$$

where *C* is the set of cells in cluster *c*, and *n*_*c*_ the number of cells in *C*. When one is interested in the absolute values of the quantities, the average is calculated with |***Q***|. Then, genes can be ranked based on *q*^*c*^ for each cluster. For the ranking of the Jacobian, since each cell is associated with an *m* × *m* Jacobian matrix, the whole data is an *m* × *m* × *n* 3D matrix. The same averaging method is applied to all cells or each cluster:

$$\langle J_{\zeta ,\xi ,\cdot }\rangle =\frac{1}{n}\sum_{i=1}^{n}J_{\zeta ,\xi ,i},$$

$${\langle J_{\zeta ,\xi ,\cdot }\rangle }_{c}=\frac{1}{n_{c}}\sum_{i\in \;\;C}J_{\zeta ,\xi ,i}.$$

Because for each cell or cluster, the Jacobian or average Jacobian is an *m* × *m* matrix, and the ranking can be performed in various ways to identify putative interactions, regulators, and effectors:

1. Top interactions: because each element in the averaged Jacobian indicates the change in the velocity of the effector with respect to the change in the expression of the putative regulator, top elements suggest strong gene–gene interactions in each cell or cell type, as below.
2. Top regulators for each effector: we rank each row of the averaged Jacobian matrix, so that for each effector, one obtains the top genes that potentially regulate the effector.
3. Top effectors for each regulator: we rank each column of the averaged Jacobian matrix, so that for each regulator, one obtains top genes potentially regulated by the regulator.
4. Top regulators: For effector *ζ*, we sum up its averaged Jacobian elements with respect to all possible regulators:
$$R_{\zeta }=\sum_{\xi =1}^{n}\langle J_{\zeta ,\xi ,\cdot }\rangle =\sum_{\xi =1}^{n}\langle \frac{\partial f_{\zeta }}{\partial x_{\xi }}\rangle ,$$
  and rank all *R*_*ζ*_, which shows the top genes potentially involved in the regulation of others;
5. Top effectors: For regulator *ζ*, a summation is taken across all effectors:
$$E_{\xi }=\sum_{\zeta =1}^{n}\langle J_{\zeta ,\xi ,\cdot }\rangle =\sum_{\zeta =1}^{n}\langle \frac{\partial f_{\zeta }}{\partial x_{\xi }}\rangle .$$
  The ranking of all *E*_*ξ*_ reveals top regulated genes.

##### Estimating kinetic parameters by fitting the Jacobian vs. expression curve

Because the reconstructed vector field is expressed as a set of implicit basis functions, not explicitly as Hill functions, in the current framework we are not able to directly obtain kinetic parameters such as the Hill coefficient. Nevertheless, the reconstructed vector field encodes such information, and additional computations are applied to extract that information. We demonstrate this possibility on simplistic network motifs such as PU.1–GATA1, by fitting the derivatives of inhibitory or activation Hill equations to the corresponding Jacobian elements. Further efforts will be needed to make such efforts generally applicable to systems with more sophisticated mechanisms.

Formally, we assume that the activation effect of gene *x* on the target gene takes the form of an activating Hill function:

$$h_{a}(x)=\frac{x^{n}}{K^{n}+x^{n}},$$

and that the inhibition effect assumes the form of an inhibitory Hill function:

$$h_{i}(x)=\frac{K^{n}}{K^{n}+x^{n}}$$

For self-interactions, there is also an additional degradation term, so:

$$H_{a}(x)=h_{a}(x)-\gamma x,\;H_{i}(x)=h_{i}(x)-\gamma x.$$

Taking the derivatives:

$$\frac{\text{d}H_{a}(x)}{\text{d}x}=\frac{nK^{n}x^{n-1}}{{\left(K^{n}+x^{n}\right)}^{2}}-\gamma ,\;\frac{\text{d}H_{i}(x)}{\text{d}x}=-\frac{nK^{n}x^{n-1}}{{\left(K^{n}+x^{n}\right)}^{2}}-\gamma .$$

In Figure S6H, I, the means and standard deviations of the Jacobian vs. expression profiles were calculated and fitted with the above derivatives using the *SciPy* curve_fit function (Virtanen et al., 2020). For cross- interactions (GATA1 to SPI1 and SPI1 to GATA1), the degradation constant *γ* is fixed to zero.

##### Three types of regulatory interaction analyses

Three increasingly explicit regulatory interaction analyses are possible for the continuous vector field, namely: 1) cell-wise analyses; 2) trajectory-wise analyses; and 3) plane-wise analyses. The cell-wise analyses cor- respond to regular analyses across measured cells, whereas the trajectory-wise and plane-wise analyses are unique to generative vector fields learned with ***dynamo***. Trajectory-wise analyses reveal trajectory- dependent acceleration, curvature, interactions, etc., where the trajectory can be either the vector field integration path (Figure S6J) or the predicted least action path (Figure 6C). Because integration paths, or least action paths are predicted from the vector fields, data points along the paths are often not observed but predicted states. Plane-wise analyses reveal “direct” interactions for any characteristic cell states (such as the GMP-like state in Figure S6K) by varying genes of interest while holding all other genes constant. It must be noted that with scRNA-seq data alone, one cannot exclude indirect interaction influences resulting from post-transcriptional regulation or from other hidden variable effects.

##### Vector field simulation and benchmark of the two-gene bifurcation system

We use the simple canonical self-activating and mutual-inhibiting two-gene motif that frequently appears in a variety of cell fate bifurcation systems to introduce key concepts in dynamical systems and differential geometry employed in this study (Figure 1A). The vector field function of this system is adapted from Qiu et al. (Qiu et al., 2012):

$$f_{1}\left(x_{1},x_{2}\right)={\dot{x}}_{1}=a_{1}\frac{x_{1}^{n}}{K_{1}^{n}+x_{1}^{n}}+b_{1}\frac{K_{1}^{n}}{K_{1}^{n}+x_{2}^{n}},$$

$$f_{2}\left(x_{1},x_{2}\right)={\dot{x}}_{2}=a_{2}\frac{x_{2}^{n}}{K_{2}^{n}+x_{2}^{n}}+b_{2}\frac{K_{2}^{n}}{K_{2}^{n}+x_{1}^{n}},$$

where *K*_1_ = *K*_2_ = *a*_1_ = *a*_2_ = *b*_1_ = *b*_2_ = 1, and *n* = 4. In the following two subsections, we will describe how the demonstration of the vector field analysis and the benchmarking of our vector field reconstruction with this two-gene system are performed.

##### Mapping the topological and geometry feature of the two-gene system

To make the quiver plot of the two-gene system, we first set the expression range of *x*_1_ and *x*_2_ to [0, 2.5] and plot the velocity values calculated with the above vector field function on a 25-by-25 grid with even spacing in this space. The velocity values on the grid are also used to create the streamline plot. Individual trajectories associated with states 1, 2, 3 are obtained via numerical integration of the vector field function. Fixed points are solved analytically from the vector field function. To obtain the separatrices, we integrate the vector field function backwards in time, starting from initial points that are close to saddle points in both directions of eigenvectors whose eigenvalues are negative. The Jacobian of this system is a 2-by-2 matrix:

$$J=\left[\begin{array}{cc} \frac{\partial f_{1}}{\partial x_{1}} & \frac{\partial f_{1}}{\partial x_{2}}\\ \frac{\partial f_{2}}{\partial x_{1}} & \frac{\partial f_{2}}{\partial x_{2}} \end{array}\right],$$

where:

$$\frac{\partial f_{1}}{\partial x_{1}}=a_{1}\frac{nK_{1}^{n}x_{1}^{n-1}}{{\left(K_{1}^{n}+x_{1}^{n}\right)}^{2}}-1,$$

$$\frac{\partial f_{2}}{\partial x_{1}}=-b_{2}\frac{nK_{2}^{n}x_{1}^{n-1}}{{\left(K_{2}^{n}+x_{1}^{n}\right)}^{2}},$$

and the rest of the elements can be deduced easily from the above results due to the symmetry of the system. With the Jacobian, we can also obtain the acceleration, curvature, divergence, and curl. The heatmaps for the four elements of Jacobian, divergence, and curl are superimposed with the quiver or streamline plot. For vectors like acceleration and curvature, we plot their magnitudes together with the corresponding vector fields. To enhance the presentation of the plots for the above differential geometry quantities, finer grids, 2-D gaussian kernel smoothing, and different colormaps are used as needed.

##### Benchmarking the reconstruction of the vector field and the calculation of differential geometry quantities

To generate the benchmark dataset, we randomly select 5000 points within the same domain used in the above plots and calculate the corresponding velocity vectors for those points. Those cell states and velocity vector pairs are then used as inputs to reconstruct vector field function with ***dynamo*** using default parameters. Attractor, saddle points, and nullclines are estimated with the reconstructed vector field function and plot with the streamline plot that is also based on the reconstructed vector field function via ***dynamo***. We used the reconstructed vector field function to calculate analytical Jacobian, acceleration, curvature, curl, and divergence using ***dynamo***. Scatterplots from ***dynamo***, including a frontier showing the boundary of all those cells, are used to plot the 5000 sampled cells, colored according to either the four elements of the Jacobian, divergence, or curl at those points. ***Dynamo*** is also used to estimate the acceleration and curvature for those sampled cells, and then plot their magnitudes together with the corresponding vector fields (i.e. acceleration or curvature vector field). We calculate the analytical Jacobian, acceleration, curvature, divergence, and curl with the true vector field function at those sampled data points and compare the corresponding values estimated from ***dynamo*** with scatterplots (Figure 4C–E).

To demonstrate the efficiency of our differential geometry analyses with the reconstructed vector field function, we compare the time used either by the numeral approaches that build upon the *numdifftools* or by the analytical approaches, both implemented in ***dynamo***. Note that numerical approaches for those differential geometry quantities are only possible with the analytical vector field function we learned, especially in the high-dimensional gene expression space.

##### Robustness of vector field reconstruction and differential geometry analyses to cell downsampling and noise

We downsampled the cells and injected different levels of noise into the simulated dataset in Figure S4I, J to benchmark the robustness of vector field reconstruction and differential geometry analysis. For bench- marking of robustness to cell downsampling (Figure S4I), the number of simulated cells was progressively downsampled from 5,000 to 156 cells with five repeats at each downsampling depth, followed by re-performing the vector field reconstruction and re-computing differential geometry quantities. We then calculated the cosine or Spearman’s correlation and RMSE between the predicted vector field quantities (such as velocity, Jacobian, pseudotime, etc.) and the corresponding ground-truth quantities at each downsampling depth and repeat. Finally, we plotted the cosine or Spearman’s correlation or RMSE as a function of sampling depth while including corresponding linear regression fitting curves (Figure S4I). For benchmarking of robustness to noise (Figure S4J), random Gaussian noise was injected to the velocities data before reconstruction of vector fields and computation of differential geometry quantities. The injected noise had a mean of zero, and standard deviation (s.d.) progressively increased to the same level (100%) as the magnitude of mean velocity. Similar to what was done for the cell downsampling benchmark, we plotted the cosine or Spearman’s corre- lation or RMSE as a function of noise level while including the corresponding linear regression fitting curves (Figure S4J). All parameters used by the algorithm, except beta, ecr, and sigma (the algorithm is invariant to parameters, ecr and sigma, while beta is hard to set), were varied to check against the performance of vector field reconstruction and differential geometry analyses, similar to benchmarking of cell downsampling and noise level, to demonstrate the robustness of the algorithm (Figure S4K).

#### Dynamo: 4) Vector field predictions with LAP and *in silico* perturbations

##### Toward ​​*à la carte* reprogramming: a least action path approach

The least action path (LAP) principle has previously been used to predict the optimal transition path of cell fate transition for simple and designed systems (Qiu et al., 2012; Wang et al., 2011, 2014). Because ***dynamo*** learns analytical vector field functions, we reason that we are able to move beyond contrived systems to real biological systems with scRNA-seq datasets. Formally, the LAP method, based on a numerical algorithm adapted from (Perez-Carrasco et al., 2016), aims to find an optimal path between the given starting and end points ***x***_0_ and ***x***_*n*_. In the context of cell state transitions, these points may correspond to different cell types, such as the HSC and the Meg lineage attractor states in Figure 6C. The path is discretized as a sequence of points *P* = {***x***_0_, ***x***_1_, …, ***x***_*n*_}, which forms *n* line segments (Figure 6C). For each line segment, the discrete tangential velocity can be calculated as ***v***_*k*_ = (***x***_*k*_ − ***x***_*k* − 1_)/Δ*t*, where Δ*t* is the time step for the cell to move from ***x***_*k* − 1_. In addition to the deterministic vector field, we also assume a certain degree of stochasticity in the system:

$$\dot{x}=f(x)+\sigma \eta (t),$$

where ***η***(*t*) is a stochastic white noise and *σ* the size of it. The action *S* along the discrete path is defined as (Perez-Carrasco et al., 2016):

$$S(P,\text{Δ}t)=\frac{1}{2D}\sum_{k=1}^{n}{\left(v_{k}-f\left(y_{k}\right)\right)}^{2}\text{Δ}t,$$

where ***y***_*k*_ are the middle points of the line segments, i.e., ***y***_*k*_ *=* (***x***_*k* − 1_ + ***x***_*k*_)/2. We have also assumed the diffusion matrix to be a constant *D*, such that *D* = *σ*^2^/2. It is intuitive that a path whose tangential velocities ***v*** align with the vector field has smaller action than paths that do not. The LAP is a path such that:

$$P^{*}=\underset{P,\text{Δ}t}{argmin}S(P,\text{Δ}t)=\underset{P,\text{Δ}t}{argmin}\frac{1}{2D}\sum_{k=1}^{n}{\left(v_{k}-f\left(y_{k}\right)\right)}^{2}\text{Δ}t.$$

The algorithm for finding the LAP therefore consists of two steps:

1. Minimization of the action by varying the time step. The optimal time step Δt* given a fixed path *P* is a simple univariate least square minimization, i.e.:
$$\text{Δ}t^{*}=\underset{\text{Δ}t}{argmin}\frac{1}{2D}\sum_{k=1}^{n}{\left(\frac{x_{k}-x_{k-1}}{\text{Δ}t}-f\left(y_{k}\right)\right)}^{2}\text{Δ}t.$$
2. Minimization of the action by varying the path without moving the starting and end points. The optimal path P* given a fixed time step Δ*t* is found by:
$$P^{*}=\underset{\left\{x_{1},x_{2},\ldots ,x_{n-1}\right\}}{argmin}\frac{1}{2D}\sum_{k=1}^{n}{\left(\frac{x_{k}-x_{k-1}}{\text{Δ}t}-f\left(\frac{x_{k-1}+x_{k}}{2}\right)\right)}^{2}\text{Δ}t.$$
  For a *d*-dimensional vector field, the number of variables in the above optimization problem is *d* × *n*. To mitigate the computational cost, the Jacobian of the action w.r.t. the path (more specifically, the *a*-th component of the *k*-th point) is analytically computed:
$$\frac{\partial S}{\partial x_{k}^{a}}=\frac{1}{D}\left(v_{k}^{a}-v_{k+1}^{a}+f^{a}\left(y_{k+1}\right)-f^{a}\left(y_{k}\right)\right)\;\;-\frac{1}{2D}\left({\left(v_{k+1}-f\left(x_{k+1}\right)\right)\cdot \frac{\partial f}{\partial x^{a}}|}_{x_{k+1}}+{\left(v_{k}-f\left(x_{k}\right)\right)\cdot \frac{\partial f}{\partial x^{a}}|}_{x_{k}}\right).$$
  Note that the partial derivative of the vector field ∂***f***/∂*x*^*a*^ is the *a*-th row of the Jacobian of the vector field mentioned in the section “differential geometry analysis of the reconstructed single-cell vector field”. With the analytical Jacobian, the computation efficiency of the LAP optimization improves tremendously, making the LAP calculation feasible to operate in high-dimensional space, such as the top 30 PCs.

The LAP is found by iterating between the two steps, and empirically we found that the path converges in two or three iterations. By default, the LAP optimization is initialized with the interpolated shortest path on the *k*NN graph of cells.

For rare transitions with $S_{T^{*}}\gg 0$ (e.g., dedifferentiation and transdifferentiation), the transition rate (number of transitions per unit time) is proportional to the exponential of actions of all paths. The Freidlin–Wentzell theorem dictates that the LAP with the minimal traversal time (which will be referred to as the optimal path below) contributes the most to this transition rate (Aurell and Sneppen, 2002; Freidlin and Wentzell, 2012; Maier and Stein, 1997; Onsager and Machlup, 1953):

$$R(A\to B)\approx C\exp\left(-S_{T^{*}}\right),$$

where *A* and *B* are two cell types, $S_{T^{*}}$ the action of the optimal path, and *C* a proportionality constant. Furthermore, the transition time, or more specifically the mean first passage time (MFPT), is related to the transition rate:

$$\text{MFPT}=\frac{1}{R(A\to B)}.$$

Therefore, the action of the optimal path predicts both the likelihood and transition time for such rare transitions. Again, most reprogramming experiments take a few weeks or months, depending on the exact initial and terminal cell states (Takahashi and Yamanaka, 2006)

For natural transitions, there are many paths following the vector field streamlines, which have nearly zero actions. The LAP is not unique, and the algorithm often ends up finding one with non-optimal traversal time. Therefore, after the algorithm converges to a LAP with traversal time T*, we perform an additional linear search with respect to the traversal time *T* < T*, and find the LAP whose traversal time is at the elbow of the action vs. traversal time curve. To determine the elbow *T*^*e*^, we interpolate the normalized action vs. traversal time curve using the cubic spline. Then we calculate the second derivative of the interpolated curve numerically, and find the traversal time that is closest to T* and exceeds the numerical threshold. The resulting LAP is the fastest LAP (F-LAP).

In order to identify key drivers of cell fate transition, after transforming the path from the PCA space to the gene space, we calculate the mean square displacement (MSD) for every gene *i* along the path:

$${\text{MSD}}_{i}=\sum_{t=0}^{T}{\left(y_{i}(t)-y_{i}(0)\right)}^{2},$$

and rank the genes based on it. Arguably, those top genes can be prioritized as TF cocktails for optimal reprogramming between any cell types, paving the road for *à la carte* reprogramming (Graf and Enver, 2009) for regenerative medicine.

##### Hematopoietic optimal transition matrix with the LAP method and prioritization of transcription factors

We used the LAP method to predict an optimal transition matrix between all stable hematopoietic cell types. First, we identified the cell states in either UMAP or PCA space closest to the identified fixed points, and treated them as the stereotypical states for hematopoietic cell types. We then looped through all possible transitions (5 × 6 = 30) from one stereotypical cell state to another and performed LAP analyses. Optimizations of LAPs for all transitions were done in either UMAP or PCA space with default parameters and settings, except that when searching for developmental LAPs (LAPs starting from HSCs state to other mature cell types), we explicitly used the F-LAPs, as recommended in ***Vector field predictions with LAP and in silico perturbations***. We used the LAPs calculated in UMAP space to visualize the developmental, dedifferentiation, and transdifferentiation LAPs in Figure 6D, Figure S7A, B, respectively. We created the barplot of LAP time (the traversal time) for developmental LAPs (Figure 6E) and heatmap of actions for all transitions (Figure 6F), both calculated with the LAPs computed in PCA space. With a LAP computed in PCA space, we can project it back to the original gene expression space to obtain the full transcriptomic kinetics. We showcased the transcriptomic kinetics along LAPs from HSC to the Bas lineage and vice versa as kinetic heatmaps (Figure 6G, S7C). We ranked all transcription factors (133 in total) based on their cumulative MSD (in a descending manner) and plotted the expression kinetics of the top three TFs for each transition along the LAP as a function of the LAP transition time (units: hours) (Figure S7D).

Based on the ranking (*R*) of each TF for each transition, we then defined a priority score *S*:

$$S=1-\frac{R}{\text{\#TF}},$$

where #TF corresponds to the total number of TFs for each transition. When a gene’s cumulative MSD is high, indicating a larger contribution to the transition, the rank is small and thus the priority score is close to 1. To the best of our ability, we manually compiled a complete table of known hematopoietic cell fate transitions (including developmental process) and the key TFs corresponding to each transition (Supplementary Table 2). To globally quantify the accuracy of our LAP method in prioritizing TFs of cell fate transitions, we used the roc_curve function from *sklearn* (Pedregosa et al., 2011) to perform a universal ROC (receiver operating characteristic) curve analysis using priority scores from all transitions. Specifically, we gradually relaxed the priority score and calculated the average rate of true positives (*y*-axis) and that (*x*-axis) of false positives (TFs not on the compiled list) across all transitions, eventually creating the universal ROC curve across all cell transitions. We also calculate the AUC (area under the curve) of the ROC curve. The ROC analysis and AUC of LAP TF prioritization are presented in Figure 6H.

##### *In silico* perturbation to predict gene-wise perturbation effects and cell fate diversions

We leverage the analytical Jacobian of the reconstructed vector field function to make *in silico* genetic perturbation and predict cell-fate outcomes after the perturbation. Mathematically, for gene in any cell, the genetic perturbation effects or change in its velocity (or more accurately, the vector field) w.r.t. to small perturbations in the expression of all genes in the network (encoded by the Jacobian matrix ***J***), $\text{d}x_{1},\text{d}x_{2,\ldots ,}\text{d}x_{n}$, can be calculated with the *exact differential*:

$$\text{d}f_{i}=\frac{\partial f_{i}}{\partial x_{1}}\text{d}x_{1}+\frac{\partial f_{i}}{\partial x_{2}}\text{d}x_{2}+\ldots +\frac{\partial f_{i}}{\partial x_{n}}\text{d}x_{n}.$$

In vectorized form:

$$\left[\begin{array}{c} \text{d}f_{1}\\ \text{d}f_{2}\\ \ldots \\ \text{d}f_{n} \end{array}\right]=\left[\begin{array}{rrrr} \frac{\partial f_{1}}{\partial x_{1}} & \frac{\partial f_{1}}{\partial x_{2}} & \cdots & \frac{\partial f_{1}}{\partial x_{n}}\\ \frac{\partial f_{2}}{\partial x_{1}} & \frac{\partial f_{2}}{\partial x_{2}} & \cdots & \frac{\partial f_{2}}{\partial x_{n}}\\ \ldots & \cdots & \cdots & \cdots \\ \frac{\partial f_{n}}{\partial x_{1}} & \frac{\partial f_{n}}{\partial x_{2}} & \cdots & \frac{dx_{1}}{\partial x_{n}} \end{array}\right]\left[\begin{array}{c} \text{d}x_{1}\\ \text{d}x_{2}\\ \ldots \\ \text{d}x_{n} \end{array}\right].$$

The matrix on the right hand side is the Jacobian of the vector field. Replacing infinitesimal changes with finite perturbations, the above equation becomes:

Δf=JΔx.

In practice, a proportionality constant *c* is often added to the perturbation Δ***x*** to amplify the response Δ***f***. Furthermore, because vector fields are often learned in the PCA space, the perturbations in the *d*-dimensional gene space are first transformed to the *k*-dimensional PCA space by:

$$\text{Δ}x=Q^{⊤}(\text{Δ}y-\mu ).$$

where ***Q*** is the *d*-by-*k* PCA loading matrix, and ***μ*** is the mean of the PCA-transformed data. The response Δ***f*** can be transformed back to the PCA space:

Δg=QΔf+μ.

One can then use Δ***f***, a gene by cell matrix, to identify the strongest positive or negative responders of the genetic perturbation across cells (Figure 7B i).

Importantly, because Δ***f*** implies how each cell state will be affected after genetic perturbations, we can predict the cell fate trajectory under genetic perturbations by integrating the perturbation effects across cells over gene expression space, To visualize the cell fate trajectory, pairs of ***x*** and Δ***g*** are used in the same vein as the gene expression and RNA velocity vector to be further projected onto the UMAP or other low dimensional embeddings using the transition matrix (Bergen et al., 2020; La Manno et al., 2018) and then plotted with streamlines (Figure 7B).

#### Attaining tscRNA-seq dataset used in this study

The data deposited by the scEU-seq (Battich et al., 2020) study provided four species, namely unspliced unlabeled, unspliced labeled, spliced unlabeled, and spliced labeled RNA (*u*_*u*_, *u*_*l*_, *s*_*u*_, *s*_*l*_), and were retrieved via the GEO access ID GSE128365. Because scEU-seq manually separated labeled and unlabeled RNA, there is no need for a statistical estimation. However, the manual separation of labeled and unlabeled RNA may introduce potential cross-contamination, and the preparation of two libraries may lead to batch effects. Correction of those possible cross-contamination and batch effects represents an interesting future direction. Data for the sci-fate (Cao et al., 2020) and scNT-seq (Qiu et al., 2020) studies were obtained through direct communication with the authors before their publication. Custom statistical corrections, as reported in the original studies, were applied to the obtained datasets. Datasets for those studies can now also be downloaded via GEO access IDs GSE131351 and GSE141851, respectively.

#### Analysis details for the scNT-seq dataset

The wild-type and *Tet1/2/3* triple-knockout (TetTKO) datasets for studying the bidirectional transition between mESC pluripotent and totipotent state from (Qiu et al., 2020) were used in this study. The wild-type experiment used the degradation metabolic labeling scheme, whereas the TetTKO experiment used the one-shot metabolic labeling scheme. From both experiments, we obtained unspliced, spliced, labeled, and total RNA data for each gene in each cell. To estimate the absolute degradation rates for each gene in the wild-type dataset, we used the labeled and total RNA data and apply a curve fitting estimation approach (see ***Estimating absolute RNA velocity for metabolic labeling–based scRNA-seq experiments across various labeling strategies***) that builds on **Model 2** (Figure S2A), which does not consider splicing, and assumes a first-order decay for the RNA. We estimate the absolute splicing rate constant *β* by $\beta =\gamma /\tilde{\gamma }$, where *γ* and $\tilde{\gamma }$ are the absolute degradation rate constant (estimated using the curve fitting method) and the relative degradation rate constant (estimated from the spliced and unspliced RNA in the same dataset), respectively. Absolute splicing and degradation rate constants for each gene were then used for absolute RNA velocity calculation, velocity projection to 2D UMAP space of spliced RNA, vector field reconstruction (in the top 30 PC space), differential geometry analyses (e.g., Jacobian analysis), etc.

For the TetTKO dataset, we used the labeled and total RNA data to estimate absolute transcription and degradation rate constants using the “one-shot” method, which explicitly considers the time of the RNA metabolic labeling. Then we calculate the absolute total RNA velocity using the estimated transcription and degradation rate constants. Note that the transcription rates calculated here were cell- and gene-dependent (i.e., they corresponded to a cell-by-gene matrix like the expression matrix). On the other hand, the spliced and unspliced RNA were used to estimate the relative degradation rate constants. Combining the relative and absolute degradation rate constants, we obtained the absolute splicing rate constant, which allows us to compute the absolute spliced RNA velocity. The absolute total RNA velocity or spliced RNA velocity was then projected to the total RNA-based or spliced RNA–based 2D UMAP and used for vector field reconstructions (in the top 30 PC space), differential geometry analyses (e.g. Jacobian analysis), etc.

To benchmark the performance of labeling vs. splicing based RNA velocity analyses for the neuronal activ- ity dataset, we provided scVelo with the unspliced and spliced RNA counts of 97 neuronal activity genes, as features from the original study. Similar to the hematopoietic tscRNA-seq dataset analyses, we closely followed scVelo’s tutorials to perform spliced RNA velocity estimation with either the deterministic, stochastic, or dynamical method. We reproduced what we have reported previously on the labeling RNA velocity analysis using ***dynamo*** with default parameters (Qiu et al., 2020). The streamline plots in Figure S3H, J were produced with either scVelo or ***dynamo***, as noted. The splicing/labeling kinetics–based phase plots of example gene *Fos* in Figure S3I, J, were all produced under the respective models from scVelo or the one-shot model from ***dynamo***.

#### Analysis details for the scEU-seq dataset

Both the kinetics and mixture labeling experiment datasets of the cell cycle study using human RPE-1 cell line from (Battich et al., 2020) were used in this study. The degradation labeling experiment dataset of the intestinal organoid study from (Battich et al., 2020) was also used. We retrieved unspliced unlabeled, unspliced labeled, spliced unlabeled, and spliced labeled RNA data (*u*_*u*_, *u*_*l*_, *s*_*u*_, *s*_*l*_) for each gene in each cell from all experiments which then gave us unspliced (*u*), spliced (*s*), labeled (*l*) and total (*r*) RNA data (*u* = *u*_*u*_ *+ u*_*l*_, *s* = *s*_*u*_ *+ s*_*l*_, *l* = *u*_*l*_ *+ s*_*l*_, *r* = *u* + *s*). We mainly focused on analyzing the kinetics and degradation labeling experiments, while demonstrating the generalizability of our estimation framework and revealing the high transcription rates for mitochondrial genes with the mixture labeling experiment. For the kinetics experiment, we used the labeled and total RNA data and the “two-step” method to estimate the absolute transcription (cell- and gene-dependent, as above) and degradation rate constants. We estimated absolute splicing rate constants similar to the previous section. With the absolute transcription, splicing, and degradation rate constants, we can obtain absolute unspliced, spliced, labeled (or new), and total RNA velocities. The absolute total RNA velocity or spliced RNA velocity was then projected to the total RNA–based or spliced RNA–based 2D UMAP, and are used for vector field reconstructions (in the top 30 PC space), differential geometry analyses (e.g., Jacobian calculation), etc. For the mixture experiment, which had a fixed time period that includes a variable initial kinetics experiment and later accompanying degradation experiment [Figure S7 from (Battich et al., 2020)], we used a curve fitting strategy under **Model 2** (Figure S2A) to estimate the transcription and degradation rate constants. For the degradation experiment, we used the same strategy as mentioned above for the degradation experiment data from scNT-seq.

##### Functional analysis of kinetic rates calculated from scNT-seq or scEU-seq studies

Recent studies showed that degradation is slower for human proteins than their mouse counterparts during both embryonic segmentation (Matsuda et al., 2020) and motor neuron differentiation (Rayon et al., 2020). Because we calculated the degradation and splicing rate constants in the mESCs cells and hRPE-1 cells with data from the scNT-seq (Qiu et al., 2020) and scEU-seq studies (Battich et al., 2020) respectively, we can compare the degradation and splicing rate constants between human and mouse ortholog genes. The database of human and mouse ortholog genes was retrieved from ensembl bioMart (Smedley et al., 2015).

We also tested whether genes with high or low splicing and transcription rate constants are enriched for particular biological pathways. For the mESC degradation study, we compared the cumulative distri- bution of the degradation and splicing rate constants from housekeeping genes and other genes. The database of housekeeping genes was retrieved from https://www.genomics-online.com/resources/16/5049/housekeeping-genes/. For the hRPE-1 kinetics study, we took the top 10% of genes with the fastest splicing and degradation rate constants, and then subject them to GO pathway enrichment analysis.

#### Analysis details for the sci-fate dataset

The new and total RNA data from (Cao et al., 2020) were analyzed in this study. The absolute transcription and degradation rate constants, as well as the associated absolute total RNA velocity were estimated with the “one-shot” model. Genes from the original study reported to be associated with cell-cycle and glucocorticoid receptor (GR) response (Supplementary Table 2 of (Cao et al., 2020)) were used for the separated and combined RNA velocity analyses. To formally test whether the cell-cycle progression is independent of GR response, we first reconstructed the vector field on the 4D PCA space or the 3D UMAP space that was reduced from the combined expression space with cell-cycle and glucocorticoid receptor (GR) response genes, using the corresponding projected cell state and velocity vector pairs. We then calculated the Jacobian between those UMAP or PCA components in each cell. Overall high-magnitude Jacobian values across cells indicate a strong coupling between those processes related to those components and vice versa. The first and second principal components were related to linear GR response, whereas the third and fourth principal components were related to the cell cycle process. The first UMAP space is related to the GR response, whereas the second and third were related to the cell cycle process.

#### Analysis details for the Kimmerling dataset

Successful reconstruction of the vector field function from transcriptomic data depends on whether the input datasets capture sufficient dynamical information and whether hidden variables such as proteomic and epigenetic states are redundant in specifying cell dynamics. To test this, we examined a dataset (Kimmerling et al., 2016) in which sisters/cousins from primary activated murine CD8+ T cells were captured and measured using a specifically designed microfluidics platform (Figure S4B). Because sister or cousin cells are generated from the same cell through one or two cell divisions, respectively, they should explore the expression space in a similar manner (Figure S4C). Indeed, the transcriptomic distances between sisters and cousins are both significantly lower than those of random cell pairs (Figure S4D). Moreover, the distances between transcriptome-wide spliced RNA states of cells are highly correlated with those of estimated RNA velocity, and even more so for the unspliced RNA states (Figure S4E). In addition, cells close in transcriptome state shared similar RNA velocity vectors, and neighbor cells that also happened to be sisters or cousins did not exhibit higher similarity (Figure S4F). These results indicate when hidden variable effects are not apparent in the system, as in this case, one may predict velocity via a vector field function once the transcriptomic state is known, namely, $\dot{x}(t)=f(x(t))$.

#### Analysis details of the HL60 cell differentiation datasets

##### Process clone barcode and build “cell linkages”

Based on the conserved sequences flanking the cellular barcodes (GBCs), we retrieved the GBCs sequence for all reads in each cell from the scSLAM-seq clone tracing experiment and formed a cell by barcode matrix in which each element corresponds to the number of reads for that barcode observed in that cell. After removing barcodes with low reads across cells, we calculated the Leivenstein distance between all pairs of the remaining barcodes and applied an affinity propagation clustering algorithm to group barcodes into 666 clone clusters and identify a barcode exemplar for each cluster. Because the clustering algorithm itself does not incorporate a hard distance threshold between barcodes belonging to this barcode cluster and the exemplar of this cluster, we used a custom script to iteratively search for barcodes that had a Levenstein distance > 3 from the cluster exemplar or any newly identified exemplars, and appended those as new barcode cluster exemplars in addition to the existing ones. This approach yielded 764 uniquely identified barcodes. On the cell level, most cells had only one barcode, but a few that had two or more. In order to identify only confident cell linkages in which two or more cells shared the same barcode and to avoid spurious linkages, we explicitly ignored cells processed at nearby wells of a 384-well plate that had the same barcode as clone cells (Figure S5D). Because the wells in those plates were extremely small, cross-contamination between nearby wells can occur, leading to spurious cell linkage. This was less an issue for transcriptome qualification because the amount of leakage relative to the entire transcriptome was relatively small.

##### Analyze the 10x and scSLAM-seq datasets

We used default parameters to preprocess the 10x data and the unspliced and spliced RNA data from the scSLAM-seq experiment, and then performed dimension reduction and estimated and projected relative RNA velocity to the UMAP space for both datasets. For the one-shot labeling data from the scSLAM-seq experiment, the “one-shot” method was used with default parameters to estimate the absolute transcription rate and degradation rate constants, which were then integrated with the splicing data to obtain absolute splicing rate constants, as well as absolute spliced and total RNA velocity. Scatterplots of marker gene expression of progenitors and neutrophil lineages, as well as streamline plots with cells colored by sample collection time points on UMAP space across all datasets (10x, splicing data, and labeling data from the scSLAM-seq clone tracing experiment) were used to visualize neutrophil lineage commitment.

##### Analysis details for the Weinreb hematopoiesis dataset

We used hematopoietic datasets from (Weinreb et al., 2018), which included three major experiments: an *in vitro* experiment in which HSCs were cultured in competent differentiation media; a cytokine perturbation experiment in which HSCs in different plates received different differentiation factors, such as MPO or EPO; and an *in vivo* experiment in which barcoded HSCs were first allowed to proliferate *in vitro* for 2 days and then transplanted into 10 irradiated host mice whose blood cells were later harvested at week 1 and 2. Both of the first two experiments were subject to clone tracking on days 0, 2, 4, and 6, and all experiments were sequenced via inDrop-seq. Although the sequencing depth was not high (only 600 genes on average), roughly 100,000 cells are sequenced in each experiment. We used *kb-python* (https://github.com/pachterlab/kb_python) to reprocess the data to obtain unspliced and spliced counts for each cell.

We first performed velocity analysis on those datasets using ***dynamo*** with default parameters; however, for all three datasets, this resulted in unexpected backward velocity flows from terminal cell types to undiffer- entiated cells, based on cell-type assignments from the original study (Weinreb et al., 2018). After carefully ruling out issues with RNA velocity estimation, we reached the conclusion that the shallow sequencing of this study was the culprit of the backward velocity flow. We noticed that such biologically conflicting results have been observed by others and circulated online. In fact, RNA velocity estimation is prone to be problematic if the intron capture is insufficient or biased, as in the case of shallow sequencing. Hence, we were motivated to develop a heuristic method that uses some prior (of broad cell lineage hierarchy) to filter genes whose expression kinetics does not follow clockwise dynamics on the spliced–unspliced RNA phase plane. This supervised method (see details below) was used to correct the relative RNA velocity estimation and vector field reconstructions for all three datasets (*in vitro*, cytokine perturbation, and the *in vivo* experiment).

#### Details of the analysis of the human hematopoiesis tscRNA-seq dataset

To maximize the representation of known hematopoietic genes and thus improve the dimension reduction and other analyses, we first used the new RNA to select feature genes based on high variance, and then specifically appended a list of about 150 known hematopoietic genes compiled from multiple sources (Krumsiek et al., 2011; Paul et al., 2016; Weinreb et al., 2020) to establish our final feature gene set. This set of genes was then used for PCA denoising on the size factor–normalized and log1p-transformed data of new RNA counts, followed by UMAP projection (McInnes et al., 2018). The resultant UMAP embedding was used for all other downstream analyses, including the spliced RNA–based RNA velocity analyses.

The splicing data (spliced and unspliced RNA) from the combined adata object that comprises both cells collected on days 4 and 7, generated with ***dynast***, were used to perform conventional splicing kinetics–based RNA velocity analyses, using all methods implemented in both scVelo (version: 0.2.3) and ***dynamo***. Specifically, we closely followed the tutorials from scVelo to perform spliced RNA velocity estimation with either the deterministic, stochastic or dynamical model method. Similarly, we also performed spliced RNA velocity estimation based on either deterministic, stochastic or negative binomial distribution method using ***dynamo*** with default parameters. For the purpose of comparing the RNA velocity flow across tools and methods, we universally projected gene-wise RNA velocity vectors to the same UMAP space constructed with the new RNA expression (see above) in each tool for each method. To make Figure 3B, SI3C, we used ***dynamo*** to create all the streamline plots so that the color key of cell types and other aesthetics was used consistently for all tool and method combinations used for velocity estimation. Both the splicing RNA velocity plot in Figure 3B and splicing kinetics–based phase plot of example gene *PF4* in Figure 3E were built under the state-of-art dynamical model from scVelo.

To demonstrate the correction strategy of the splicing RNA velocity with ***dynamo***, we specifically feeded into ***dynamo*** the RNA velocity result obtained from the most sophisticated dynamical model in scVelo. We supplied the established hematopoietic lineage hierarchy information to the dyn.tl.confident_cell_velocities function in ***dynamo***. This function scores each gene based on the agreement of its behavior in the splicing phase diagram with the input lineage hierarchy priors (see ***Correcting RNA velocity flow by removing genes with low gene-wise confidence in the phase plane***). By default, all genes with the confidence score above 0.8 are used to re-project into low dimensional embeddings, which is further used to create an RNA velocity streamline plot as shown in Figure S3G. We also plotted the distribution of the confidence score for 316 velocity genes extracted from scVelo’s dynamical model (Figure S3E), as well as the boxplot of (only 43) genes that passed the filtering (Figure S3F).

The one-shot labeling model from ***dynamo*** was used to estimate absolute total RNA velocities on the labeling data (new and total RNA). Because we quantified both the labeling and splicing information, we used the second formula $\dot{r}=\alpha -\gamma s$ that involves both splicing and labeling data to define total RNA velocity. The high-dimensional velocity vectors were projected to two-dimensional UMAP space and visualized with the streamline plot, using ***dynamo*** with default parameters (Figure 3B). Similarly, the total RNA velocity plot in Figure 3D and total RNA phase diagram in Figure 3E for example gene *PF4* were generated using ***dynamo*** with default settings.

Pairs of the cell state and the velocity vector for that state, projected in either top-30 PC space or two-dimensional UMAP space, were used to reconstruct continuous vector field functions in ***dynamo*** with default parameters. As the dimension increases, the confidence of fixed point identification deteriorates, so we used the vector field constructed in the UMAP space to search for fixed points and associated them with stable hematopoietic cell types in our data. Furthermore, to ensure the full coverage of all fixed points, we increased the initial sample points from the default of 25 to 250 when searching for the fixed points. In the end, we manually selected the six most confident fixed points associated with each stable cell type, namely, HSCs, Meg, Ery, Bas, Mon, and Neu lineage cells. The type (repulsor or attractor) and confidence of fixed points were simultaneously identified and calculated. These results were then represented as a topography plot as shown in Figure 5B.

We used the vector field reconstructed from the UMAP space to build a lineage tree of hematopoiesis (Figure 5C). Specifically, we first estimated a weighted transition graph between cell types by calculating the fraction of vector field integration paths that starts from the sampled cell states (by default, 100 cells per cell type) of a particular cell type that passes through the middle of the cell states domain of another cell type. This cell-type transition graph was pruned and used to identify the shortest paths from the repulsory (HSC, based on the type of the corresponding fixed points) to absorbing cell types (Meg, Ery, Bas, Mon and Neu lineage cells) to form the final lineage tree. The pruning was achieved by simply restricting transitions to cell types that are adjacent in low dimensional gene expression space, such as the UMAP space. Note that we deliberately included a transition from the GMP-like cell state to the Bas lineage in Figure 5C to reflect this marginal transition, in addition to the dominant transition from the MEP-like cell to the Bas lineage.

We used the Hodge decomposition algorithm from (Maehara and Ohkawa, 2019) to estimate the pseudotime, relying on a directional transition graph computed during the RNA velocity projection with the default cosine kernel. This RNA velocity and vector field–based pseudotime was then used as the *x*-axis to visualize the timing of appearance of different lineages in Figure 5D. We also calculated the analytical acceleration vector in the PCA space for each cell with the PCA-based vector field, which was further projected back to the original gene expression space. We plotted the length of the acceleration vector in each cell on UMAP space (Figure 5E).

The analytical Jacobian matrix for each cell in the PCA space was calculated from the PCA-based vector field, which was further projected back to gene-wise space to enable a series of functional analyses. First, we calculated the Jacobian for *FLI1* (master regulator of Meg lineage) and *KLF1* (master regulator of Ery lineage) and visualized the Jacobian elements of *FLI1*’s self-activation and the mutual inhibition between *FLI1* and *KLF1* in each cell on the UMAP space (Figure 5F). Similarly, we also used the Jacobian analyses to compile a minimal network of the commitment of the Bas lineage based on the identified switch genes (see next paragraph) of the Bas lineage. We visualized the Jacobian elements corresponding to each interaction of the minimal network in Figure 5G and Figure S6E. For the canonical network motif PU.1–GATA1, we plotted the magnitude of self-activation and mutual inhibition of this motif across cells on the gene expression space of *PU.1* and *GATA1* in Figure 5I iii. To extract quantitative insight about the regulatory functions, we first plotted distributions of the four Jacobian elements versus expression of each gene with the so-called response heatmap, adapted from Scribe (Qiu et al., 2020b) (Figure 5I v, SI6G, H). We further fit the four Jacobians with either the active or inhibitory Hill equations (see ***Estimating kinetic parameters by fitting the Jacobian vs. expression curve***).

We relied on the Jacobian matrix ***J*** for each cell to identify toggle-switch gene pairs that mutually inhibit each other. To identify the toggle-switch gene pairs, we defined a *d*-by-*d* matrix ***K*** for each cell, where *d* is the dimension of the gene expression space, such that ***K*** = ***JJ***^**⏉**^. We further define:

$$L_{ij}=K_{ij}\left[J_{ij}<0\right]\left[J_{ji}<0\right],$$

where [*P*] is the Iverson bracket, which outputs 1 if the statement *P* is true and otherwise 0. The matrix *L* is used in the same way as the Jacobian matrix to perform the “interaction ranking” with absolute values but to identify the top toggle-switch gene pairs (see ***Ranking genes based on differential geometrical***). Intuitively, the preprocessing described here ensures that the gene pair with strongest mutual inhibitions (negative Jacobian values) will be ranked the highest. Note that the two Iverson brackets are used to filter out positive interactions. Also note that because *L* is a symmetric matrix, only the interactions above the main diagonal are needed for ranking.

##### Analysis details of the *in silico* perturbation

We used the dyn.vf.perturbation function to perform *in silico* perturbation and visualize the cell fate diversion with streamlines projected from the perturbation effect vectors (***In silico perturbation to predict gene-wise perturbation effects and cell fate diversions***). We suppressed *SPI1* (setting expression to −100), *GATA1* (setting expression to −100), and both *SPI1* (setting expression to −15, because repression of *GATA1* by *SPI1* is much stronger than the reverse interaction) and *GATA1* (setting expression to −100) and visualized the resultant streamline plots based on the perturbation effect vectors (Figure 7B i–iii). Similarly, we simulated the cell fate outcomes after activation of *KLF1* (setting expression to 100), suppression of *HLF1* (setting expression to −100), and triple activation of *GATA1*, *KLF1*, and *TAL1* (setting expression to 100 for all genes in all cells), as shown in the streamline plots of (Figure 7B vi–vi).

### QUANTIFICATION AND STATISTICAL ANALYSIS

Mann–Whitney–Wilcoxon two-sided tests with Bonferroni correction are used to compare the distribution differences in Figure S4D, F, as well as Figure S5F. The default hypergeometric test, from *gseapy* for GO enrichment analysis is used in Figure S2F.

## Supplementary Material

Supplemental Video Supplementary Animation, related to Figure 4:
Vector field animation that visualizes the long trajectories of single cells of human hematopoesis.

Fig SI1 Figure S1: Various ways to quantify expression dynamics and additional differential geometry analyses of vector fields, related to Figure 1. A. Gene expression (both *x*_1_ and *x*_2_) dynamics along the indicated trajectories in Figure 1A4. **1**) Gene expression quickly decreases, **2**) while velocity rapidly approaches 0 over time. Taking the derivative of the expression or velocity with respect to time along the indicated trajectory gives velocity **1**) or acceleration **2**), respectively, represented by red arrows. **3**) Increasing the gene expression linearly decreases the velocity of the other genes. **4**) The velocity of gene *x*_1_ positively correlates with that of *x*_2_, but with different strengths across the three trajectories.
B. The Jacobian of ∂*f*_1_/∂*x*_2_ (**left**), ∂*f*_2_/∂*x*_2_ (**right**) along the horizontal dashed line indicated in Figure 1 A4. Two other symmetric Jacobian elements, ∂*f*_2_/∂*x*_1_ (**left**), ∂*f*_1_/∂*x*_1_, are shown in Figure 1C.
C. Heatmaps of the curl (defined only in two or three dimensions ∇×*f* = ∂*f*_2_/∂*x*_1_ − ∂*f*_1_/∂*x*_2_ and divergence (∇·*f* = ∂*f*_1_/∂*x*_1_ + ∂*f*_2_/∂*x*_2_) landscapes in the phase space of the two-gene system.

Fig SI2 Figure S2: Comprehensive expression kinetics estimation framework in ***dynamo*** and global analyses of transcription, splicing, and degradation rate constants, related to Figure 2. A. Three main models of cscRNA-seq data (**Model 1**) and tscRNA-seq data that do not incorporate splicing (**Model 2**) or do (**Model 3**).
B. Estimating RNA degradation and splicing rates with data from degradation or kinetics labeling tscRNA-seq experiments. Scatterplot of 1) degradation rates, *γ*, estimated from labeling data, and slopes of the unspliced–spliced phase plane, $\tilde{\gamma }$, estimated from splicing data of mouse ESC cells from the scNT-seq study (Qiu et al., 2020a) on the **left**, and 2) degradation rates *γ* and the splicing rate $\beta =\gamma /\tilde{\gamma }$ from the human RPE-1 cells from the scEU-seq study in the **middle**. The murine splicing rate constant (*β*) calculated based on scNT-seq data is generally higher than that for humans calculated based on scEU-seq data (**right**).
C. Deterministic first-order decay model fitting of *Ank2* (slow degradation) and *Slc25a32* (fast degradation) chase data, using the ESC experiment data from the scNT-seq study (Qiu et al., 2020a).
D. Splicing rate constants (*β*) are in general much larger than the degradation rate constants (*γ*) in both the scNT-seq (**left**) and scEU-seq (**right**) dataset analysis based on the density plot.
E. Housekeeping genes tend to have faster splicing (**left**) but slower degradation (**right**) than other genes based on the cumulative distribution plot.
F. The top 10% genes from the scEU-seq dataset with highest splicing (**left**) or degradation (**right**) are enriched in transcription and cell cycle–related pathways.
G. Demonstration of estimating kinetic parameters from a mixture pulse-chase experiment from the scEU-seq study (Battich et al., 2020), using also its non-steady state model (Battich et al., 2020).
H. Genes with highest transcription rates are all mitochondrially encoded.
I. Degradation rates estimated from the non–steady-state model (Battich et al., 2020) of the mixture pulse-chase experiment are consistent with those estimated from the degradation experiment.

Fig SI3 Figure S3: ***Dynamo*** estimates RNA velocity more robustly and accurately on labeling data than splicing data, related to Figure 3. A. FACS plots showing human HSPCs (hematopoietic stem and progenitor cells) exiting from CD34^+^ compartment and first committing to the Meg (Megakaryocyte) lineage. CD34, CD33, and CD41a are markers for HSPCs, committed myeloid cells, and the Meg lineage, respectively.
B. Gene expression distribution of *PLEK*, *HBB*, *LMO4*, *MPO,* and *LYZ*, markers of the Meg, Ery (Erythrocyte), Bas (Basophil), Mon (Monocyte), and Neu (Neutrophil) lineages, respectively, on the UMAP space.
C. RNA velocity results of the splicing data from the hematopoietic tscRNA-seq experiment, obtained using scVelo and ***dynamo*** under different models/methods.
D. Corrected splicing RNA velocity flow via identification of reliable expression dynamics by scoring the velocity confidence of each gene in the phase plane when provided with a prior lineage relationship. See more discussion in STAR Methods.
E. Distribution of velocity confidence among all dynamical genes, identified via the dynamical model from scVelo. The dashed vertical line indicates the threshold (0.8) used by the correction algorithm to select confident velocity genes
F. Box plot of the number of non-confident vs. confident velocity genes.
G. Spliced RNA velocity flow after the correction.
H. RNA velocity flow on the splicing data from the neuronal activity scNT-seq dataset (Qiu et al., 2020a) using the deterministic, stochastic and dynamical models from scVelo.
I. Unsuccessful capture of introns and the constant transcription rate assumption causes most cells to have negative velocities, as evidenced by the fact that they are mostly under the estimated steady state line on the example gene, *Fos*.
J. Unbiased capture of nascent RNA via metabolic labeling and the gene/cell dependent RNA transcription rate modeling strategy give rise to correct RNA velocity flow of neuronal activity under KCl polarization.
K. Streamline plots with only GR-related genes (**left**), cycle–related genes (**middle**), and a combination of GR and cell cycle–related genes (**right**) for the sci-fate dataset (Cao et al., 2020b) on UMAP embedding. The right panel is the same as the right panel in Figure 2F but is annotated with inferred cell-cycle stages.
L. Cells with triple KO of *Tet 1/2/3* (TetTKO) are biased to differentiate into 2C-like cells, based on the splicing RNA velocity streamline plot produced with ***dynamo***.
M. Cells with TetTKO are biased to differentiate into 2C-like cells, based on the labeling RNA velocity streamline plot produced with ***dynamo***. Both **L** and **M** are the same as the middle and bottom panels from Figure 3H but are annotated with cell genotype information.

Fig SI4 Figure S4: ***Dynamo*** enables scalable and accurate reconstruction of vector field functions and characterization of vector field topologies, related to Figure 4. A. Existence of hidden variables may confound vector field reconstruction. The averaging intervals (shaded box, **right**) along the trajectory at points *x*_*a*_ and *x*_*b*_ have single-peaked distributions along *X* as well as the unmeasured *Y* (shaded density plot, **left**), whereas the interval at *x*_*c*_ has a two-peaked distribution for unmeasured *Y* (shaded density plot, **left**). Note the conditional distribution for *Y* at point *x*_*a*_ (*P*(*y*|*x*_*a*_)) is much wider than that at point *x*_*b*_ (*P*(*y*|*x*_*b*_)). Vector field reconstruction is expected to perform well when hidden variables from the system correlate with the observed variables (at *x*_*a*_), but less well when they are loosely coupled (*x*_*b*_ and *x*_*c*_). $\bar{y}$ corresponds to the mean of the distribution, and *K*(|*x* − *x*_*a*_|) is a fast-decay kernel function (for more detail, see discussion in STAR Methods).
B. Microfluidic platform design and experimental scheme to capture sisters/cousins from primary activated murine *CD8+* T-cell. Adapted from Figure 1 of (Kimmerling et al., 2016).
C. RNA velocity streamline plot of cells on UMAP embedding. Cells are colored by lineage groups (i.e., sister or cousin cells) of single cells.
D. Boxplot of the expression distance distribution (in the PCA space) of sister and cousin cell pairs, as well as that of random cell pairs. Mann–Whitney–Wilcoxon test (two-sided) was used to calculate the p-value between groups. ****: *p* ≤ 10^−4^.
E. Scatterplots of the distance of spliced RNA expression states of single cells vs. the distance of unspliced RNA expression states, and vs. that of RNA velocity vectors of single cells show strong correlations. Distances were calculated for any pairs of cells in PCA space. R-squared value (*R*^2^) is shown for each panel.
F. Distances between first-nearest neighbor cells show no difference among cells from the same or different linkages. Mann–Whitney–Wilcoxon test (two-sided) was used to calculate the p-value between groups. **: *p* ≤ 0.01.
G. Pairwise scatterplots of estimated and analytical Jacobian elements (indicated by the notation for each column) from corresponding cells (**Left**) and the identified outlier cells on the gene expression space of *x*_1_ and *x*_2_ (**right**). Accuracy of estimated Jacobian deteriorates in boundary regions of sampled cells due to insufficient and biased sampling.
H. Analytical differential geometric analyses enable nearly 1000x faster computation than state-of-the-art numeric algorithms (*numdifftools*).
I. Cosine correlation or RMSE between estimated vector field quantities (velocity, Jacobian, acceleration, divergence, curl, curvature and pseudotime) under different cell downsampling depth, noise level, and values of various parameters (*a, theta.* See **S** STAR Methods) of ***dynamo***’s vector field reconstruction algorithm and corresponding ground truths. The analytical velocity, Jacobian, acceleration, divergence, curl, and curvature of the simulated toggle-switch model are used as the ground truth. For pseudotime, the value calculated with default parameters is used as ground truth. The blue lines correspond to the linear regression fittings, and the shading surrounding the lines indicates the size of the confidence interval for the regression estimate (95%). *X*-axis is in log-scale. Each row is associated with the indicated benchmarks annotated above the corresponding horizontal line.

Fig SI5 Figure S5. Vector field–based cell fate predictions are validated by clonally related single cells, related Figure 4. A. Clonity of cells, which were sequentially sampled at different time points, is inferred based on the static barcodes (**left**) and is used to validate the vector field prediction (the red line, **right**).
B. Experimental schemes of conventional 10x Chromium–based scRNA-seq (**top**) and plate-based metabolic labeling scRNA-seq (scSLAM-seq or NASC-seq) coupled with sequential clonal cell tracking via lentivirus lineage barcodes (**bottom**) for neutrophil fate commitment of HL60 cells under ATRA treatment.
C. RNA velocity streamline plot of UMAP embedding based on labeling data from the scRNA-seq experiment reveals neutrophil-lineage commitment. From left to right, the cells on the streamline plot are colored with experimental time, progenitor marker (*CD38*) and neutrophil marker (*CD11b* or *ITGAM*) expression, respectively.
D. Layout of an example 384-well plate and locations of clonally related cells.
E. Forty confident clone-related linkages across different days among 944 cells from the scSLAM-seq experiment.
F. Boxplot of minimal distance of clone cells in later time points to the vector field prediction trajectory and the distance of random cells from the same day. Mann–Whitney–Wilcoxon test (two-sided) was used to calculate the p-value between groups. ****: *p* <= 1*e* − 04.
G. RNA velocity streamline plot of SPRING embedding (embedding is from (Weinreb et al., 2020)) reveals lineage hierarchy from murine hematopoietic stem cells to myeloid (megakaryocytes, erythroids, mast cells, basophil, eosphil, neutrophil, monocytes, dendritic cells, etc.) and lymphoid lineages for *in vivo* and *in vitro* systems. Cells are colored by the cell type identity.
H. A majority of cells from the same clone are biased towards a specific lineage or remain in the undifferentiated cell state. In the heatmap, row, column, and color correspond to a particular clone of cells, a particular cell lineage, and the probability that cells eventually commit to a cell lineage (or maintain the undifferentiated cell state).
I. Comparing vector field–based lineage fate predictions with other state-of-the-art methods. Smoothed clone fate (red dot) is the prediction based on clone barcodes. PBA, WOT, and fateID (black dots) are predictions based on other state-of-art algorithms that do not use velocity information. Those first four methods are from the original study (Weinreb et al., 2020). Predictions for all cells (fifth and sixth items) or the same subset of cells (seventh and eighth items) from the original studies are presented. Both PCA embedding, and SPRING embedding based vector fields from the original study were used for prediction.

Fig SI6 Figure S6: Differential geometry analyses of the reconstructed vector field function with ***dynamo*** predict regulatory mechanisms of hematopoiesis, related to Figure 5. A. The reconstructed vector field, and identified fixed points of hematopoiesis from the hematopoietic tscRNA-seq dataset. Cells are colored based on vector field–based pseudotime, which is calculated based on the RNA velocity transition matrix.
B. Megakaryocytes have the largest RNA speed (velocity magnitude) among all cell types.
C. Earlier expression (locally smoothed total RNA expression, *M*_*t*_, same as below, see more at STAR Methods) of *FLI1* (Meg lineage master regulator) relative to *KLF1* (Ery lineage master regulator) in progenitors.
D. Expression of top switch genes, *RUNX1* and *CEBPA*, for the Meg/Ery vs. Bas lineage bifurcation.
E. Jacobian analyses of the remaining regulatory interactions of the minimal network (Figure 5G iv) of the Bas lineage, except for the repression of *RUNX1* and *GATA2* by *CEBPA* in Figure 5G ii, iii.
F. Expression of *SPI1* and *GATA1*, GMP and MEP master regulators, respectively, in the UMAP embedding.
G. Transcription rate (*α*) analyses of the PU.1/SPI1–GATA1 network motif across all cells. i) Transcription rates of *SPI1* and *GATA1* in the *SPI1* and *GATA1* expression space. ii) Similar to i) (each column of the i-th subpanel matches with that of the ii-th subpanel), but replaced with a *response* heatmap (see STAR Methods).
H. Fitting the function of Jacobian vs. gene expression with derivatives of a simplistic inhibitory or activation Hill equation corresponds to mutual inhibition (first two subpanels) or self-activation (second two subpanels), respectively. White dashed line corresponds to the zero Jacobian value. The blue stars at each *x*-axis grid point correspond to the weighted mean of the Jacobian values for that point. The blue solid lines are the resultant fittings for the Jacobian. For more details, see STAR Methods.
I. The velocity kinetic curves over gene expression changes of the corresponding fitted Hill equations of panel **H**. For more details, see STAR Methods.
J. Trajectory-wise analyses of the SPI1-GATA1 network motif along a trajectory from HSCs to the Mon lineage. From left to right and top to bottom: i) The trajectory from HSCs to the Mon lineage in the first two PCA components. Cells are colored by cell-type identities, whereas trajectories are colored by integration time (i.e. the time predicted from the vector field integration, unit: hours). ii) The switch kinetics of *SPI1* and *GATA1* along the integration path. iii) Repression of *GATA1* by *SPI1* (∂*f*_GATA1_/∂*x*_SPI1_), repression of *SPI1* by *GATA1* (∂*f*_SPI1_/∂*x*_GATA1_), and self-activation of *SPI1* (∂*f*_SPI1_/∂*x*_SPI1_) and *GATA1* (∂*f*_GATA1_/∂*x*_GATA1_) along the integration paths reveal linear or sigmoid-like, time-dependent interactions.
K. Plane-wise analyses of the SPI1–GATA1 network motif in a GMP-like state. Self-activation of *SPI1*, repression of *SPI1* by *GATA1* and of *GATA1* by *SPI1*, and self-activation of *GATA1* as a function of *SPI1* (x-axis) and *GATA1* (y-axis) expression.

Fig SI7 Figure S7: Least action path and *in silico* perturbation accurately predict optimal cell-fate transition, related to Figure 6. A. Predicted optimal dedifferentiation path (i.e., dedifferentiation LAP) from each of the terminal cell types to HSC in the UMAP embedding. Color of the node along the paths indicates the LAP transition time.
B. Same as above but for transdifferentiation paths (a.k.a transdifferentiation LAPs).
C. Transcriptomic kinetics along the LAP of the basophil lineage back to HSC.
D. The LAP time between any hematopoietic cell states. Megakaryocyte developmental LAP has the overall shortest time among all developmental LAPs. Developmental time is significantly smaller than that of reprogramming.
E. The cell fate transition matrix between stable hematopoietic cell types. The expression kinetics of the top three genes for each transition (from the cell type in that row to the cell type in that column) along the LAP are plotted as a function of the LAP transition time (Unit: hour).
F. Majority of TFs for known hematopoietic transitions are accurately prioritized by LAP predictions.

Supplementary Table 1 Supplementary Table 1, related to Figure 6:
Top toggle switch gene pairs identified for each hematopoietic cell fate bifurcation of the tscRNA-seq dataset.

Supplementary Table 2 Supplementary Table 2, related to Figure 6:
Key transcription factors involved in known hematopoietic cell fate transitions

## Figures and Tables

**Table 1:** Available estimation algorithms for each labeling strategy.

Labeling strategy	One-shot	Kinetics (pulse)	Degradation
**Model**	Model 2/3	Model 2/3	Model 2/3
**Has splicing**	With or without	With or without	With or without
**Time points**	Single time point	Multiple time points	Multiple time points
**Steady state assumption**	Yes	Yes or No	Yes or No
**Estimation**	“**One-shot**” method (without splicing); NB method (with or without splicing);	**“Two-step”** method (without splicing); NB method (with or without splicing); curve fitting (with or without);	**Curve fitting** (with or without splicing)
**Velocity**	Velocity_N/T/S/U if integrated with conventional RNA velocity, Velocity_N/T otherwise	Velocity_N/T/S/U if integrated with conventional RNA velocity, Velocity_N/T otherwise	Velocity_S if splicing is considered, none otherwise

**Bold** fonts in the “Estimation” row correspond to the recommended method.

**Key resources table:** 

REAGENT or RESOURCE	SOURCE	IDENTIFIER
Antibodies		
CD14	Biolegend	Cat#367117
CD11b (for scSLAM-seq)	Biolegend	Cat#301309
CD34	Biolegend	Cat#343608
CD33	BD	Cat#340474
CD11b (for scNT-seq)	BD	Cat#562793
		
Bacterial and virus strains		
N.A.		
		
Biological samples		
N.A		
		
Chemicals, peptides, and recombinant proteins		
ATRA	Sigma-Aldrich	Cat# R2625-100MG
4sU	Sigma	Cat# T4509-25MG
2,2,2-trifluoroethylamine	Sigma-Aldrich	Cat# 91692-5ML
sodium periodate	Sigma-Aldrich	Cat# 311448-5G
TE	Sigma-Aldrich	Cat# 93302-100ML
RiboLock RNase inhibitor	Thermo Scientific	Cat# EO0381
Maxima H Minus 5X RT buffer	Thermo Scientific	Cat# EP0751
dNTPs	NEB	Cat# N0447L
Maxima H Minus reverse transcriptase enzyme	Thermo Scientific	Cat# EP0751
exonuclease I	NEB	Cat# M0293L
exonuclease I buffer	NEB	Cat# M0293L
Blue buffer	Enzymatics	Cat# P7010-HC-L
Klenow exo	Enzymatics	Cat# P7010-HC-L
KAPA HiFi HS ReadyMix	Roche	Cat# 07958935001
AMPURE XP beads	Beckman Coulter	Cat# A63881
SYBR Green Dye	Lonza	Cat# 12001-796
		
Critical commercial assays		
BD sample tags	BD Bioscience	Cat# PN 633780
Nextera XT DNA Library Prep Kit	Illumina	Cat# FC-131-1096
Agilent 2100 Bioanalyzer High Sensitivity DNA kit	Agilent Technologies	Cat# 5067-4626 and 5067-4627
10x Chromium™ Single Cell 3’ v2	10x genomics	Cat# PN-120267
		
Deposited data		
		
		
Experimental models: Cell lines		
HL60 (female)	ATCC	ATCC® CCL-240™
human CD34+ hematopoietic stem and progenitor cells	Fred Hutchinson Cancer Research Center	N/A
		
Experimental models: Organisms/strains		
N.A.		
		
Oligonucleotides		
Template switch oligo: AAGCAGTGGTATCAACGCAGAGTGAATrGrGrG	This paper	N/A
TSO-N9 primer: /5SpC3/AAGCAGTGGTATCAACGCAGAGTGAAT(N1:25252525)(N1)(N1)(N1)(N1)(N1)(N1)(N1)(N1)	This paper	N/A
TSO-GAATG primer: /5SpC3/AAGCAGTGGTATCAACGCAGAGTGAATG	This paper	N/A
TSO-PCR primer: AAGCAGTGGTATCAACGCAGAGT	This paper	N/A
P5-TSO hybrid primer: AATGATACGGCGACCACCGAGATCTACACGCCTGTCCGCGGAAGCAGTGGTATCAA	This paper	N/A
Nextera N70X oligo	Buenrostro et al., 2015	N/A
		
		
Recombinant DNA		
N.A		
		
Software and algorithms		
***dynamo*** (version: 1.0.0)	This paper	https://github.com/aristoteleo/dynamo-release
Dynast (version: 0.1.0)	Will reported elsewhere	https://github.com/aristoteleo/dynast-release
scVelo (version: 0.2.4)	Bergen et al., 2020	https://github.com/theislab/scvelo
		
		
Other		
		

## Box 1: Differential Geometry of Vector Fields

In this work, we introduced *dynamical systems theory* and *differential geometry* analyses to single-cell genomics. A dynamical system describes the time evolution of a point in a geometry space, whereas differential geometry uses the techniques from calculus and algebra to study geometric problems (Marsden and Tromba 2003). A vector field function ***f***, takes coordinates ***x*** in a *d*-dimensional space as input and outputs a vector ***v*** in the same space, i.e. ***v***=***f***(***x***). With differentiable velocity vector field functions, we can move beyond velocity to high-order quantities, as discussed below. The discussion in this study focuses on the transcriptomic space; vector fields, however, can be generally applicable to other spaces, such as morphological, proteomic, or metabolic space.

Because ***f*** is a vector-valued multivariate function, a *d*-by-*d* matrix encoding its derivatives, called the *Jacobian*, is defined as:

$$J=\left[\begin{array}{cccc} \frac{\partial f_{1}}{\partial x_{1}} & \frac{\partial f_{1}}{\partial x_{2}} & \cdots & \frac{\partial f_{1}}{\partial x_{d}}\\ \frac{\partial f_{2}}{\partial x_{1}} & \frac{\partial f_{2}}{\partial x_{2}} & \cdots & \frac{\partial f_{2}}{\partial x_{d}}\\ \cdots & \cdots & \cdots & \cdots \\ \frac{\partial f_{d}}{\partial x_{1}} & \frac{\partial f_{d}}{\partial x_{2}} & \cdots & \frac{\partial f_{d}}{\partial x_{d}} \end{array}\right].$$

A Jacobian element ∂*f*_*i*_/∂*x*_*j*_ reflects how the velocity of *x*_*i*_ is impacted by changes in ^*x*^*j*. In the context of this study, a positive (or negative) value for activating (or inhibitory) regulation (Figure 1B). Moreover, the maximum of ∂*f*_*i*_/∂*x*_*j*_ indicates where gene *j* has the strongest effect (activation or inhibition) on gene *i* (Figure 1B, C, Figure S1B).

The trace of the Jacobian is the *divergence*:

$$\nabla \cdot f=\sum_{i=1}^{d}\frac{\partial f_{i}}{\partial x_{i}}=TrJ.$$

Divergence measures the degree of “outgoingness” at any point, summarized in Box Fig. 1A.

By definition, an attractor (repulsor) converges (diverges) in any direction and therefore, divergence can be used to discover potential attractors and repulsors (see STAR Methods for additional discussions). *Curl* is a quantity measuring the degree of rotation at a given point in the vector field, whose behavior is summarized in Box Fig. 1B. See STAR Methods for computational details. *Acceleration* measures the rate of change of velocity in terms of both its magnitude and direction, as shown in Box Fig. 1C (orange shade). *Curvature*, on the other hand, measures only the change in direction, as the velocity vector is normalized. Box Fig. 1C (green shade) illustrates how the acceleration can be decomposed into a tangential and a radial component, and the latter is connected to the curvature:

$$a=a_{t}+|\nu {|}^{2}\kappa .$$

Although acceleration and curvature are mathematically defined on streamlines, actual calculations can be done pointwise using only the velocity and the Jacobian evaluated at the points of interest, which provide the acceleration or curvature vector field (see STAR Methods).

## Box 2: Vector Field Function Learning in Reproducing Kernel Hilbert Space

The overall goal of vector field function learning is to find a vector-valued function ***f*** in the function space H consisting of all possible vector field functions, such that, trained by a sparse set of coordinate–velocity data pairs ${\left\{x_{i},v_{i}\right\}}_{i=1}^{n}$, ***f***(***x***) gives the velocity ***v*** at an arbitrary coordinate ***x*** as schemed in Box Fig. 2A.

The coordinates ***x***_*i*_ in the gene expression space are fed into vector field functions (***g***, ***h***, and ***q***) in the function space H, which output vectors in the same space. To distinguish the output vectors from the velocity vectors from the data, these vectors from the vector field functions are called “*evaluations*”. As shown in the rightmost panel in Box Fig. 2A, intuitively ***h*** is best when one compares its evaluations ***h***(***x***_*i*_) to the velocity data ***v***_*i*_. This comparison can be formally evaluated with a *loss functional* (a function of functions) **Φ**(***f***) that measures how close the evaluations of the vector field functions and the velocity data are.

In general, a function space may have an infinite number of functions, and the learning procedure involves singling out one function through variational analysis,

$$f^{*}=\underset{f\in H}{argmin}\text{Φ}(f).$$

It is nontrivial to minimize the above loss functional computationally with respect to functions in the function space H. Note that ***f*** is an object defined in the function space, whereas ***f(x)***, the evaluation of ***f*** at point ***x***, is an object in the gene expression space, an ${\mathbb{R}}^{d}$ vector space, the same space in which the velocity vectors lives. The diagram in Box Fig. 2B outlines the relationships of the vector space and the function space.

One such tool is the reproducing kernel Hilbert space (RKHS). As shown in Box Fig. 2C and defined in STAR Methods, in RKHS, the vector field is represented as a combination of kernel-weighted (yellow radial gradient) coefficient vectors. A key elegant feature of the reproducing kernel is that it both encodes all the “options” in the function space and determines how the functional inner product is performed. See the corresponding sections in STAR Methods and Supplementary information for computational implementations.

## Box 3: Predicting Optimal Cell Fate Transitions by Least Action Paths

The least action path (LAP) principle, first proposed as early as 1744 by Maupertuis (Williams 2005) and famously advocated by Feynman with his reformulation of quantum mechanics via the path integral of the classical Hamilton action (Feynman, Hibbs, and Styer 2010), has previously been used in predicting the optimal transition path of cell fate transition for simplistic and designed systems (J. Wang et al. 2011; Qiu, Ding, and Shi 2012; P. Wang et al. 2014). With the reconstructed continuous, differentiable vector field, one can extend the LAP approach to real datasets in transcriptomic space to computationally explore optimal paths for differentiation and reprogramming (dedifferentiation and transdifferentiation), which then helps identify key transcription factors whose expression levels vary strongest along these paths.

The transcriptomic vector field encodes dynamical information of paths connecting different cell types. Generally, given a starting and an end cell states, there are infinitely many possible paths connecting the two. With a generic dynamical equation $\dot{x}(t)=f(x(t))+\zeta (t)$, including stochastic noises *ζ*(*t*) (see STAR Methods), the probability for cells to take a path *x*(*t*) is related to the action, defined as (Freidlin and Wentzell 2012; Onsager and Machlup 1953; Maier and Stein 1997):

$$S_{T}(x)=\frac{1}{2}\int_{0}^{T}\text{d}t{\left(v(t)-f(x(t))\right)}^{⊤}D^{-1}(v(t)-f(x(t)),$$

where ***D*** is the diffusion coefficient matrix related to *ζ*, *T* is the traversal time, the time needed for a cell to traverse the path. Based on this definition, a path has zero action if it strictly follows a streamline of a vector field whose tangential velocity *v(t)* also equals the evaluated velocity of the vector field, whereas any deviation increases action.

Developmental paths that connect progenitors and stable cell types, such as HSCs and megakaryocytes (Fig. 6C), are characterized by vector field streamlines, where cells need to overcome little to no dynamical barrier. On the contrary, a reversed process, i.e., dedifferentiation, typically requires cells to migrate against the streamline, overcoming the developmental barrier to become progenitors and reacquire multipotency. Stable cell types are attractors that are separated by attractor barriers (Box 1, Fig. 1); during transdifferentiation processes, cells from one stable attractor overcome these barriers and transverse into another stable attractor. Transitions that overcome the dedifferentiation/transdifferentiation barriers driven by stochasticity are rare, as evidenced by extremely low experimental reprogramming efficiency, as low as 0.001 – 0.01% (Merkl et al. 2013). This is why reprogramming factors are generally needed, which reshape the developmental landscape in favor of the reprogramming transitions.

For *rare transitions*, the Freidlin–Wentzell theorem dictates that LAPs dominate among all possible transition paths (Freidlin and Wentzell 2012; Onsager and Machlup 1953; Maier and Stein 1997; Aurell and Sneppen 2002). Furthermore, the *transition time* for such rare events—the average waiting time for observing the transitions to take place, which is typically much longer than the *traversal time*—can be inferred approximately from the action of LAPs (see STAR Methods).

For spontaneous transitions between points that are connected by the vector field streamlines (e.g., from a repulsor to an adjacent attractor), the streamlines are all LAPs with zero action, and the above approximation no longer applies. Differentiation processes are typically spontaneous, and the action of a differentiation LAP cannot tell us any information on the transition rate. However, LAPs are still the most probable paths for cells to take, as they are optimized to follow the streamline of the vector field. The waiting time for the cell to initiate the transition is negligible in this case, so the transition time can be approximated by the traversal time of the LAP. See STAR Methods for the algorithmic details of LAP.
