# Supplementary material for: Mapping Transcriptomic Vector Fields of Single Cells
Source: Cell. Author manuscript; Available in PMC 2022 Jul 28. (PMC9332140; doi:10.1016/j.cell.2021.12.045)
Supplement: Fig SI1 — A. Gene expression (both x1 and x2) dynamics along the indicated trajectories in Figure 1A4. 1) Gene expression quickly decreases, 2) while velocity rapidly approaches 0 over time. Taking the derivative of the expression or velocity with respect to time along the indicated trajectory gives velocity 1) or acceleration 2), respectively, represented by red arrows. 3) Increasing the gene expression linearly decreases the velocity of the other genes. 4) The velocity of gene x1 positively correlates with that of x2, but with different strengths across the three trajectories. B. The Jacobian of ∂f1/∂x2 (left), ∂f2/∂x2 (right) along the horizontal dashed line indicated in Figure 1 A4. Two other symmetric Jacobian elements, ∂f2/∂x1 (left), ∂f1/∂x1, are shown in Figure 1C. C. Heatmaps of the curl (defined only in two or three dimensions ∇×f = ∂f2/∂x1 − ∂f1/∂x2 and divergence (∇·f = ∂f1/∂x1 + ∂f2/∂x2) landscapes in the phase space of the two-gene system. [file NIHMS1802444-supplement-Fig_SI1.pdf]

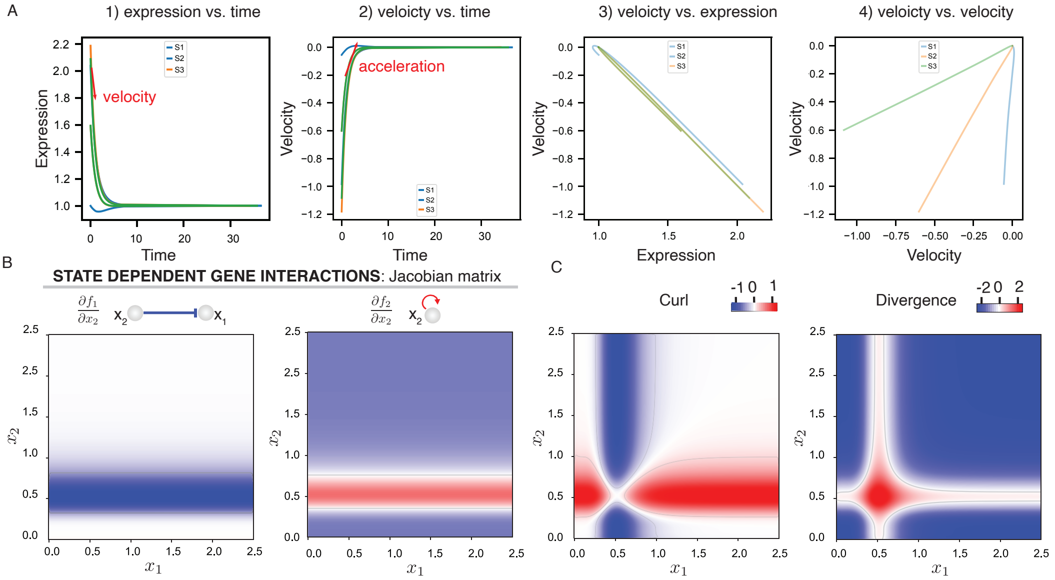
A
1) expression vs. time
2) veloicty vs. time
3) veloicty vs. expression
4) veloicty vs. velocity
Expression
Velocity
Time
velocity
acceleration
S1
S2
S3
B
STATE DEPENDENT GENE INTERACTIONS: Jacobian matrix
C
Curl
Divergence
