# Supplementary figures and images for: Mapping Transcriptomic Vector Fields of Single Cells
Source: Cell. Author manuscript; Available in PMC 2022 Jul 28. (PMC9332140; doi:10.1016/j.cell.2021.12.045)

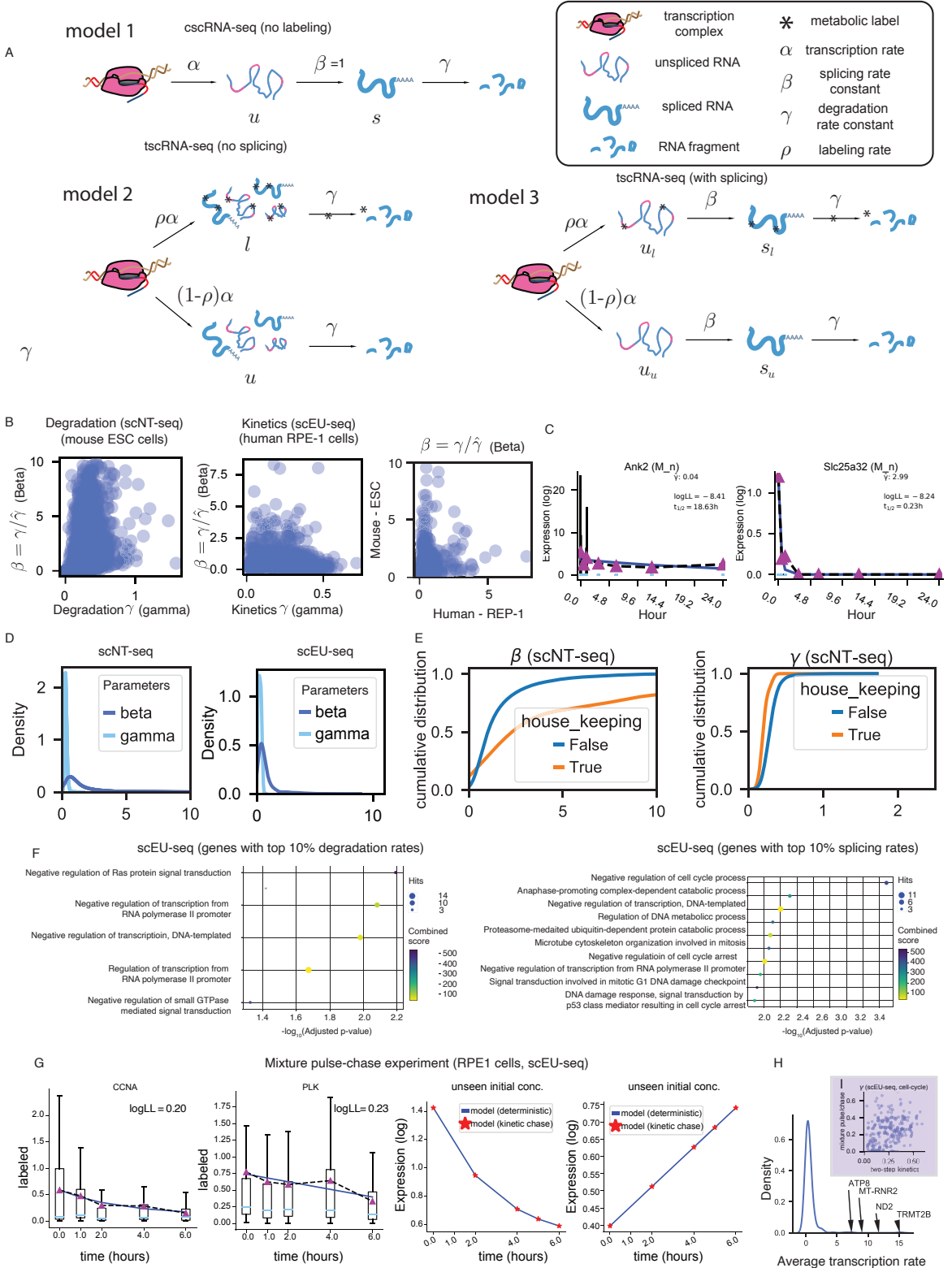

Supplement: Fig SI2 — A. Three main models of cscRNA-seq data (Model 1) and tscRNA-seq data that do not incorporate splicing (Model 2) or do (Model 3). B. Estimating RNA degradation and splicing rates with data from degradation or kinetics labeling tscRNA-seq experiments. Scatterplot of 1) degradation rates, γ, estimated from labeling data, and slopes of the unspliced–spliced phase plane, γ˜, estimated from splicing data of mouse ESC cells from the scNT-seq study (Qiu et al., 2020a) on the left, and 2) degradation rates γ and the splicing rate β=γ/γ˜ from the human RPE-1 cells from the scEU-seq study in the middle. The murine splicing rate constant (β) calculated based on scNT-seq data is generally higher than that for humans calculated based on scEU-seq data (right). C. Deterministic first-order decay model fitting of Ank2 (slow degradation) and Slc25a32 (fast degradation) chase data, using the ESC experiment data from the scNT-seq study (Qiu et al., 2020a). D. Splicing rate constants (β) are in general much larger than the degradation rate constants (γ) in both the scNT-seq (left) and scEU-seq (right) dataset analysis based on the density plot. E. Housekeeping genes tend to have faster splicing (left) but slower degradation (right) than other genes based on the cumulative distribution plot. F. The top 10% genes from the scEU-seq dataset with highest splicing (left) or degradation (right) are enriched in transcription and cell cycle–related pathways. G. Demonstration of estimating kinetic parameters from a mixture pulse-chase experiment from the scEU-seq study (Battich et al., 2020), using also its non-steady state model (Battich et al., 2020). H. Genes with highest transcription rates are all mitochondrially encoded. I. Degradation rates estimated from the non–steady-state model (Battich et al., 2020) of the mixture pulse-chase experiment are consistent with those estimated from the degradation experiment. [file NIHMS1802444-supplement-Fig_SI2.pdf]

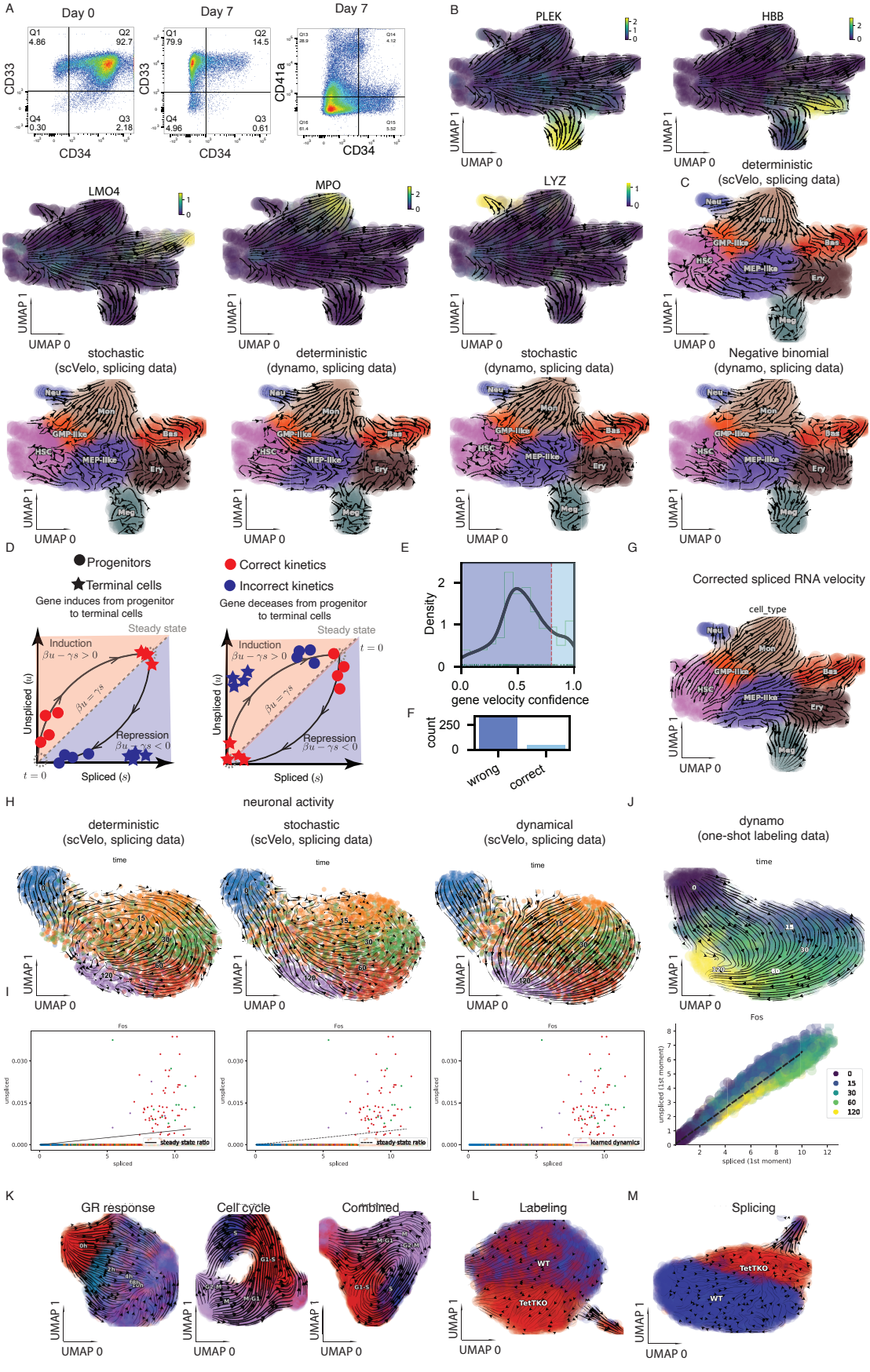

Supplement: Fig SI3 — A. FACS plots showing human HSPCs (hematopoietic stem and progenitor cells) exiting from CD34+ compartment and first committing to the Meg (Megakaryocyte) lineage. CD34, CD33, and CD41a are markers for HSPCs, committed myeloid cells, and the Meg lineage, respectively. B. Gene expression distribution of PLEK, HBB, LMO4, MPO, and LYZ, markers of the Meg, Ery (Erythrocyte), Bas (Basophil), Mon (Monocyte), and Neu (Neutrophil) lineages, respectively, on the UMAP space. C. RNA velocity results of the splicing data from the hematopoietic tscRNA-seq experiment, obtained using scVelo and dynamo under different models/methods. D. Corrected splicing RNA velocity flow via identification of reliable expression dynamics by scoring the velocity confidence of each gene in the phase plane when provided with a prior lineage relationship. See more discussion in STAR Methods. E. Distribution of velocity confidence among all dynamical genes, identified via the dynamical model from scVelo. The dashed vertical line indicates the threshold (0.8) used by the correction algorithm to select confident velocity genes F. Box plot of the number of non-confident vs. confident velocity genes. G. Spliced RNA velocity flow after the correction. H. RNA velocity flow on the splicing data from the neuronal activity scNT-seq dataset (Qiu et al., 2020a) using the deterministic, stochastic and dynamical models from scVelo. I. Unsuccessful capture of introns and the constant transcription rate assumption causes most cells to have negative velocities, as evidenced by the fact that they are mostly under the estimated steady state line on the example gene, Fos. J. Unbiased capture of nascent RNA via metabolic labeling and the gene/cell dependent RNA transcription rate modeling strategy give rise to correct RNA velocity flow of neuronal activity under KCl polarization. K. Streamline plots with only GR-related genes (left), cycle–related genes (middle), and a combination of GR and cell cycle–related genes (righ [file NIHMS1802444-supplement-Fig_SI3.pdf]

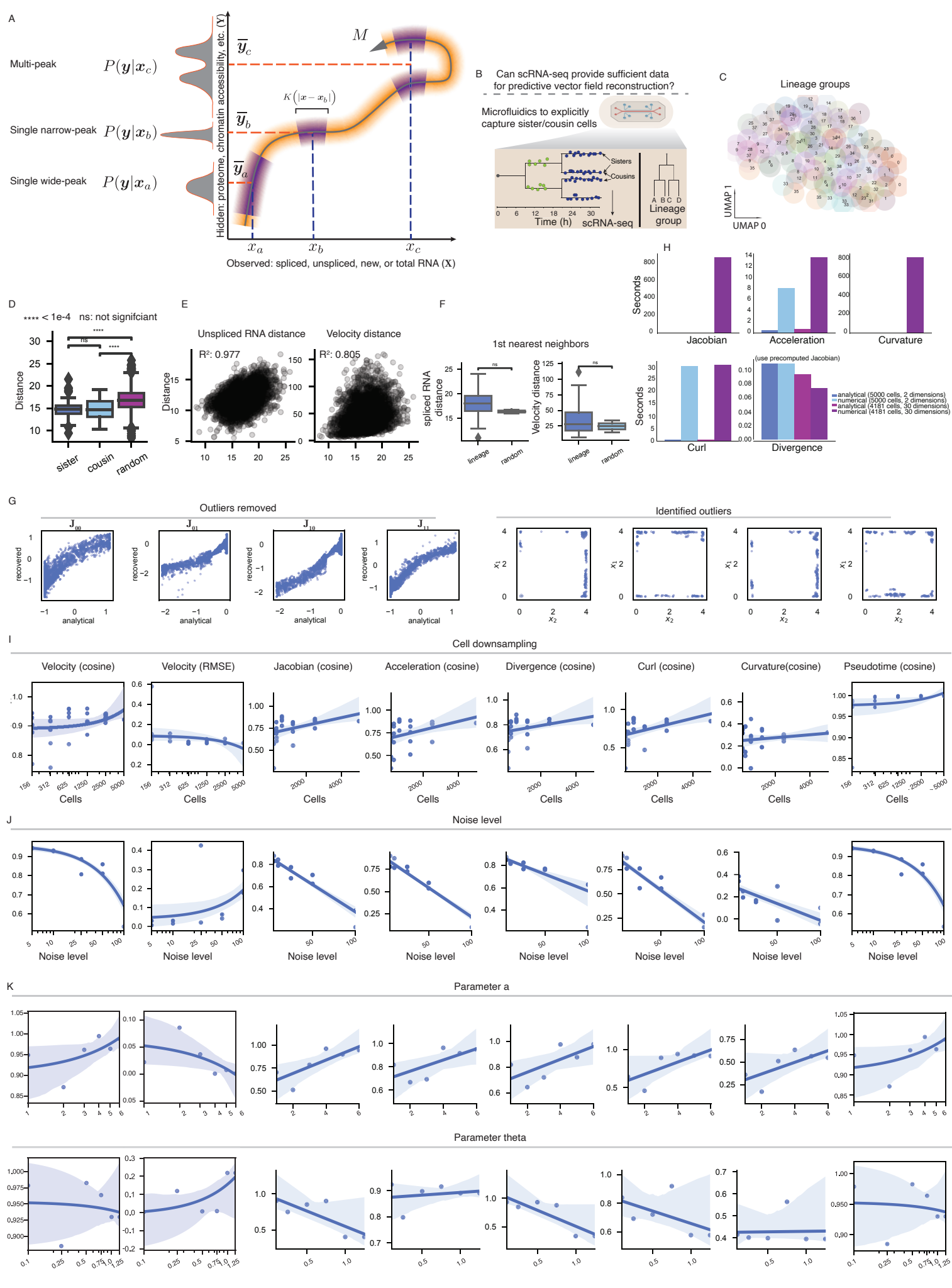

Supplement: Fig SI4 — A. Existence of hidden variables may confound vector field reconstruction. The averaging intervals (shaded box, right) along the trajectory at points xa and xb have single-peaked distributions along X as well as the unmeasured Y (shaded density plot, left), whereas the interval at xc has a two-peaked distribution for unmeasured Y (shaded density plot, left). Note the conditional distribution for Y at point xa (P(y|xa)) is much wider than that at point xb (P(y|xb)). Vector field reconstruction is expected to perform well when hidden variables from the system correlate with the observed variables (at xa), but less well when they are loosely coupled (xb and xc). y¯ corresponds to the mean of the distribution, and K(|x − xa|) is a fast-decay kernel function (for more detail, see discussion in STAR Methods). B. Microfluidic platform design and experimental scheme to capture sisters/cousins from primary activated murine CD8+ T-cell. Adapted from Figure 1 of (Kimmerling et al., 2016). C. RNA velocity streamline plot of cells on UMAP embedding. Cells are colored by lineage groups (i.e., sister or cousin cells) of single cells. D. Boxplot of the expression distance distribution (in the PCA space) of sister and cousin cell pairs, as well as that of random cell pairs. Mann–Whitney–Wilcoxon test (two-sided) was used to calculate the p-value between groups. ****: p ≤ 10−4. E. Scatterplots of the distance of spliced RNA expression states of single cells vs. the distance of unspliced RNA expression states, and vs. that of RNA velocity vectors of single cells show strong correlations. Distances were calculated for any pairs of cells in PCA space. R-squared value (R2) is shown for each panel. F. Distances between first-nearest neighbor cells show no difference among cells from the same or different linkages. Mann–Whitney–Wilcoxon test (two-sided) was used to calculate the p-value between groups. **: p ≤ 0.01. G. Pairwise scatterplots of estimated and analytical Jacobian elements (in [file NIHMS1802444-supplement-Fig_SI4.pdf]

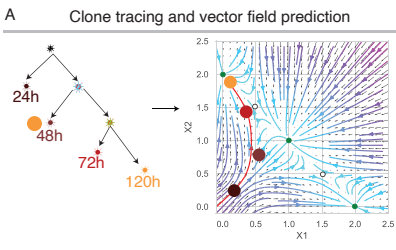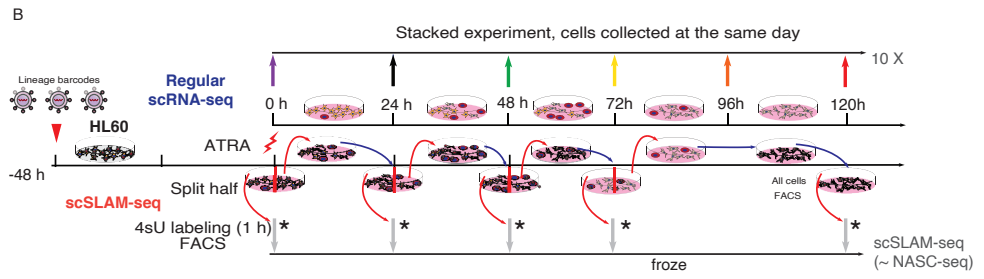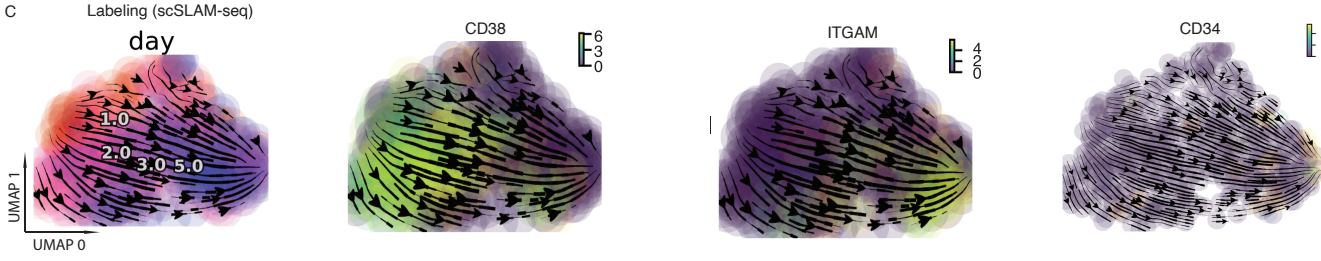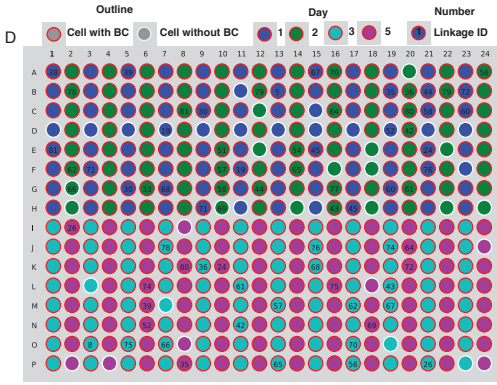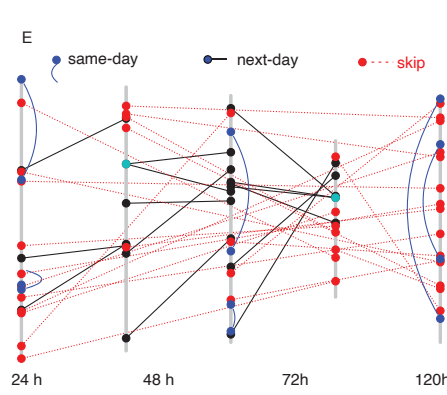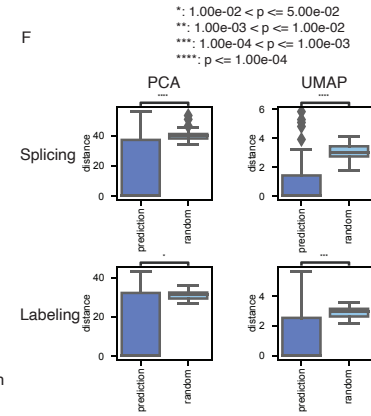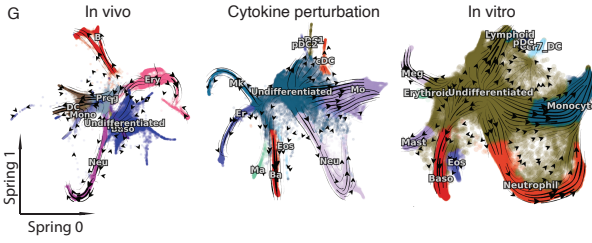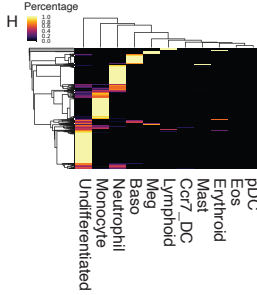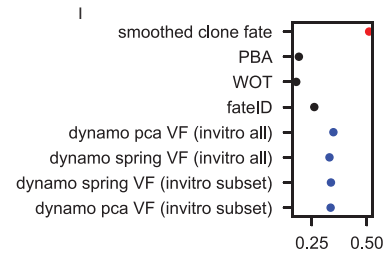

Supplement: Fig SI5 — A. Clonity of cells, which were sequentially sampled at different time points, is inferred based on the static barcodes (left) and is used to validate the vector field prediction (the red line, right). B. Experimental schemes of conventional 10x Chromium–based scRNA-seq (top) and plate-based metabolic labeling scRNA-seq (scSLAM-seq or NASC-seq) coupled with sequential clonal cell tracking via lentivirus lineage barcodes (bottom) for neutrophil fate commitment of HL60 cells under ATRA treatment. C. RNA velocity streamline plot of UMAP embedding based on labeling data from the scRNA-seq experiment reveals neutrophil-lineage commitment. From left to right, the cells on the streamline plot are colored with experimental time, progenitor marker (CD38) and neutrophil marker (CD11b or ITGAM) expression, respectively. D. Layout of an example 384-well plate and locations of clonally related cells. E. Forty confident clone-related linkages across different days among 944 cells from the scSLAM-seq experiment. F. Boxplot of minimal distance of clone cells in later time points to the vector field prediction trajectory and the distance of random cells from the same day. Mann–Whitney–Wilcoxon test (two-sided) was used to calculate the p-value between groups. ****: p <= 1e − 04. G. RNA velocity streamline plot of SPRING embedding (embedding is from (Weinreb et al., 2020)) reveals lineage hierarchy from murine hematopoietic stem cells to myeloid (megakaryocytes, erythroids, mast cells, basophil, eosphil, neutrophil, monocytes, dendritic cells, etc.) and lymphoid lineages for in vivo and in vitro systems. Cells are colored by the cell type identity. H. A majority of cells from the same clone are biased towards a specific lineage or remain in the undifferentiated cell state. In the heatmap, row, column, and color correspond to a particular clone of cells, a particular cell lineage, and the probability that cells eventually commit to a cell lineage (or maintain the undifferentiated cell [file NIHMS1802444-supplement-Fig_SI5.pdf]

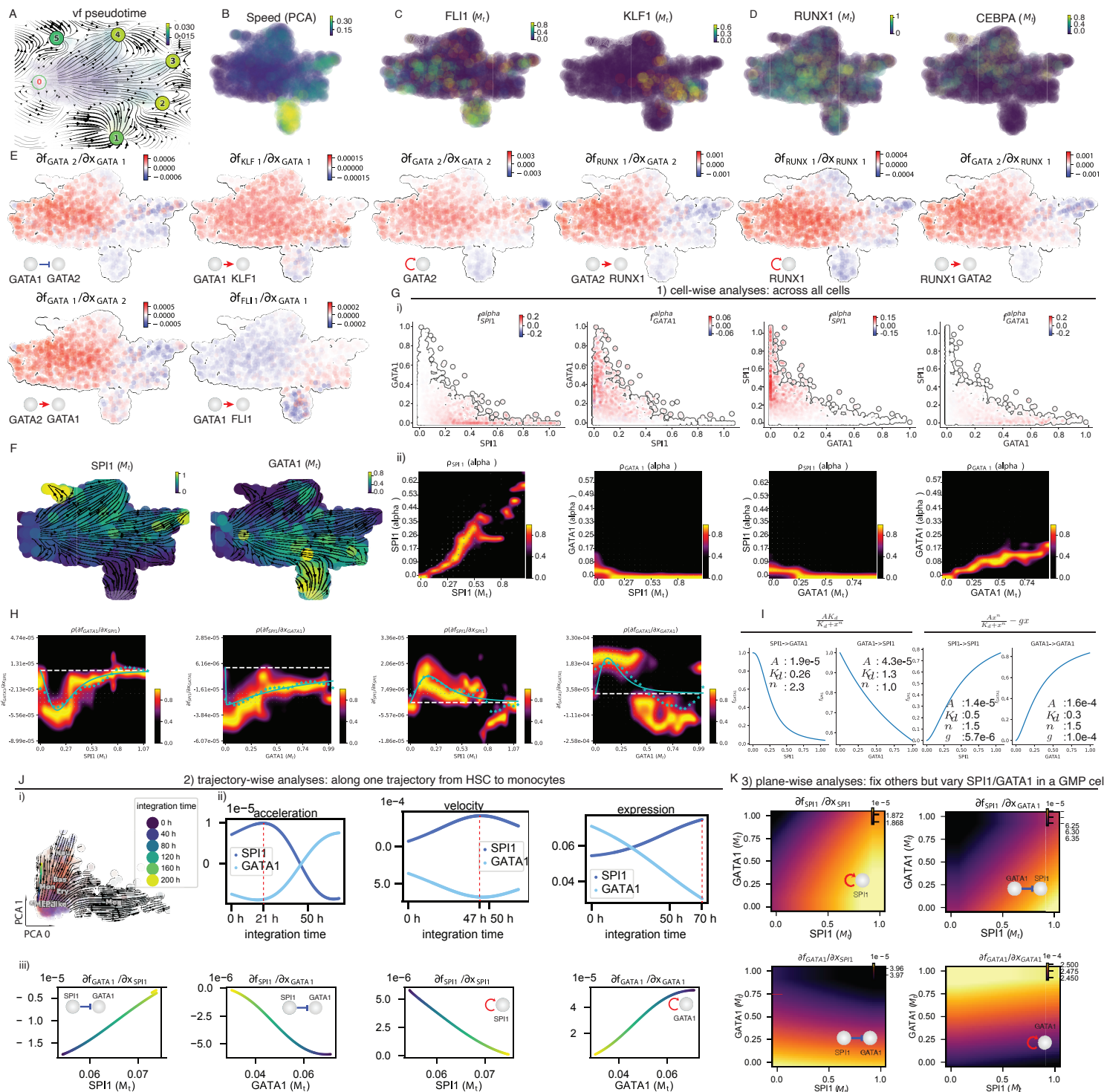

Supplement: Fig SI6 — A. The reconstructed vector field, and identified fixed points of hematopoiesis from the hematopoietic tscRNA-seq dataset. Cells are colored based on vector field–based pseudotime, which is calculated based on the RNA velocity transition matrix. B. Megakaryocytes have the largest RNA speed (velocity magnitude) among all cell types. C. Earlier expression (locally smoothed total RNA expression, Mt, same as below, see more at STAR Methods) of FLI1 (Meg lineage master regulator) relative to KLF1 (Ery lineage master regulator) in progenitors. D. Expression of top switch genes, RUNX1 and CEBPA, for the Meg/Ery vs. Bas lineage bifurcation. E. Jacobian analyses of the remaining regulatory interactions of the minimal network (Figure 5G iv) of the Bas lineage, except for the repression of RUNX1 and GATA2 by CEBPA in Figure 5G ii, iii. F. Expression of SPI1 and GATA1, GMP and MEP master regulators, respectively, in the UMAP embedding. G. Transcription rate (α) analyses of the PU.1/SPI1–GATA1 network motif across all cells. i) Transcription rates of SPI1 and GATA1 in the SPI1 and GATA1 expression space. ii) Similar to i) (each column of the i-th subpanel matches with that of the ii-th subpanel), but replaced with a response heatmap (see STAR Methods). H. Fitting the function of Jacobian vs. gene expression with derivatives of a simplistic inhibitory or activation Hill equation corresponds to mutual inhibition (first two subpanels) or self-activation (second two subpanels), respectively. White dashed line corresponds to the zero Jacobian value. The blue stars at each x-axis grid point correspond to the weighted mean of the Jacobian values for that point. The blue solid lines are the resultant fittings for the Jacobian. For more details, see STAR Methods. I. The velocity kinetic curves over gene expression changes of the corresponding fitted Hill equations of panel H. For more details, see STAR Methods. J. Trajectory-wise analyses of the SPI1-GATA1 network motif along a trajector [file NIHMS1802444-supplement-Fig_SI6.pdf]

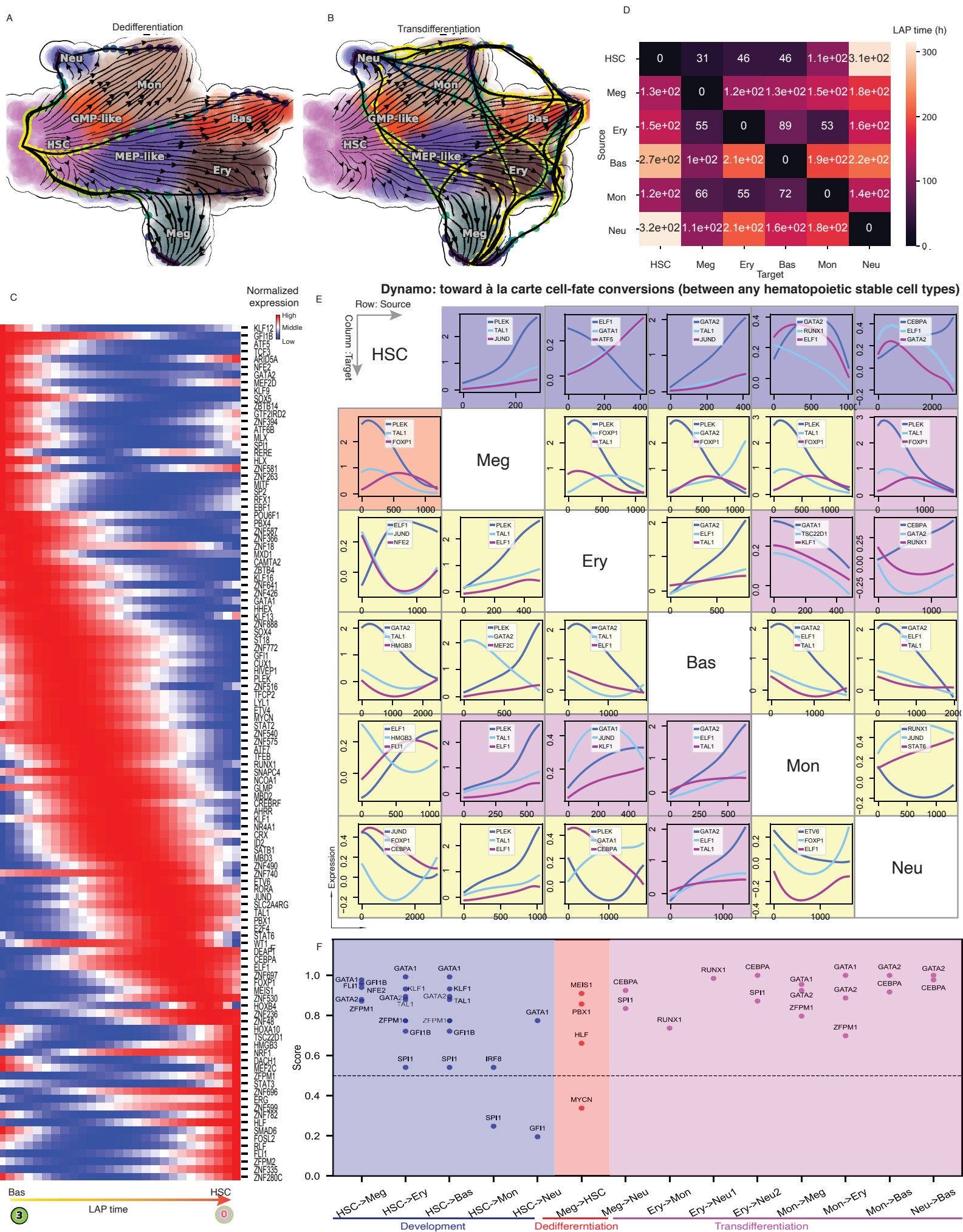

Supplement: Fig SI7 — A. Predicted optimal dedifferentiation path (i.e., dedifferentiation LAP) from each of the terminal cell types to HSC in the UMAP embedding. Color of the node along the paths indicates the LAP transition time. B. Same as above but for transdifferentiation paths (a.k.a transdifferentiation LAPs). C. Transcriptomic kinetics along the LAP of the basophil lineage back to HSC. D. The LAP time between any hematopoietic cell states. Megakaryocyte developmental LAP has the overall shortest time among all developmental LAPs. Developmental time is significantly smaller than that of reprogramming. E. The cell fate transition matrix between stable hematopoietic cell types. The expression kinetics of the top three genes for each transition (from the cell type in that row to the cell type in that column) along the LAP are plotted as a function of the LAP transition time (Unit: hour). F. Majority of TFs for known hematopoietic transitions are accurately prioritized by LAP predictions. [file NIHMS1802444-supplement-Fig_SI7.pdf]
